# Bioactive Oil Blend Nanoemulsion Attenuates Depression-like Behavior Through Modulation of Neuroimmune-Related Inflammatory Pathways in Experimental Rheumatoid Arthritis

**DOI:** 10.3390/life16091428

**Published:** 2026-08-27

**Authors:** Doha A. Mohamed, Hoda B. Mabrok, Hagar F. Elbakry, Marwa E. El-Shamarka, Rania A. Bassuoni

**Affiliations:** 1 Nutrition and Food Science Department, Food Industries and Nutrition Institute, National Research Centre, Dokki, Cairo 12622, Egypt; hoda.mabrok@gmail.com (H.B.M.); faridhagar79@yahoo.com (H.F.E.); raniabassuoni@yahoo.com (R.A.B.); 2 Toxicology and Narcotics Department, Medical Research and Clinical Studies Institute, National Research Centre, Dokki, Cairo 12622, Egypt; marwaelshamarka@gmail.com

**Keywords:** rheumatoid arthritis, depression-like behavior, oil blend nanoemulsion, network pharmacology, molecular docking, neuroinflammation, cholinergic pathway, agro-industrial by-products

## Abstract

**Background**: Rheumatoid arthritis (RA) is a chronic autoimmune disease frequently accompanied by depression, with persistent inflammation, oxidative stress, and neuroimmune dysfunction contributing to disease progression. This study evaluated the therapeutic efficacy of a Gum Arabic-stabilized bioactive oil blend nanoemulsion and investigated its underlying mechanisms using molecular docking and network pharmacology. **Methods**: A lyophilized nanoemulsion prepared from grape seed oil, wheat germ oil, and avocado peel oil was characterized for physicochemical properties and phytochemical composition. Female rats with Freund’s complete adjuvant-induced rheumatoid arthritis received the nanoemulsion at two doses. Paw inflammation, behavioral performance, inflammatory cytokines, oxidative stress biomarkers, acetylcholinesterase concentration, lipid profile, and liver and kidney function were evaluated. Molecular docking and network pharmacology analyses were performed to identify potential molecular targets and signaling pathways. **Results**: The nanoemulsion exhibited favorable physicochemical characteristics and was rich in phenolic compounds, flavonoids, phytosterols, tocopherols, and unsaturated fatty acids. Treatment significantly reduced paw inflammation, TNF-α, IL-6, malondialdehyde, and acetylcholinesterase while enhancing catalase activity, improving metabolic parameters, and alleviating depression-like behavior. Molecular docking demonstrated favorable binding of the major phytochemicals to TNF-α, IL-6, and acetylcholinesterase. Network pharmacology identified key therapeutic targets, including TNF, AKT1, PTGS2, IL6, STAT3, ESR1, and NR3C1, and revealed enrichment of inflammatory, oxidative stress, and neuroimmune signaling pathways. **Conclusions**: The Gum Arabic-stabilized bioactive oil blend nanoemulsion ameliorated rheumatoid arthritis-associated depression-like behavior through a multi-component–multi-target–multi-pathway mechanism involving coordinated regulation of inflammatory, oxidative stress, cholinergic, and neuroimmune pathways. These findings support its potential as a complementary nutraceutical strategy for rheumatoid arthritis and its associated neurobehavioral complications.

## 1. Introduction

Rheumatoid arthritis (RA) is a chronic autoimmune inflammatory disease affecting approximately 0.5–1% of the global population and remains one of the leading causes of disability and reduced quality of life worldwide [1,2]. Although RA primarily affects the synovial joints, it is now recognized as a systemic disease characterized by persistent inflammation, oxidative stress, metabolic disturbances, and a broad spectrum of extra-articular manifestations that collectively increase morbidity and mortality [1,2]. Despite substantial advances in disease-modifying antirheumatic drugs (DMARDs) and biologic therapies, many patients continue to experience persistent disease activity, incomplete remission, treatment resistance, or treatment-related adverse effects; this highlights the need for complementary therapeutic strategies capable of targeting multiple pathogenic mechanisms simultaneously.

Depression is among the most common and clinically significant extra-articular manifestations of RA, affecting approximately 20–40% of patients and substantially worsening quality of life, treatment adherence, and clinical outcomes [3]. In addition to its psychological burden, depression is associated with increased cardiovascular and all-cause mortality in patients with RA [4]. Patients with concurrent RA and depression generally experience greater pain intensity, higher disease activity, poorer functional status, and lower remission rates than those without depression [5]. Women are particularly vulnerable, with higher rates of depression and anxiety than men, making female experimental models especially relevant for investigating RA-associated neurobehavioral complications [6,7]. Moreover, fatigue, sleep disturbances, and disability further reinforce the bidirectional relationship between chronic inflammation and depression, creating a vicious cycle that contributes to disease progression and poorer clinical outcomes [8,9].

Current evidence indicates that RA-associated depression arises from shared biological mechanisms rather than representing only a psychological response to chronic pain. Persistent elevation of pro-inflammatory cytokines, particularly tumor necrosis factor-α (TNF-α) and interleukin-6 (IL-6), facilitates communication between peripheral inflammation and the central nervous system, leading to microglial activation, mitochondrial dysfunction, impaired synaptic plasticity, and depression-like behavior [1]. Multi-omics analyses further demonstrate coordinated neuroimmune and metabolic dysregulation across RA and depression, supporting the concept that these disorders share common pathogenic pathways [10]. Oxidative stress amplifies these processes through excessive reactive oxygen species generation, lipid peroxidation, and impairment of endogenous antioxidant defenses, with malondialdehyde (MDA) consistently elevated in both conditions [11]. Furthermore, dysregulation of the cholinergic anti-inflammatory pathway contributes to persistent inflammation and neurobehavioral impairment. Increased acetylcholinesterase (AChE) activity reduces acetylcholine availability, thereby weakening vagus nerve-mediated anti-inflammatory signaling and facilitating excessive cytokine production [12]. Collectively, these findings identify inflammatory, oxidative stress, and cholinergic pathways as attractive therapeutic targets for simultaneously attenuating systemic inflammation and depression-like behaviors.

Modern botanical formulations integrate the traditional concept of multi-component plant-based therapy with contemporary pharmaceutical technologies to improve efficacy, safety, and mechanistic understanding [13,14,15]. Rather than acting on a single molecular target, these formulations are designed to regulate multiple interconnected biological pathways through complementary bioactive constituents, and advanced delivery systems enhance their stability, bioavailability, and therapeutic performance [13,14]. This multi-target strategy is particularly relevant for complex disorders such as RA, where chronic inflammation, oxidative stress, immune dysregulation, and neurobehavioral alterations coexist.

Naturally occurring phytochemicals have gained considerable attention as multifunctional agents capable of modulating these interconnected pathological pathways. Polyphenols, flavonoids, phytosterols, tocopherols, and polyunsaturated fatty acids exhibit well-documented antioxidant, anti-inflammatory, immunomodulatory, and neuroprotective activity. Bioactive compounds such as catechin, quercetin, rutin, chlorogenic acid, and ferulic acid suppress inflammatory cytokine production, reduce oxidative stress, and improve neuronal function through regulation of NF-κB- and Nrf2-dependent signaling pathways [11]. Likewise, phytosterols and α-tocopherol contribute to membrane stabilization, immune regulation, and protection against oxidative damage [16,17,18], whereas polyunsaturated fatty acids promote the resolution of inflammation and preserve neuronal membrane integrity [19]. Nevertheless, the therapeutic potential of many lipophilic phytochemicals is limited by poor aqueous solubility, chemical instability, and low oral bioavailability.

Nanotechnology has emerged as an effective strategy to overcome these limitations by improving the stability, gastrointestinal absorption, bioaccessibility, and controlled delivery of natural bioactive compounds. Nanoemulsion systems protect lipophilic phytochemicals from degradation, enhance their gastrointestinal absorption and interaction with biological membranes, and ultimately improve their therapeutic efficacy. Recent studies have highlighted nanoemulsion-based botanical formulations as promising therapeutic systems for chronic inflammatory and neuroimmune disorders because they simultaneously target inflammation, oxidative stress, and neuronal dysfunction [20,21]. Gum Arabic is particularly attractive as a natural food-grade emulsifier owing to its excellent emulsifying properties, high biocompatibility, and intrinsic antioxidant and anti-inflammatory activities, which improve formulation stability and protect encapsulated bioactive compounds from physicochemical deterioration [21,22].

The utilization of agro-industrial by-products as sustainable sources of high-value bioactive compounds has received increasing attention because it simultaneously reduces food-processing waste and generates functional ingredients with important health benefits. Our research group has previously demonstrated significant anti-inflammatory activity of wheat germ oil in experimental arthritis [23], antioxidant and anti-inflammatory properties of grape seed and avocado peel extracts [24,25], and beneficial modulation of gut microbiota following administration of an avocado peel-derived bioactive formulation in arthritic rats [25]. In addition, our previous studies have demonstrated that chronic metabolic disorders can impair brain function through gut microbiota dysbiosis, neuroinflammation, oxidative stress, and cholinergic dysfunction, highlighting the importance of neuroimmune interactions in systemic diseases [26]. These findings collectively support the concept that combining grape seed oil, wheat germ oil, and avocado peel oil into a Gum Arabic-stabilized nanoemulsion may integrate their complementary phytochemical profiles while enhancing the bioavailability and therapeutic efficacy of their bioactive constituents.

This study aimed to develop and characterize a novel Gum Arabic-stabilized bioactive oil blend nanoemulsion prepared from grape seed oil, avocado peel oil, and wheat germ oil, and to evaluate its therapeutic efficacy against adjuvant-induced rheumatoid arthritis-associated depression-like behavior in female rats. The underlying mechanisms were investigated through integrated behavioral and biochemical assessments together with molecular docking, network pharmacology, and correlation analyses, with particular emphasis on inflammatory, oxidative stress, cholinergic, and neuroimmune pathways.

## 2. Materials and Methods

### 2.1. Preparation and Characterization of the Oil Nanoemulsions

#### 2.1.1. Preparation of the Oil Blend

Oils were extracted from freeze-dried avocado peel, grape seeds, and wheat germ, as previously described by Mohamed et al. [23,24,25]. Equal proportions of grape seed oil, wheat germ oil, and avocado peel oil (1:1:1, *v*/*v*) were then blended to prepare the oil phase used for nanoemulsion formulation.

#### 2.1.2. Preparation and Lyophilization of the Gum Arabic-Stabilized Oil Blend Nanoemulsion

The Gum Arabic-stabilized oil blend nanoemulsion was prepared using a high-shear homogenization technique with slight modifications to previously reported methods that employ Gum Arabic as a natural emulsifying and stabilizing agent for food-grade nanoemulsions [21]. A Gum Arabic solution (10%, *w*/*v*) was prepared in distilled water and used as the aqueous phase. The oil blend (27 g) was gradually dispersed in 150 mL of the Gum Arabic solution under continuous stirring to obtain a coarse emulsion prior to homogenization, corresponding to an oil-to-aqueous-phase ratio of approximately 1:5.6 (*w*/*v*).

The resulting mixture was homogenized using a high-speed homogenizer (WiseTis HG-15D, Wise Laboratory Instruments, Seoul, Republic of Korea) at 9000 rpm for 10 min at 10 °C until a uniform milky emulsion was formed. The prepared nanoemulsion was stored at 4 °C throughout the experimental period and was used directly for biological evaluation.

For lyophilization, the samples were frozen prior to freeze-drying and subsequently dried using a laboratory freeze-dryer until complete removal of water was achieved. The resulting lyophilized powder was collected and stored in airtight containers at 4 °C until further analysis, including TEM examination and other physicochemical measurements.

Transmission electron microscopy (TEM) analysis. The morphology and droplet size of the nanoemulsion were examined using a JEOL JEM-1230 transmission electron microscope (JEOL Ltd., Tokyo, Japan; RRID:SCR_018036). Representative TEM micrographs were obtained at appropriate magnifications, and droplet diameters were measured directly from the micrographs. The measured droplet diameters in the representative micrographs ranged from approximately 60 to 145 nm, indicating the nanoscale size of the nanoemulsion droplets.

#### 2.1.3. Phytochemical Analysis of the Oil Blend Nanoemulsion

Phenolic compounds were extracted from the prepared oil blend nanoemulsion according to the method of Oliveira Alves et al. [27]; then, total phenolic [28] and total flavonoids [29] were determined. The phenolic profile of the oil blend nanoemulsion was analyzed using HPLC (Agilent 1260 series) under the same chromatographic conditions previously described by Mohamed et al. [30]. Separation was achieved using a Zorbax Eclipse Plus C8 column (4.6 × 250 mm, 5 μm; Agilent Technologies, Santa Clara, CA, USA). Identification and quantification of phenolic compounds were performed by comparison with authentic standards including gallic acid, methyl gallate, chlorogenic acid, catechin, pyrocatechol, rosmarinic acid, syringic acid, rutin, coumaric acid, caffeic acid, naringenin, ferulic acid, vanillin, cinnamic acid, quercetin, hesperetin, and kaempferol. The phytosterols and fatty acid profiles of the oil blend were determined according to AOAC standard procedures [31] using gas–liquid chromatography (GLC). Preparation and identification of fatty acid methyl esters were carried out following the method previously described by Mohamed et al. [24]. In addition, α-tocopherol content was determined by HPLC according to the method of Amaral et al. [32]. 

Analysis was performed using an Agilent 1100 HPLC system equipped with a fluorescence detector and ChemStation software (B.04.03; Agilent Technologies, Santa Clara, CA, USA) using an SI column (150 × 4.6 mm). The excitation and emission wavelengths were adjusted to 290 and 330 nm, respectively. The mobile phase consisted of hexane:isopropanol (99:1, *v*/*v*) delivered at a flow rate of 1 mL/min. Quantification of α-tocopherol was performed by comparing sample peak areas with those of standard solutions. The oxidizability (COX) index of the prepared oil blend nanoemulsion was calculated [25].

### 2.2. Physicochemical Characterization of the Oil Blend Nanoemulsion

The prepared gum Arabic-stabilized oil blend nanoemulsion was characterized for its physical appearance, stability, particle morphology, particle size distribution, surface charge, and physicochemical properties. The freshly prepared nanoemulsion was visually inspected for color, homogeneity, phase separation, creaming, precipitation, and aggregation. The physical stability of the formulation was monitored during refrigerated storage at 4 °C for three weeks over the experimental period.

The pH of the nanoemulsion was measured at room temperature using a calibrated digital pH meter, and the measurements were performed in triplicate [33]. In addition, the oxidative stability of the nanoemulsion was evaluated by determining the peroxide value during the storage period [34].

The morphology and particle size distribution of the nanoemulsion were examined using transmission electron microscopy (TEM). Prior to analysis, the lyophilized nanoemulsion powder was re-dispersed in distilled water and diluted appropriately. A drop of the diluted dispersion was placed onto a carbon-coated copper grid and allowed to air dry at room temperature before imaging. TEM micrographs were obtained at different magnifications to evaluate droplet morphology, dispersion characteristics, and particle size distribution. The particle diameters were estimated directly from representative droplets observed in different microscopic fields [35].

The particle size distribution and average droplet diameter of the nanoemulsion were further determined using dynamic light scattering (DLS) analysis. Before measurement, the nanoemulsion samples were diluted with distilled water to minimize multiple scattering effects. The measurements were performed at room temperature using a particle size analyzer equipped with a laser light scattering system. The average particle size and polydispersity index (PDI) were recorded to assess the droplet size distribution and formulation homogeneity [34].

The zeta potential of the prepared nanoemulsion was also measured using a zeta potential analyzer to evaluate the surface charge and colloidal stability of the emulsion droplets. Diluted nanoemulsion samples were analyzed at room temperature under standard operating conditions, and the resulting zeta potential values were expressed in millivolts (mV) [36].

### 2.3. Biological Experiments

#### 2.3.1. Experimental Animals

Twenty-four adult female Wistar rats weighing 125–171 g were obtained from the Animal House Colony of the National Research Centre (NRC), Cairo, Egypt. Animals were housed individually in stainless-steel cages under standard laboratory conditions at a room temperature of 25 ± 2 °C and relative humidity of approximately 55%, with a 12 h light/12 h dark cycle. Food and water were provided ad libitum throughout the experimental period. Rats were acclimated for one week before the experiment. All animal experiments were approved by the Medical Research Ethics Committee of the National Research Centre, Cairo, Egypt (Approval No. 1342025) and were conducted in accordance with the National Institutes of Health Guide for the Care and Use of Laboratory Animals (NIH Publication No. 85-23, revised 1985). All animal experiments complied with the ARRIVE (Animal Research: Reporting of In Vivo Experiments) guidelines. The study also complied with the World Medical Association (WMA) Statement on Animal Use in Biomedical Research.

#### 2.3.2. Induction of Rheumatoid Arthritis

**Figure 1:**
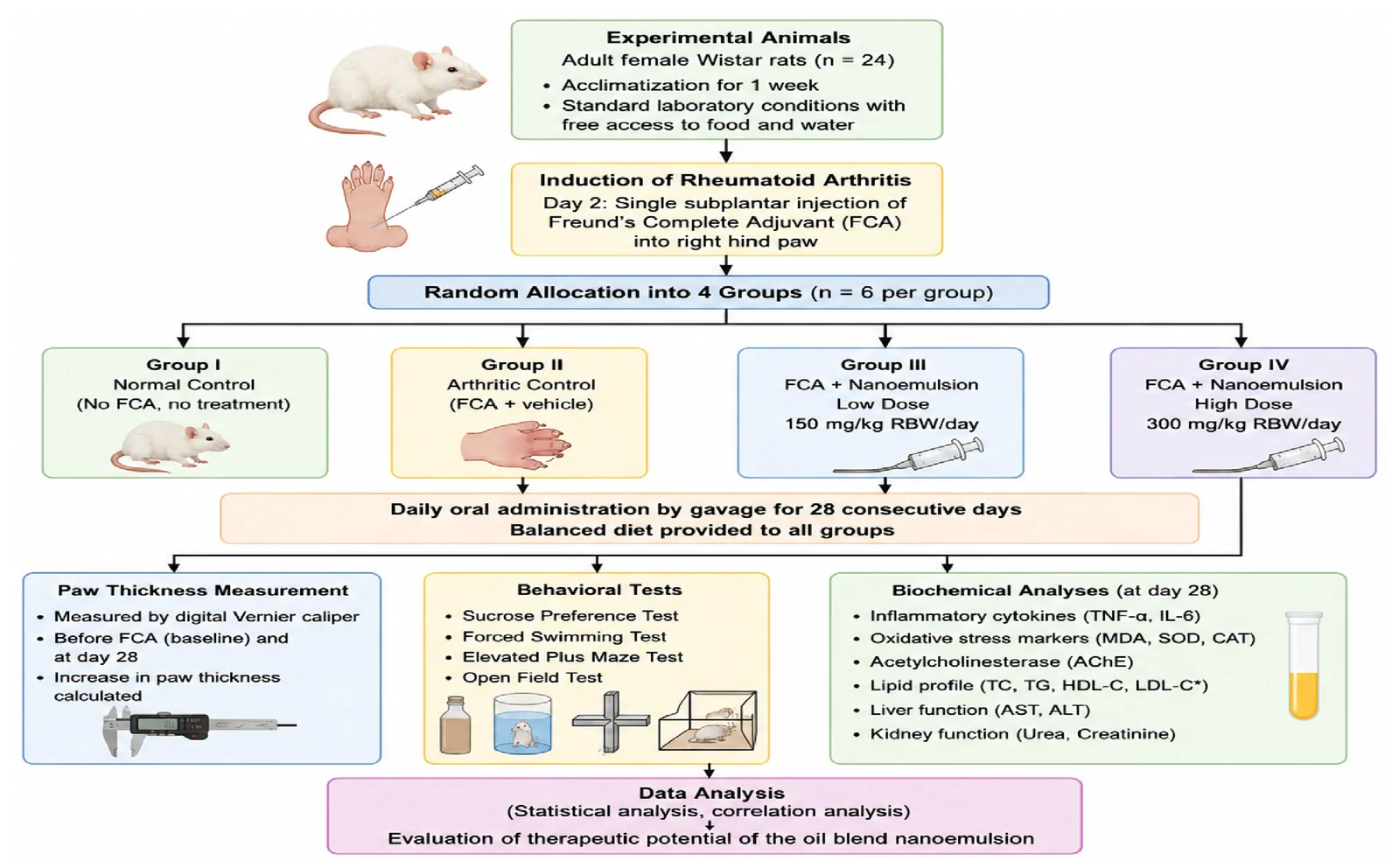
Schematic representation of the experimental workflow used to evaluate the therapeutic efficacy of the lyophilized oil blend nanoemulsion in rats with complete Freund’s adjuvant (CFA)-induced rheumatoid arthritis. * LDL-C was calculated using the equation described in the Section 2 [25].

Arthritis was induced according to the protocol previously described by Mohamed et al. [25]. Briefly, animals received a single subplantar injection of 0.1 mL Freund’s Complete Adjuvant (FCA; F5881, Sigma-Aldrich, St. Louis, MO, USA) into the right hind paw. Each mL of FCA contained 1 mg of heat-killed, dried *Mycobacterium tuberculosis* H37Ra (ATCC 25177), 0.85 mL paraffin oil, and 0.15 mL mannide monooleate. FCA was administered on Day 2 of the experimental protocol. Animals were monitored for the development of local inflammatory signs, particularly paw swelling, following FCA administration. The overall experimental protocol is summarized in Figure 1.

#### 2.3.3. Experimental Design

Following the acclimatization period, the rats were randomly allocated into four experimental groups (*n* = 6 per group):

- Group I: Normal control group.
- Group II: Arthritic control (FCA treated).
- Group III: Arthritic rats were treated orally with the lyophilized oil blend nanoemulsion at 150 mg/kg body weight/day.
- Group IV: Arthritic rats were treated orally with the lyophilized oil blend nanoemulsion at 300 mg/kg body weight/day.

All animals were maintained on a balanced laboratory diet throughout the study. On Day 2, all groups except the normal control received a single FCA injection into the right hind paw. The nanoemulsion-treated groups received the respective doses once daily by oral gavage for 28 consecutive days. Hind paw thickness was measured immediately before FCA injection and at the end of the experimental period using a digital Vernier caliper. The increase in paw thickness was calculated as the difference between the final and initial measurements, and the percentage inhibition of paw inflammation was determined relative to that of the FCA-induced arthritic control group.

#### 2.3.4. Sample Collection

At the end of the experimental period, the rats were fasted overnight and anesthetized before blood collection. Blood samples were centrifuged at 3000 rpm for 15 min to separate plasma, which was stored at −80 °C until biochemical analyses.

#### 2.3.5. Biochemical Analysis

Plasma concentrations of TNF-α (ELISA Kit, Sunlong^®^, Cat. No. SL0722Ra; Hangzhou, China) and IL-6 (ELISA Kit, Sunlong^®^, Cat. No. SL0411Ra) were determined according to the manufacturers’ instructions. Plasma AChE concentrations were quantified using a Rat AChE ELISA Kit (Sunlong^®^, Cat. No. SL0027Ra). According to the manufacturer’s specifications, the AChE assay has a detection range of 0.65–40 ng/mL and a sensitivity of 0.16 ng/mL. For the AChE assay, 10 µL of plasma sample was added to each well with 40 µL of sample diluent, and absorbance was measured at 450 nm. Results were expressed as ng/mL.

Oxidative stress status was evaluated by measuring malondialdehyde (MDA) according to the method of Satoh [37], whereas catalase (CAT) activity was determined according to Aebi [38]. Plasma lipid profile, including total cholesterol (TC), triglycerides (TG), and high-density lipoprotein cholesterol (HDL-C), was determined using commercial colorimetric assay kits according to the manufacturers’ instructions. Low-density lipoprotein cholesterol (LDL-C) was calculated as previously described [25].

Kidney function was assessed by measuring plasma urea and creatinine concentrations, whereas liver function was evaluated by determining aspartate aminotransferase (AST) and alanine aminotransferase (ALT) activities using commercial colorimetric assay kits according to the manufacturers‘ instructions.

#### 2.3.6. Behavioral Assessment

Behavioral tests were performed during the light phase in a quiet room under controlled environmental conditions. Animals were acclimated to the testing room for 30 min before testing, and the apparatuses were cleaned with 70% ethanol between animals to eliminate olfactory cues.

##### Sucrose Preference Test (SPT)

The sucrose preference test was conducted to evaluate anhedonia-like behavior, following the method described by Berke et al. [39]. Rats were provided with two pre-weighed bottles containing 2% sucrose solution and tap water for 48 h on days 0–2 and 13–15 post-induction. Bottle positions were switched after 24 h to minimize side preference bias. Sucrose preference was calculated as:

Sucrose preference (%) = [Sucrose intake/(Sucrose intake + Water intake)] × 100

A reduction in sucrose preference was considered indicative of depression-like behavior.

##### Forced Swim Test (FST)

The forced swim test was performed according to the method of Porsolt et al. [40]. Rats were individually placed in a transparent cylinder (50 cm height × 20 cm diameter) containing water (25 ± 1 °C, depth 30 cm) for 6 min. Immobility time during the last 4 min was recorded and used as an index of behavioral despair.

##### Elevated Plus Maze (EPM)

Anxiety-like behavior was evaluated using an elevated plus maze, which consisted of two open arms and two closed arms elevated 50 cm above the floor [41]. Rats were placed individually in the center of the maze and allowed to explore for 5 min. The time spent in the open arms and the number of open-arm entries were recorded.

##### Open Field Test (OFT)

Locomotor and exploratory activities were assessed using the open field test [42]. Rats were individually placed in the center of a square arena and allowed to explore for 5 min. The number of lateral ambulations, central ambulations, and rearing behaviors was recorded.

### 2.4. Molecular Docking Studies of the Bioactive Oil Blend Nanoemulsion

The three-dimensional crystal structures of TNF-α, IL-6, and AChE were retrieved from the RCSB Protein Data Bank under PDB ID: 2AZ5, 1ALU, 7E3H [43,44,45], respectively. Protein preparation was performed using AutoDock Tools version 1.5.7 [46], which included removal of water molecules, addition of polar hydrogen atoms, and assignment of Kollman partial charges. The binding sites were defined using the co-crystallized ligand present in each protein. The prepared protein structures were saved in PDBQT format. The structures of the phenolic compounds (catechin and gallic acid) and volatile compounds (alpha-tocopherol, β-sitosterol, campasterol, stigmasterol) identified in the oil blend nanoemulsion were retrieved from the PubChem database. Ligand structures were energy minimized using Avogadro software (version 1.2.0) employing the MMFF94 force field. Molecular docking simulations were conducted using AutoDock Vina version 1.5.7 [46], and the docking grid boxes were centered on the validated ligand-binding pockets of each protein as defined by the co-crystallized structure. Protein–ligand interactions were visualized and analyzed using BIOVIA Discovery Studio Visualizer (version 2020).

### 2.5. Network Pharmacology Analysis of the Bioactive Oil Blend Nanoemulsion

Based on the HPLC and GC–MS characterization of the Gum Arabic-stabilized bioactive oil blend nanoemulsion, seven representative bioactive compounds, namely β-sitosterol, campesterol, stigmasterol, α-tocopherol, catechin, gallic acid, and quercetin, were selected for network pharmacology analysis. These compounds were chosen because they represent the major phytosterols, phenolic compounds, and tocopherol identified in the nanoemulsion and are recognized as key contributors to its biological activity. The canonical SMILES structures of these compounds were retrieved from the PubChem database and submitted to the SwissTargetPrediction database (http://www.swisstargetprediction.ch, accessed on 1 July 2026), with the species restricted to *Homo sapiens*, to predict their potential protein targets. The predicted targets for all seven compounds were merged, and duplicate entries were removed to generate a non-redundant target dataset.

To identify disease-related targets, rheumatoid arthritis- and depression-associated genes were retrieved from the GeneCards database (https://www.genecards.org, accessed on 1 July 2026) using the keywords “Rheumatoid arthritis” and “Depression”, respectively. The retrieved genes were ranked according to their GeneCards relevance scores, and the top 100 genes for each disease were selected for subsequent analyses. The predicted compound targets were then intersected with the rheumatoid arthritis and depression gene datasets to identify common therapeutic targets potentially involved in the biological effects of the nanoemulsion.

A compound–target interaction network and a compound–target–disease network were constructed using Cytoscape version 3.10.3 to visualize the relationships among the seven bioactive compounds, the predicted targets, the common therapeutic targets, rheumatoid arthritis, and depression. Network topology analysis was performed to identify the principal bioactive compounds and key molecular targets potentially responsible for the anti-rheumatoid arthritis and antidepressant activities of the Gum Arabic-stabilized bioactive oil blend nanoemulsion.

The common therapeutic targets were further analyzed using the STRING database (version 12.0; https://string-db.org, accessed on 1 July 2026) to explore protein–protein interactions. The resulting network, together with the molecular docking results and relevant published literature, was used to construct an integrated mechanistic model illustrating the potential multi-component–multi-target therapeutic mechanisms of the nanoemulsion.

### 2.6. Statistical Analysis

Statistical analyses were performed using GraphPad Prism version 8. Data are presented as mean ± SE. Data normality was tested with the Shapiro-Wilk test. One-way ANOVA followed by Tukey’s multiple comparison test was used for the normally distributed data. The non-normally distributed data were analyzed using the Kruskal-Wallis test followed by Dunn’s multiple comparison test. Two-way repeated-measures ANOVA followed by Sidak’s multiple comparison test was used for the sucrose preference test. Correlation between behavioral and biochemical parameters was evaluated using Spearman’s rank correlation coefficient, and the results were visualized using a correlation heatmap and network analysis using R software version 5.4.1. Statistical significance was considered at *p* < 0.05.

## 3. Results

### 3.1. Chemical Composition of the Oil Blend Nanoemulsion

The prepared oil blend nanoemulsion (Table 1) exhibited a high total phenolic content (320 ± 2.54 mg GAE/100 g) and a high total flavonoid content (145 ± 1.64 mg QE/100 g), indicating its richness in bioactive antioxidant constituents. HPLC analysis identified catechin as the predominant phenolic compound (600 ± 0.07 µg/g), followed by gallic acid (270 ± 0.03 µg/g) and chlorogenic acid (210 ± 0.03 µg/g). Among the flavonoids, quercetin (170 ± 0.07 µg/g), rutin (155 ± 0.03 µg/g), and kaempferol (90 ± 0.06 µg/g) were the major constituents. Moderate amounts of ferulic acid (140 ± 0.08 µg/g), caffeic acid (80 ± 0.05 µg/g), and p-coumaric acid (65 ± 0.04 µg/g) were also detected, whereas cinnamic acid, vanillin, hesperetin, and naringenin were present at comparatively lower concentrations.

The fatty acid analysis of the prepared nanoemulsion (Table 2) revealed a predominance of unsaturated fatty acids, which accounted for 81.5 ± 0.086% of the total identified fatty acids, whereas saturated fatty acids represented only 15.04 ± 0.055%. Oleic acid was the major fatty acid component (44.5 ± 0.252%), followed by linoleic acid (32.9 ± 0.551%) and linolenic acid (4.1 ± 0.066%), which indicates that the nanoemulsion is rich in mono- and polyunsaturated fatty acids, including ω − 6 and ω − 3 fatty acids.

The phytosterol analysis (Table 2) also demonstrated the presence of considerable levels of bioactive phytosterols, with β-sitosterol representing the predominant phytosterol fraction (46.6 ± 0.224%), followed by campesterol and stigmasterol. The total phytosterol content reached 70.8 ± 0.214%, reflecting the richness of the nanoemulsion in phytosterol compounds. The oil blend shows a high content of α-tocopherol (650 ± 4.72 mg/100 g).

The calculated oxidizability index (COX) of the oil blend nanoemulsion was 4.72 (Table 2), indicating moderate oxidative susceptibility based on its fatty acid composition. This value reflects the predominance of unsaturated fatty acids, particularly oleic and linoleic acids, whereas the high levels of natural antioxidants, including tocopherols and phenolic compounds, together with nanoemulsion encapsulation, are expected to enhance the oxidative stability of the formulation.

### 3.2. Visual Appearance and Physical Stability

The prepared nanoemulsion remained physically stable for three weeks at 4 °C throughout the experimental period, with no visible phase separation, creaming, or precipitation.

### 3.3. pH and Peroxide Values of the Oil Blend Nanoemulsion

The pH of the prepared nanoemulsion (Table 3) remained relatively stable during refrigerated storage, showing only a slight increase from 6.03 initially to 6.18 after three weeks, indicating good physicochemical stability of the emulsion system. Intermediate values during the first and second weeks did not differ significantly from either the initial or the third week values.

Similarly, the peroxide value of the nanoemulsion gradually increased from 2.35 to 2.76 meq O_2_/kg oil during the storage period. A significant increase in peroxide value was observed by the third week of storage. Nevertheless, the values obtained remained relatively low throughout the experimental period, indicating limited lipid oxidation and good oxidative stability of the gum Arabic-stabilized nanoemulsion. The slight increase in peroxide value may be attributed to the natural oxidation of unsaturated fatty acids, whereas the relatively low oxidation levels suggest a protective effect of gum Arabic together with the naturally occurring antioxidant compounds present in the oil blend.

Overall, the prepared nanoemulsion maintained acceptable pH and oxidative stability under refrigerated storage conditions, supporting its suitability for further biological and functional applications.

### 3.4. Morphological Characterization, Dynamic Light Scattering (DLS) and Zeta Potential Analysis

**Figure 2:**
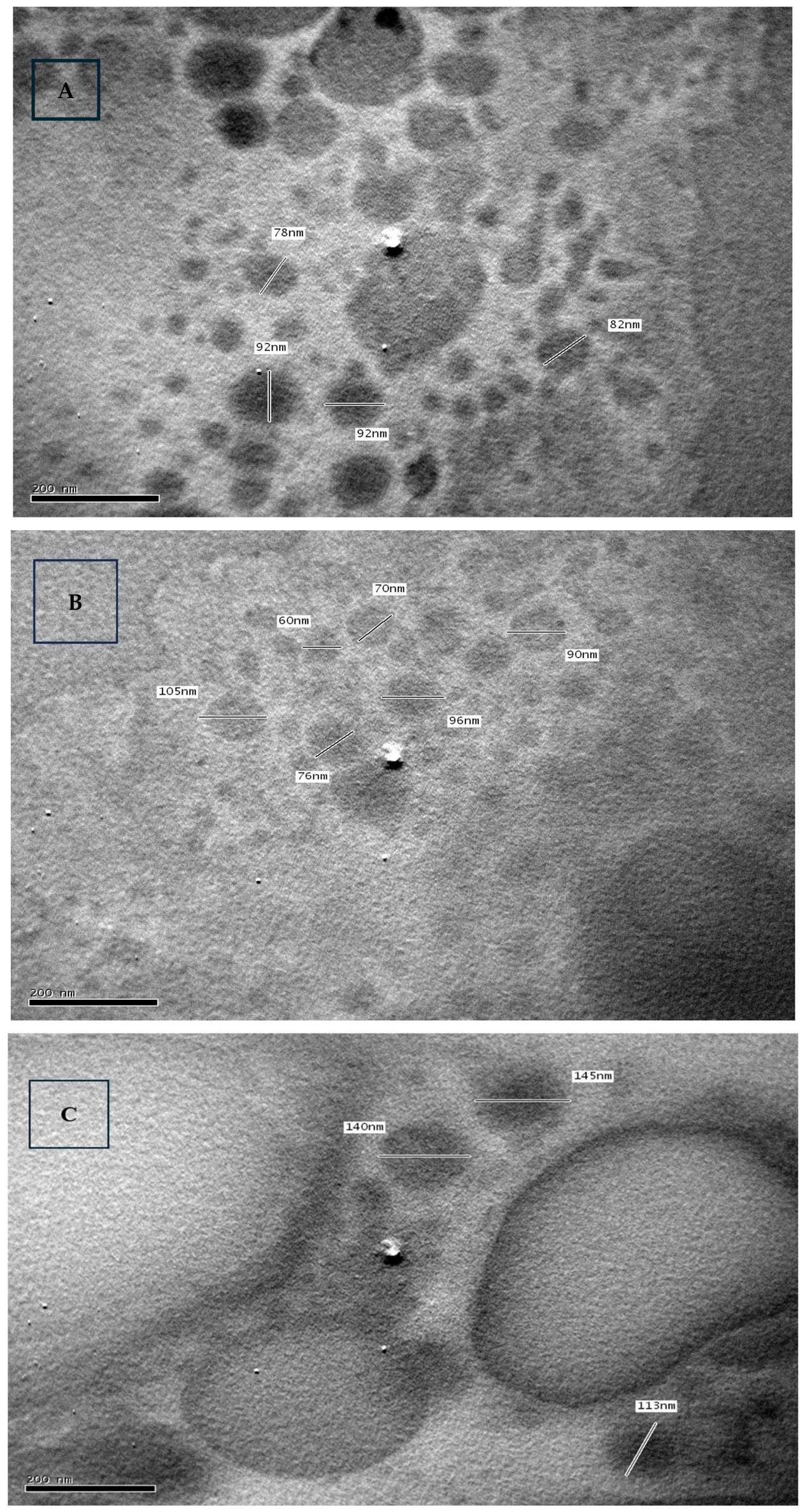
Transmission electron microscopy (TEM) micrographs of the lyophilized Gum Arabic-stabilized oil blend nanoemulsion at different magnifications. The images revealed predominantly spherical and well-dispersed droplets with measured diameters ranging from approximately 60 to 145 nm, indicating the formation of a nanoscale emulsion system. Panel (**A**) shows the overall distribution of nano-sized droplets, whereas panels (**B**,**C**) show representative droplet morphology and size measurements. Limited aggregation was observed in the representative TEM fields.

The physicochemical characterization of the prepared Gum Arabic-stabilized oil blend nanoemulsion confirmed the successful formation of a relatively stable oil-in-water nanoemulsion system with nanoscale characteristics. Transmission electron microscopy (TEM) micrographs (Figure 2A–C) revealed predominantly spherical to nearly spherical droplets, with particle sizes mainly ranging from approximately 60 to 145 nm. Figure 2A illustrates the overall dispersion pattern of the nano-sized droplets, while Figure 2B,C present representative particle size measurements and droplet morphology at higher magnifications. The droplets appeared relatively well dispersed and uniformly distributed throughout the examined fields, with only minimal aggregation observed.

The obtained TEM findings suggest that Gum Arabic effectively stabilized the dispersed oil droplets during both the emulsification and lyophilization processes. In addition, the nanoemulsion maintained its structural integrity after freeze-drying and reconstitution without obvious collapse or severe deformation of the droplets. Although some variation in droplet size was observed, most particles remained within the nanometric range, indicating the successful preparation of a moderately polydisperse nanoemulsion system.

**Figure 3:**
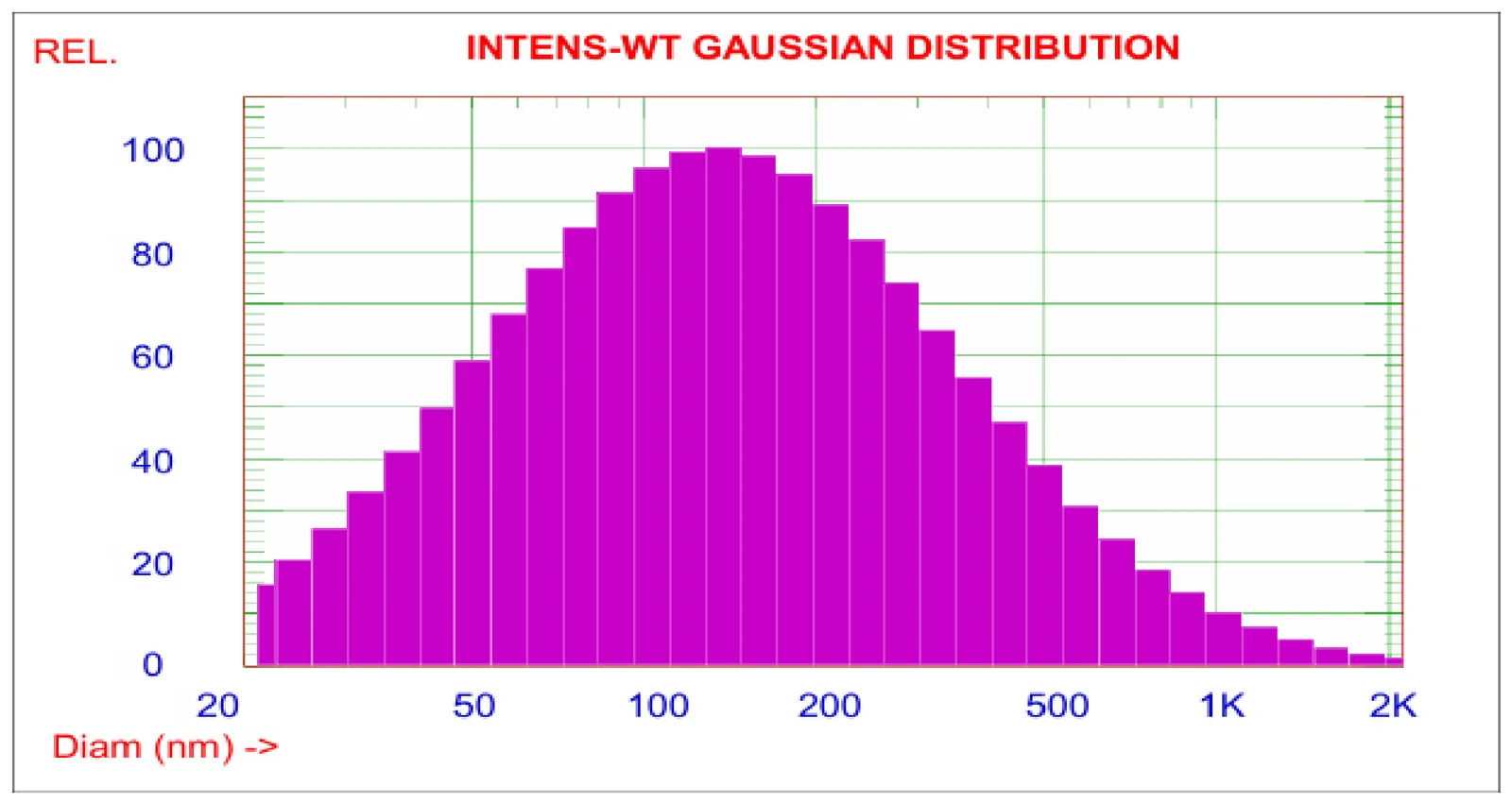
Intensity-weighted Gaussian particle size distribution of the prepared Gum Arabic-stabilized oil blend nanoemulsion determined by dynamic light scattering (DLS).

Dynamic light scattering (DLS) analysis further supported the TEM observations. The prepared nanoemulsion exhibited a mean droplet diameter of approximately 211.9 nm with a polydispersity index (PDI) of 0.914 (Figure 3). The particle size distribution analysis showed that 50% of the droplets were smaller than 139.9 nm, while 75% were below 261.7 nm, indicating that the majority of droplets were distributed within the nanometric range despite the presence of some larger particles contributing to the broader size distribution profile. The relatively high PDI value reflects moderate polydispersity, which is consistent with the variability in droplet sizes observed in the TEM micrographs. This broad distribution may be related to the natural composition of the oil blend and the use of Gum Arabic as a biopolymeric stabilizer.

**Figure 4:**
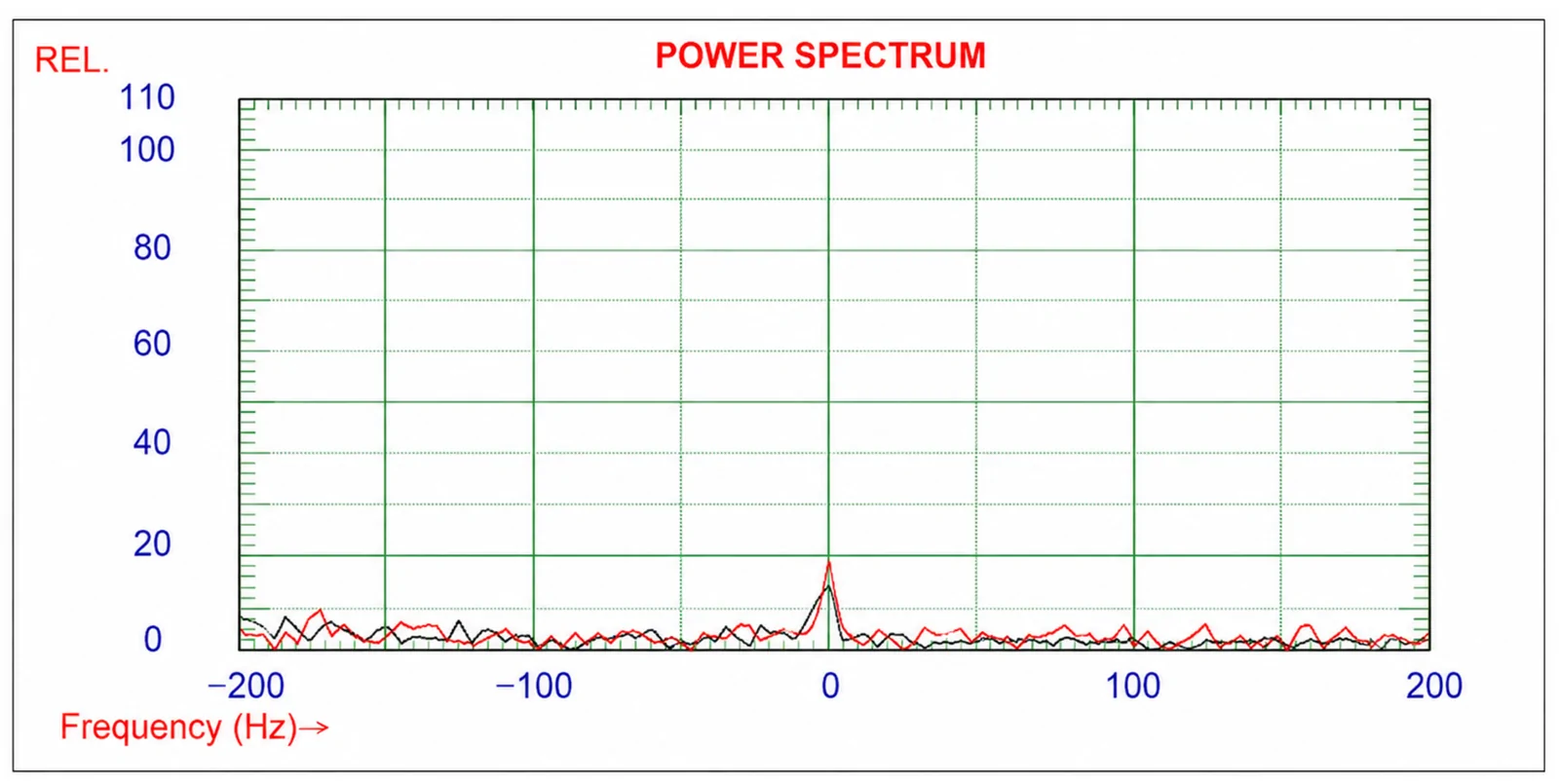
Zeta potential spectrum of the prepared Gum Arabic-stabilized oil blend nanoemulsion shows a negative surface charge, which indicates moderate electrostatic stability of the nanoemulsion droplets. The red line represents the nanoemulsion sample, whereas the black line represents the background/reference. REL.: relative power; Frequency (Hz): frequency in hertz.

The zeta potential analysis (Figure 4) revealed an average zeta potential value of −14.88 mV, indicating the presence of negatively charged droplets on the nanoemulsion surface. The negative surface charge may be attributed to the ionizable functional groups present in Gum Arabic, which likely contributed to the electrostatic stabilization of the dispersed droplets. Although the obtained zeta potential value indicates moderate stability, the combination of electrostatic repulsion and steric stabilization provided by Gum Arabic appears sufficient to minimize droplet aggregation and maintain acceptable dispersion stability throughout the storage period.

Collectively, the TEM, DLS, and zeta potential findings confirm the successful preparation of a relatively stable Gum Arabic-stabilized oil blend nanoemulsion with acceptable nanoscale characteristics, good dispersion properties, and limited aggregation, supporting its suitability for subsequent biological and therapeutic applications.

### 3.5. Effect of the Oil Blend Nanoemulsion on Paw Inflammation

**Figure 5:**
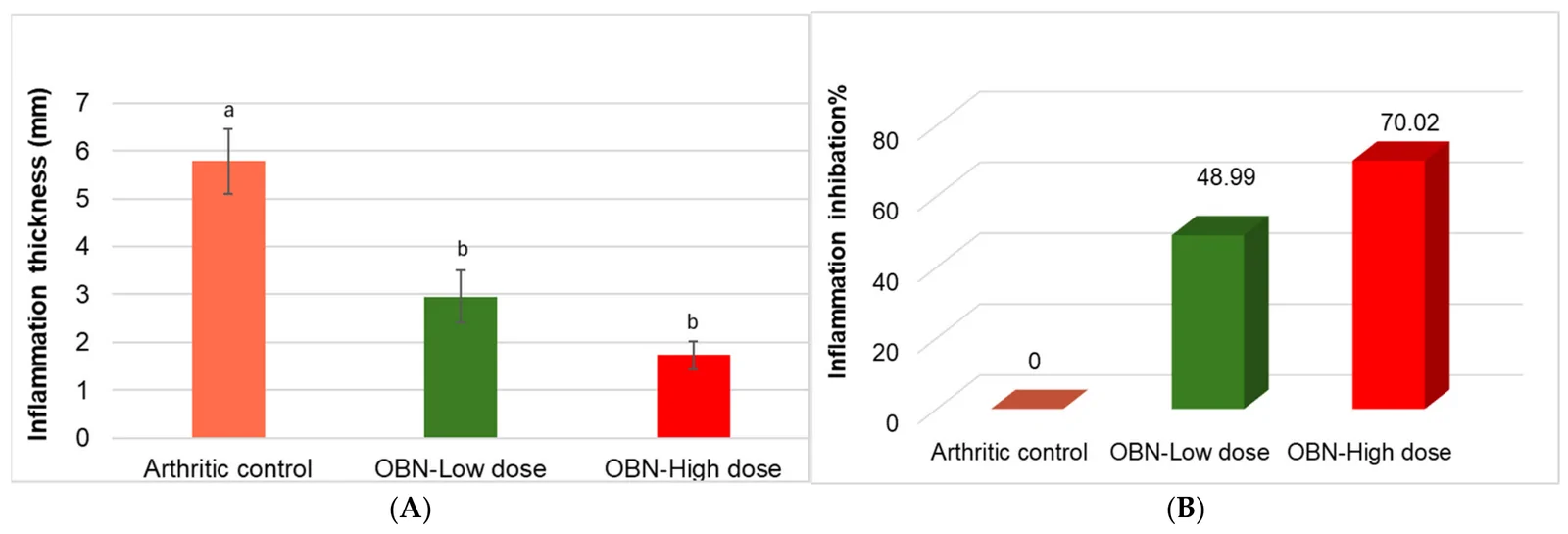
Effect of the oil blend nanoemulsion (OBN) on paw inflammation in adjuvant-induced arthritic rats. (**A**) Paw thickness (mm). (**B**) Percentage inhibition of paw inflammation relative to the arthritic control group. Data are presented as mean ± SE (*n* = 6). Statistical significance was assessed using one-way ANOVA followed by Tukey’s multiple-comparison post hoc test across all groups. Groups with different superscript letters indicate significant differences (*p* ≤ 0.05), whereas groups that share at least one letter are not significantly different (*p* > 0.05).

Adjuvant-induced arthritis markedly increased paw thickness compared with the normal control group (Figure 5A). Treatment with the oil blend nanoemulsion significantly attenuated paw inflammation in a dose-dependent manner. The low-dose nanoemulsion reduced paw thickness to 2.95 ± 0.55 mm, corresponding to 48.99% inhibition of paw inflammation, whereas the high-dose nanoemulsion produced a greater reduction to 1.73 ± 0.29 mm, representing 70.02% inhibition compared with the arthritic control group (Figure 5B).

The arthritic control group exhibited significant biochemical alterations compared with the normal control group (Table 4), including marked elevation in AChE, IL-6, TNF-α, and MDA levels, together with a significant reduction in CAT activity, indicating a severe inflammatory and oxidative stress status associated with adjuvant-induced arthritis. Treatment with the prepared oil blend nanoemulsion significantly ameliorated these alterations in a dose-dependent manner. Both low- and high-dose nanoemulsion treatments reduced the elevated levels of inflammatory cytokines (IL-6 and TNF-α) and the lipid peroxidation marker (MDA) compared with the untreated arthritic group. The high-dose nanoemulsion showed more pronounced improvement, with values approaching those of the normal control group. Similarly, CAT activity was significantly restored in the treated groups compared with the arthritic control group, indicating an improvement in antioxidant defense mechanisms. In addition, treatment with the nanoemulsion normalized the elevated AChE levels observed in arthritic rats, particularly in the high-dose group, suggesting a possible protective effect against arthritis-associated neurochemical alterations. Overall, these findings indicate that the prepared oil blend nanoemulsion exerts considerable antioxidant and anti-inflammatory effects in arthritic rats and may contribute to attenuation of arthritis-associated oxidative stress and neurobehavioral disturbances.

As shown in Table 5, adjuvant-induced rheumatoid arthritis produced significant metabolic, hepatic, and renal disturbances compared with the normal control group. Arthritic rats exhibited significantly elevated plasma total cholesterol (T-Ch), triglycerides (TG), and low-density lipoprotein cholesterol (LDL-Ch), accompanied by a significant reduction in high-density lipoprotein cholesterol (HDL-Ch). Liver function was also impaired, as evidenced by significant increases in aspartate aminotransferase (AST) and alanine aminotransferase (ALT) activities. In addition, plasma urea and creatinine concentrations were significantly elevated, indicating renal dysfunction. Oral administration of the oil blend nanoemulsion at both doses effectively ameliorated these alterations. Treatment restored the lipid profile to values comparable with those of the normal control group by significantly reducing T-Ch, TG, and LDL-Ch while increasing HDL-Ch (*p* < 0.05). Similarly, both doses normalized AST, ALT, and creatinine levels, while plasma urea was markedly reduced, with the high-dose nanoemulsion showing the greatest improvement. These findings demonstrate that the prepared oil blend nanoemulsion effectively protected against rheumatoid arthritis-induced metabolic disturbances and hepatic and renal dysfunction.

No significant differences were observed in the initial body weights (Table 6) among the experimental groups, indicating comparable baseline conditions before arthritis induction and treatment administration. However, the arthritic group showed significant reductions in final body weight and body weight gain compared with the normal control group, reflecting the adverse metabolic and inflammatory effects associated with adjuvant-induced arthritis.

Treatment with the prepared oil blend nanoemulsion improved body weight gain (Table 6) in both treated groups compared to the untreated arthritic group. The low- and high-dose nanoemulsion groups exhibited body weight gain values approaching those of the normal control group, suggesting partial restoration of normal growth and nutritional status.

In addition, the arthritic group exhibited a significant increase in relative liver weight compared with the normal control group, which may indicate inflammatory or metabolic alterations associated with arthritis. Administration of the oil blend nanoemulsion reduced the relative liver weight compared to the arthritic group, particularly in the high-dose group, indicating a possible protective effect against arthritis-associated hepatic alterations.

Overall, these findings suggest that the prepared nanoemulsion may improve the nutritional and physiological status of arthritic rats and may attenuate arthritis-associated metabolic disturbances.

### 3.6. Effect of the Oil Blend Nanoemulsion on Behavioral Performance in Adjuvant-Induced Arthritic Rats

**Figure 6:**
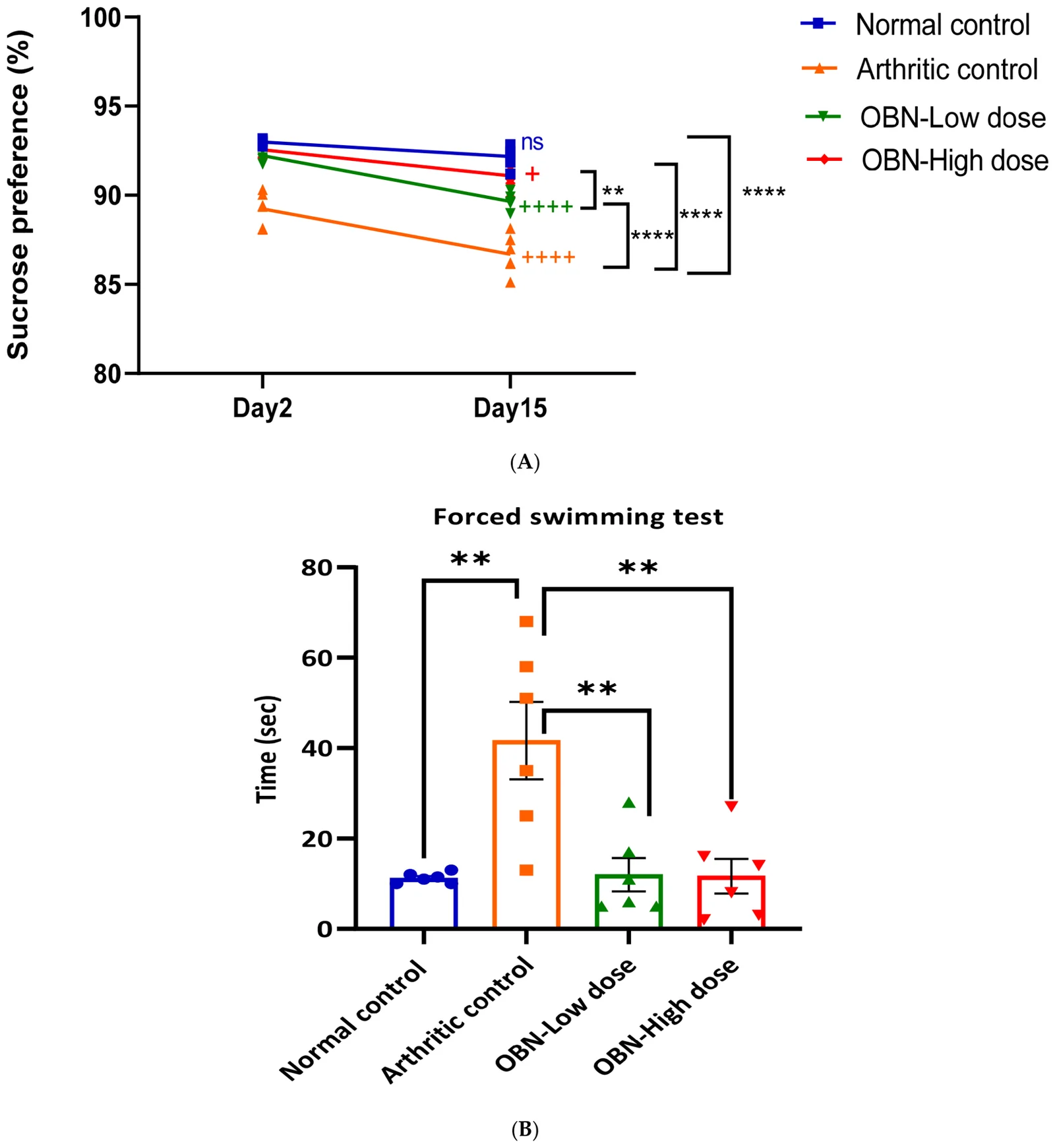

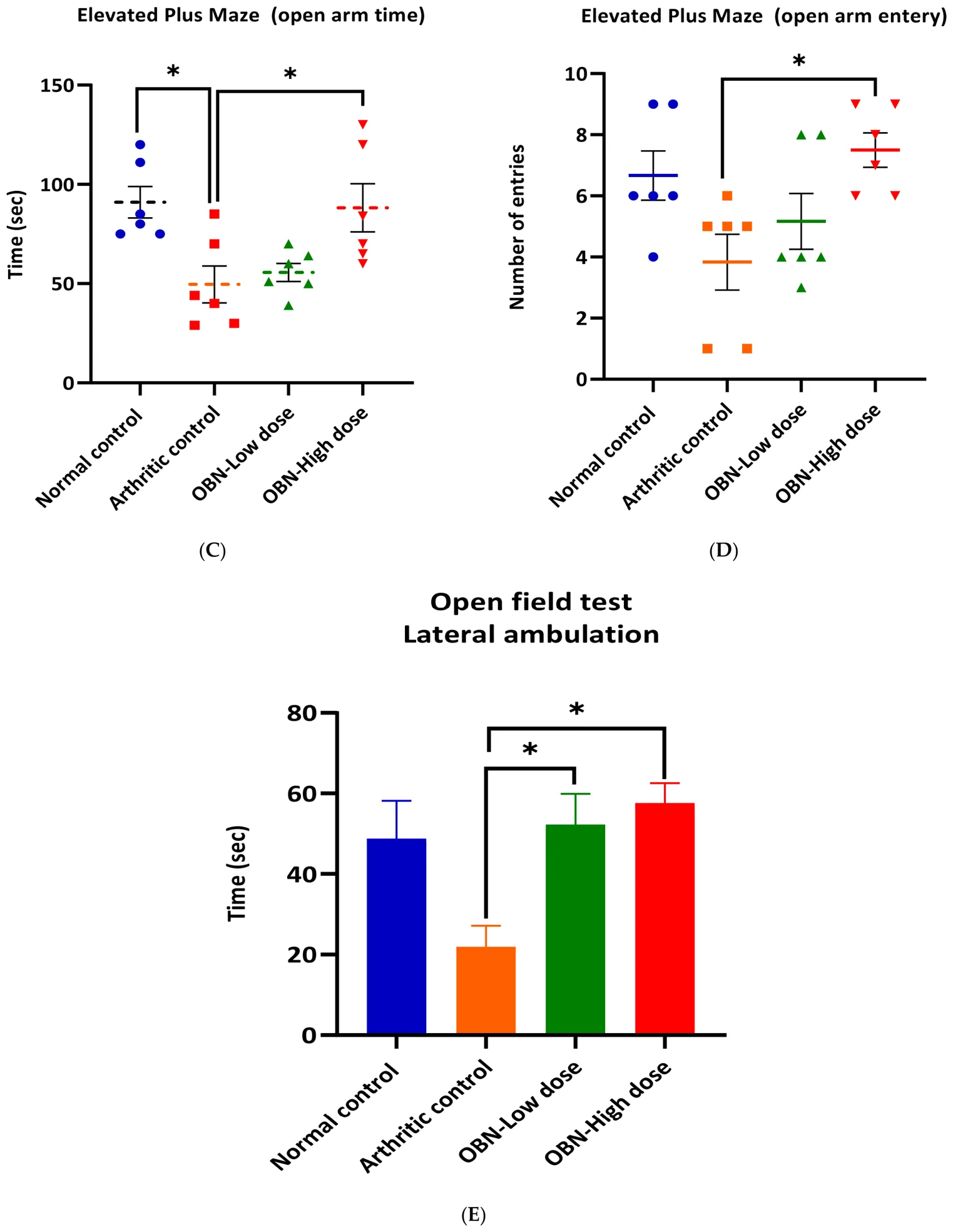

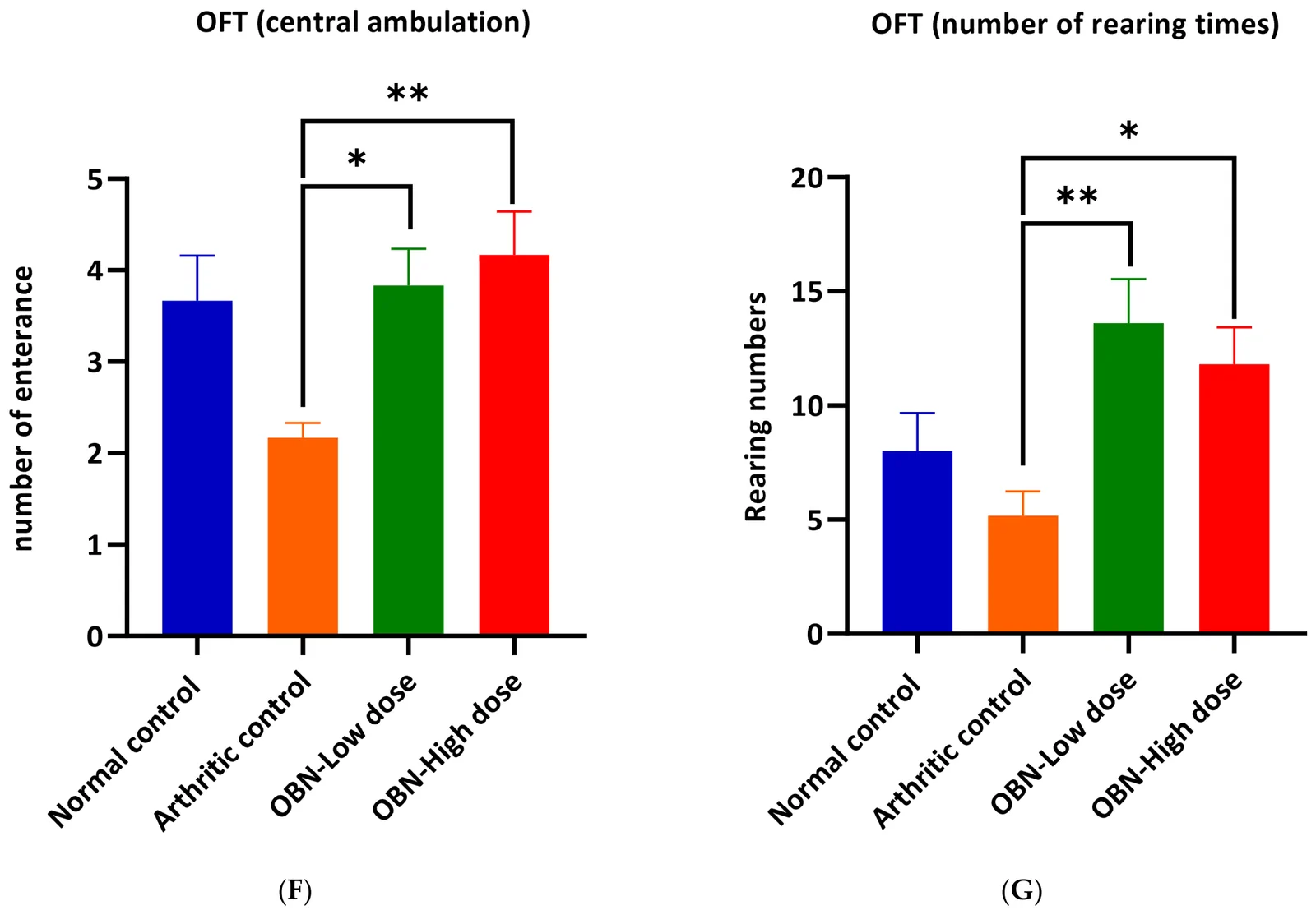
(**A–D**) Effects of the oil blend nanoemulsion on depression- and anxiety-like behaviors in Complete Freund’s Adjuvant-induced arthritic rats. (**A**) Sucrose preference was evaluated on Days 2 and 15 after arthritis induction. Values are expressed as mean ± SE (*n* = 6). Data were analyzed using two-way repeated-measures ANOVA followed by Sidak’s multiple comparisons test. ** *p* ≤ 0.01 and **** *p* ≤ 0.0001 indicate significant differences among experimental groups on Day 15. ns indicates no significant difference, whereas + *p* ≤ 0.05 and ++++ *p* ≤ 0.0001 indicate significant differences between Day 2 and Day 15 within the same experimental group. (**B**) Immobility time in the forced swimming test. Data are expressed as mean ± SE (*n* = 6) and were analyzed using one-way ANOVA followed by Tukey’s multiple comparisons test. ** *p* < 0.01 was considered statistically significant. (**C**) Time spent in the open arms (s) and (**D**) number of open-arm entries in the elevated plus maze. Values are expressed as mean ± SE (*n* = 6). Data for open-arm time were analyzed using one-way ANOVA followed by Tukey’s multiple comparisons test, whereas open-arm entries were analyzed using the Kruskal–Wallis test followed by Dunn’s multiple comparisons test. * *p* < 0.05 was considered statistically significant. OBN-Low dose: oil blend nanoemulsion low dose; OBN-High dose: oil blend nanoemulsion high dose. (**E**–**G**) Effect of the oil blend nanoemulsion on open field performance in Complete Freund’s Adjuvant-induced arthritic rats. (**E**) Lateral ambulation (number of squares crossed). (**F**) Central ambulation (number of squares crossed). (**G**) Rearing frequency (number of rearings). Values are expressed as mean ± SE (*n* = 6). Data for lateral ambulation and rearing frequency were analyzed using one-way ANOVA followed by Tukey’s multiple comparisons test, whereas central ambulation was analyzed using the Kruskal–Wallis test followed by Dunn’s multiple comparisons test. * *p* < 0.05 and ** *p* < 0.01 were considered statistically significant. OBN-Low dose: oil blend nanoemulsion low dose; OBN-High dose: oil blend nanoemulsion high dose.

The behavioral assessment demonstrated that adjuvant-induced arthritis significantly impaired both depression- and anxiety-related behaviors compared to the normal control group (Figure 6A–G).

#### 3.6.1. Effect of the Oil Blend Nanoemulsion on Sucrose Preference

The sucrose preference test demonstrated that adjuvant-induced arthritis significantly reduced sucrose preference compared with the normal control group at both Day 2 and Day 15 (Figure 6A), indicating the development of anhedonia-like behavior. The reduction was more pronounced in the arthritic control group on Day 15 than on Day 2 (*p* < 0.05). Treatment with the oil blend nanoemulsion significantly improved sucrose preference in a dose-dependent manner. The low-dose nanoemulsion partially restored sucrose preference, whereas the high-dose nanoemulsion increased sucrose preference to a level that was not significantly different from that of the normal control group on Day 15. No significant changes were observed between Day 2 and Day 15 in the normal control group, while both nanoemulsion-treated groups showed progressive improvement during the treatment period, with the high-dose group exhibiting the greatest recovery.

#### 3.6.2. Forced Swim Test

As shown in Figure 6B, arthritic rats exhibited a significant increase in immobility time compared with the normal control group (*p* < 0.01), indicating enhanced depression-like behavior. Treatment with the oil blend nanoemulsion significantly reduced immobility time in both the low- and high-dose groups compared to the untreated arthritic group (*p* < 0.01). No significant differences were observed between the nanoemulsion-treated groups and the normal control group, indicating that treatment effectively restored behavioral performance toward normal levels.

#### 3.6.3. Elevated Plus Maze Test

As shown in Figure 6C, arthritic rats spent significantly less time in the open arms of the elevated plus maze than the normal control group (*p* < 0.05), indicating impaired emotional behavior associated with adjuvant-induced arthritis. Treatment with the oil blend nanoemulsion increased the time spent in the open arms in a dose-dependent manner. The high-dose nanoemulsion restored open-arm exploration to a level comparable to that of the normal control group, whereas the low-dose treatment showed partial improvement.

The number of open-arm entries exhibited a similar trend (Figure 6D), with the high-dose group showing the highest number of entries and the arthritic group showing the lowest. However, these differences were not statistically significant among the experimental groups (*p* > 0.05).

#### 3.6.4. Open Field Test

The open field test further demonstrated behavioral impairments in arthritic rats (Figure 6E–G). Compared with the normal control group, arthritic rats exhibited significantly lower lateral ambulation and central ambulation (*p* < 0.05), indicating reduced spontaneous locomotor and exploratory activities. Administration of the oil blend nanoemulsion significantly improved both parameters. The low- and high-dose nanoemulsion groups showed lateral and central ambulation values comparable to those of the normal control group, with no significant differences among these groups.

Similarly, rearing frequency was significantly reduced in the arthritic control group compared with the normal control group (*p* < 0.05). Treatment with the oil blend nanoemulsion significantly increased rearing behavior; the low-dose group exhibited the highest exploratory activity, followed by the high-dose group. These findings indicate that the nanoemulsion effectively restored locomotor and exploratory performance impaired by adjuvant-induced arthritis.

Overall, the behavioral findings demonstrated that the oil blend nanoemulsion effectively ameliorated rheumatoid arthritis-associated behavioral impairments, as evidenced by improvements in depression-like behavior, as well as eth restoration of locomotor activity, exploratory behavior, and overall emotional performance.

### 3.7. Correlation Analysis Between Behavioral and Biochemical Parameters

**Figure 7:**
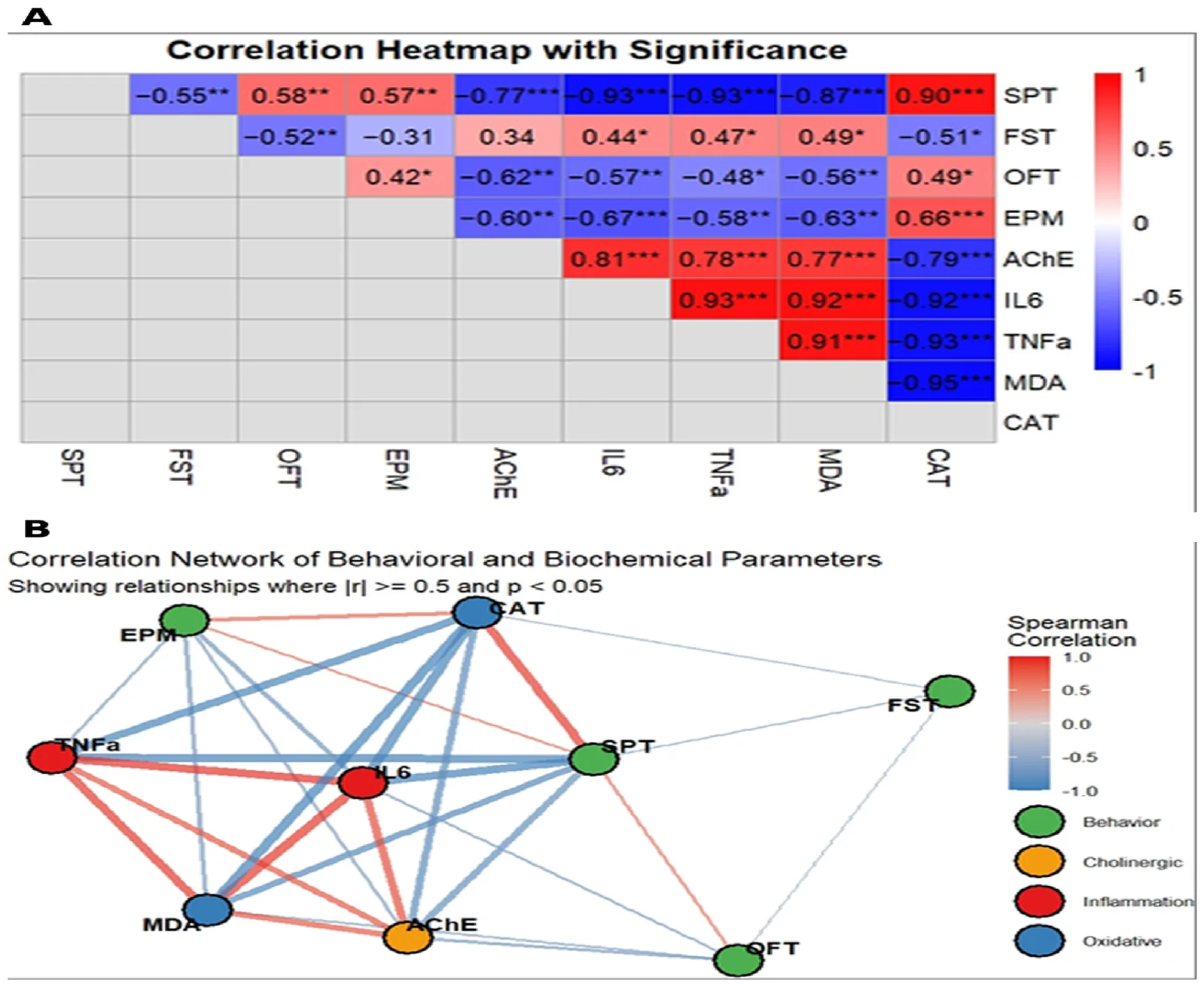
Correlation analysis between behavioral performance and biochemical biomarkers in the arthritis-associated depression model. (**A**) Spearman’s correlation heatmap showing the relationships among behavioral parameters and cholinergic, inflammatory, and oxidative stress biomarkers. (**B**) Spearman’s correlation network illustrating significant correlations (|r| ≥ 0.50, *p* < 0.05). Color intensity represents the magnitude of Spearman’s correlation coefficient, ranging from red (positive correlation) to blue (negative correlation). Node colors indicate behavioral parameters (green), cholinergic biomarkers (orange), inflammatory biomarkers (red), and oxidative stress biomarkers (blue). Edge thickness is proportional to the strength of the correlation coefficient. * *p* < 0.05, ** *p* < 0.01, *** *p* < 0.001.

Spearman’s correlation analysis revealed significant associations between behavioral performance and biochemical biomarkers in the arthritis-associated depression model (Figure 7A,B). Sucrose preference exhibited strong negative correlations with acetylcholinesterase (AChE; r = −0.77, *p* < 0.01), IL-6 (r = −0.93, *p* < 0.001), TNF-α (r = −0.93, *p* < 0.001), and MDA (r = −0.87, *p* < 0.001), whereas it showed a strong positive correlation with catalase (CAT; r = 0.90, *p* < 0.001). Forced swimming time was positively correlated with IL-6 (r = 0.44, *p* < 0.05), TNF-α (r = 0.47, *p* < 0.05), and MDA (r = 0.49, *p* < 0.05), while it exhibited a negative correlation with CAT (r = −0.51, *p* < 0.05).

Similarly, open field performance and elevated plus maze behavior demonstrated significant negative correlations with inflammatory and oxidative stress markers. Open field test scores were negatively correlated with AChE (r = −0.62, *p* < 0.01), IL-6 (r = −0.57, *p* < 0.01), TNF-α (r = −0.48, *p* < 0.05), and MDA (r = −0.56, *p* < 0.01), and were positively correlated with CAT (r = 0.49, *p* < 0.05). Elevated plus maze performance also showed significant negative correlations with AChE (r = −0.60, *p* < 0.01), IL-6 (r = −0.67, *p* < 0.01), TNF-α (r = −0.58, *p* < 0.01), and MDA (r = −0.63, *p* < 0.01), together with a positive correlation with CAT (r = 0.66, *p* < 0.001).

Among the biochemical parameters, AChE showed strong positive correlations with IL-6 (r = 0.81, *p* < 0.001), TNF-α (r = 0.78, *p* < 0.001), and MDA (r = 0.77, *p* < 0.001), whereas CAT was negatively correlated with AChE (r = −0.79, *p* < 0.001), IL-6 (r = −0.92, *p* < 0.001), TNF-α (r = −0.93, *p* < 0.001), and MDA (r = −0.95, *p* < 0.001). The correlation network (Figure 7B) further illustrates the close relationships among behavioral performance, cholinergic dysfunction, inflammatory mediators, and oxidative stress biomarkers.

### 3.8. Molecular Docking of Phytochemicals from the Bioactive Oil Bend Nanoemulsion with TNF-α, IL-6, Acetylcholinesterase

A molecular docking study was performed to investigate the potential interactions of the major phytochemicals identified in the oil blend nanoemulsion, including the phytosterols β-sitosterol, campesterol, and stigmasterol, α-tocopherol, and the phenolic compounds catechin and gallic acid, with selected protein targets implicated in inflammation and depression, namely TNF-α, IL-6, and acetylcholinesterase (AChE). The binding free energies of the investigated compounds were presented in Table 7. The docking results demonstrated strong interactions between the investigated phytochemicals and the selected protein targets. Stigmasterol exhibited the highest binding affinity toward TNF-α (−8.5 kcal/mol), IL-6 (−7.1 kcal/mol), and AChE (−10.5 kcal/mol), followed closely by β-sitosterol and campesterol. Catechin also showed favorable binding affinities toward all investigated proteins, whereas gallic acid displayed comparatively weaker interactions. The high binding affinities of phytosterols and catechin toward inflammatory and cholinergic targets support their potential role in mediating the anti-inflammatory and neuroprotective effects of the oil blend nanoemulsion.

The 2-D and 3-D structures of the phytochemical compounds exhibiting the highest content in the oil blend nanoemulsion for TNF-α, IL-6, and AChE are shown in Table 8, Table 9 and Table 10. The binding modes revealed that the investigated phytochemicals interacted favorably with the active sites of TNF-α, IL-6, and AChE through a combination of hydrophobic, hydrogen-bonding, and electrostatic interactions. Within the TNF-α binding pocket, the phytosterols primarily established hydrophobic interactions, including alkyl, π-alkyl, and π–π stacked interactions. β-Sitosterol, campesterol, and α-tocopherol interacted with key residues TYR59, TYR119, and ILE155, whereas stigmasterol interacted predominantly with TYR59 and TYR119. In contrast, catechin and gallic acid exhibited additional hydrogen-bonding interactions. Catechin formed a conventional hydrogen bond with GLY121 and a π–π stacked interaction with TYR119, while gallic acid established hydrogen bonds with LEU93 and SER147 together with a π-alkyl interaction with VAL17.

Within the IL-6 active site, β-sitosterol and campesterol interacted mainly with TYR97, PRO139, and LEU147 residues. Stigmasterol exhibited hydrophobic interactions with LYS27, ARG30, TYR31, and VAL121. α-Tocopherol formed electrostatic and hydrophobic interactions with LYS46, ARG104, PHE105, and ASP160. Catechin displayed hydrogen-bonding and hydrophobic interactions with LEU92, GLU99, PRO139, PRO141, and ALA145, whereas gallic acid interacted with LEU62 and LEU165 through hydrogen bonding and π-sigma interactions.

For AChE, β-sitosterol, campesterol, and stigmasterol shared several key hydrophobic interactions involving TRP286, LEU289, PHE297, TYR337, PHE338, and TYR341 residues. Catechin and gallic acid formed multiple hydrogen bonds within the catalytic binding region. Catechin interacted with TRP286, TYR337, and TYR341, while gallic acid established hydrogen bonds with GLY120, TYR133, GLU202, and HIS447. Additional hydrophobic interactions were observed between catechin and AChE at the TYR124 and PHE295 residues, and between gallic acid and TRP86.

Overall, all investigated phytochemicals demonstrated favorable binding interactions with TNF-α, IL-6, and AChE. The observed interactions, mediated through hydrogen bonding, electrostatic attraction, and hydrophobic contacts, suggest that these bioactive compounds may contribute collectively to the anti-inflammatory and antidepressant effects of the oil blend nanoemulsion observed in the in vivo study.

**Figure 8:**
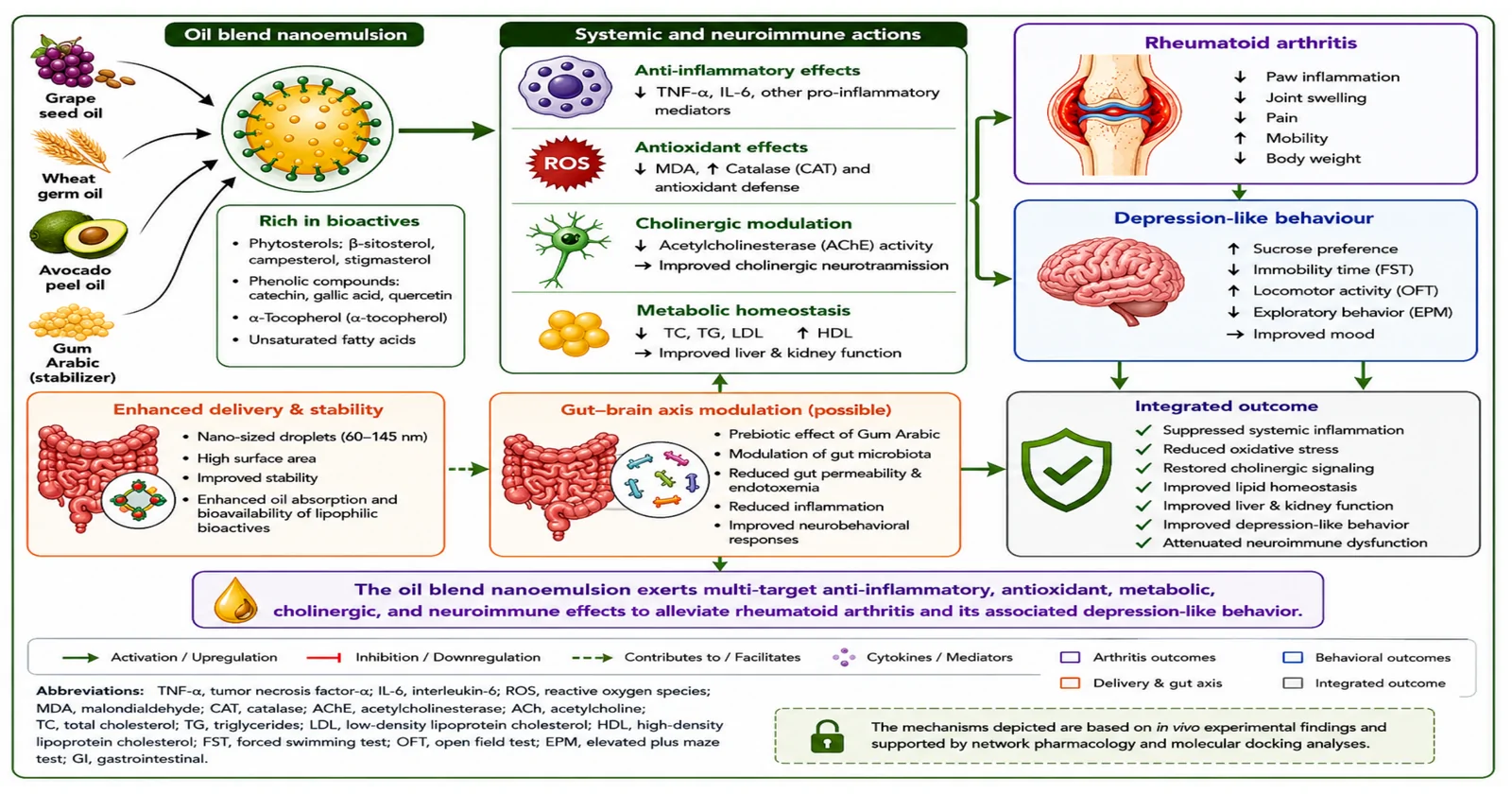
Proposed mechanism underlying the therapeutic effects of the Gum Arabic-stabilized bioactive oil blend nanoemulsion in rheumatoid arthritis-associated depression-like behavior. The lyophilized oil blend nanoemulsion, prepared from grape seed oil, wheat germ oil, and avocado peel oil, and stabilized with Gum Arabic, is rich in phenolic compounds, phytosterols, α-tocopherol, and unsaturated fatty acids. Nano-sized droplets (60–145 nm) enhance the stability, gastrointestinal absorption, and bioavailability of lipophilic bioactive compounds. The coordinated actions of these constituents suppress systemic inflammation by reducing TNF-α and IL-6, attenuate oxidative stress by decreasing malondialdehyde (MDA) and enhancing catalase (CAT) activity, improve cholinergic neurotransmission through inhibition of acetylcholinesterase (AChE), and restore metabolic homeostasis by improving lipid profile together with liver and kidney function. These effects alleviate paw inflammation, joint swelling, and pain while improving mobility and depression-like behavior, as demonstrated by increased sucrose preference, reduced immobility in the forced swimming test (FST), and enhanced locomotor and exploratory activities in the open field test (OFT) and elevated plus maze (EPM). Enhanced bioavailability provided by the nanoemulsion, together with the potential prebiotic effects of Gum Arabic and avocado peel oil, may also contribute to gut–brain axis modulation through improved gut microbial homeostasis, reduced intestinal permeability, and attenuation of inflammation, thereby supporting improved neurobehavioral responses. However, gut microbiota composition and gut–brain axis modulation were not directly investigated in the present study and therefore remain hypothetical. Overall, the bioactive oil blend nanoemulsion exerts complementary multi-target anti-inflammatory, antioxidant, metabolic, cholinergic, and neuroimmune effects that collectively contribute to the attenuation of rheumatoid arthritis and its associated depression-like behavior.

The experimental findings obtained in the present study are summarized in the proposed mechanistic model shown in Figure 8, which integrates the physicochemical properties of the nanoemulsion with the biochemical, behavioral, and molecular results.

### 3.9. Network Pharmacology Analysis of the Bioactive Oil Blend Nanoemulsion

**Figure 9:**
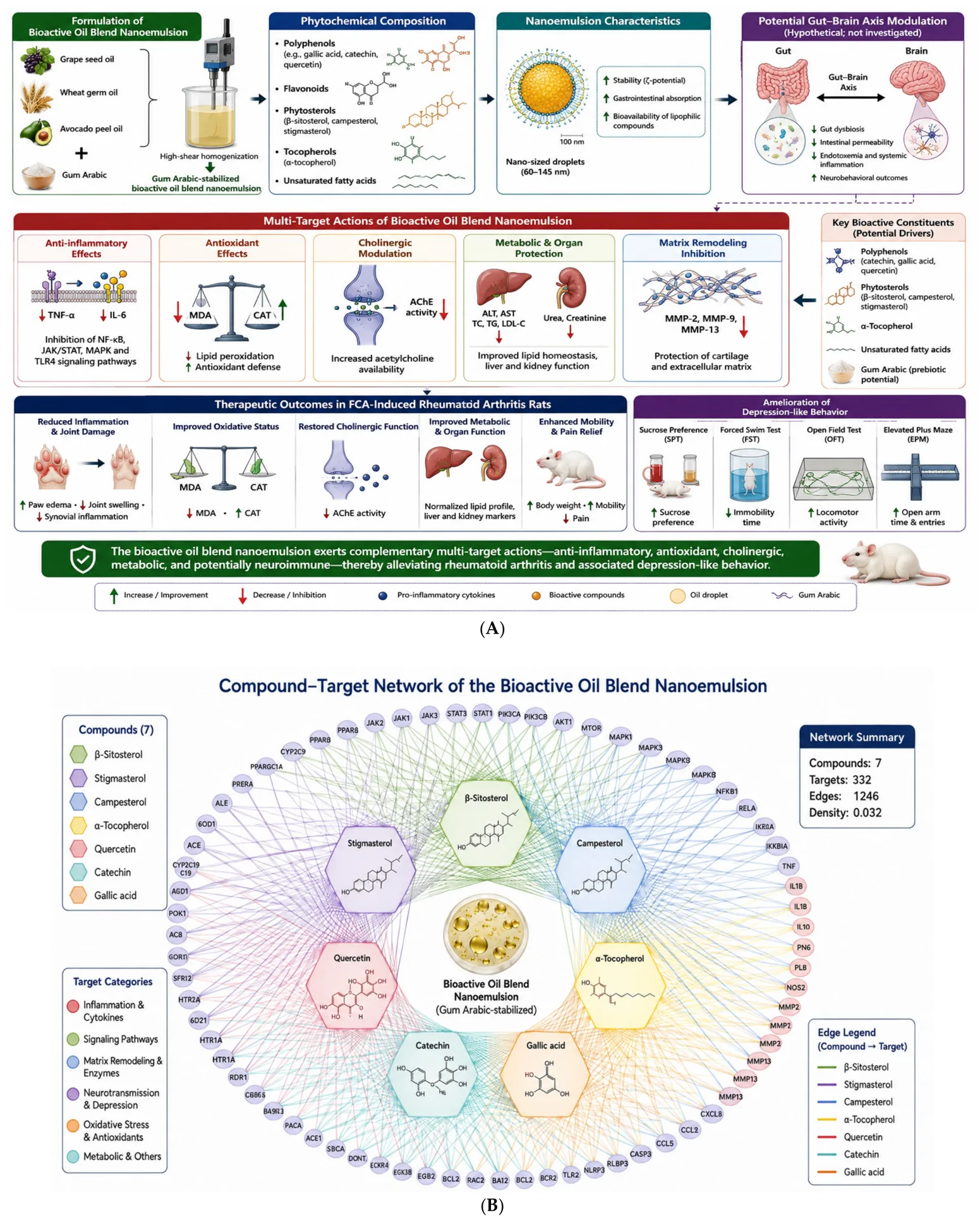

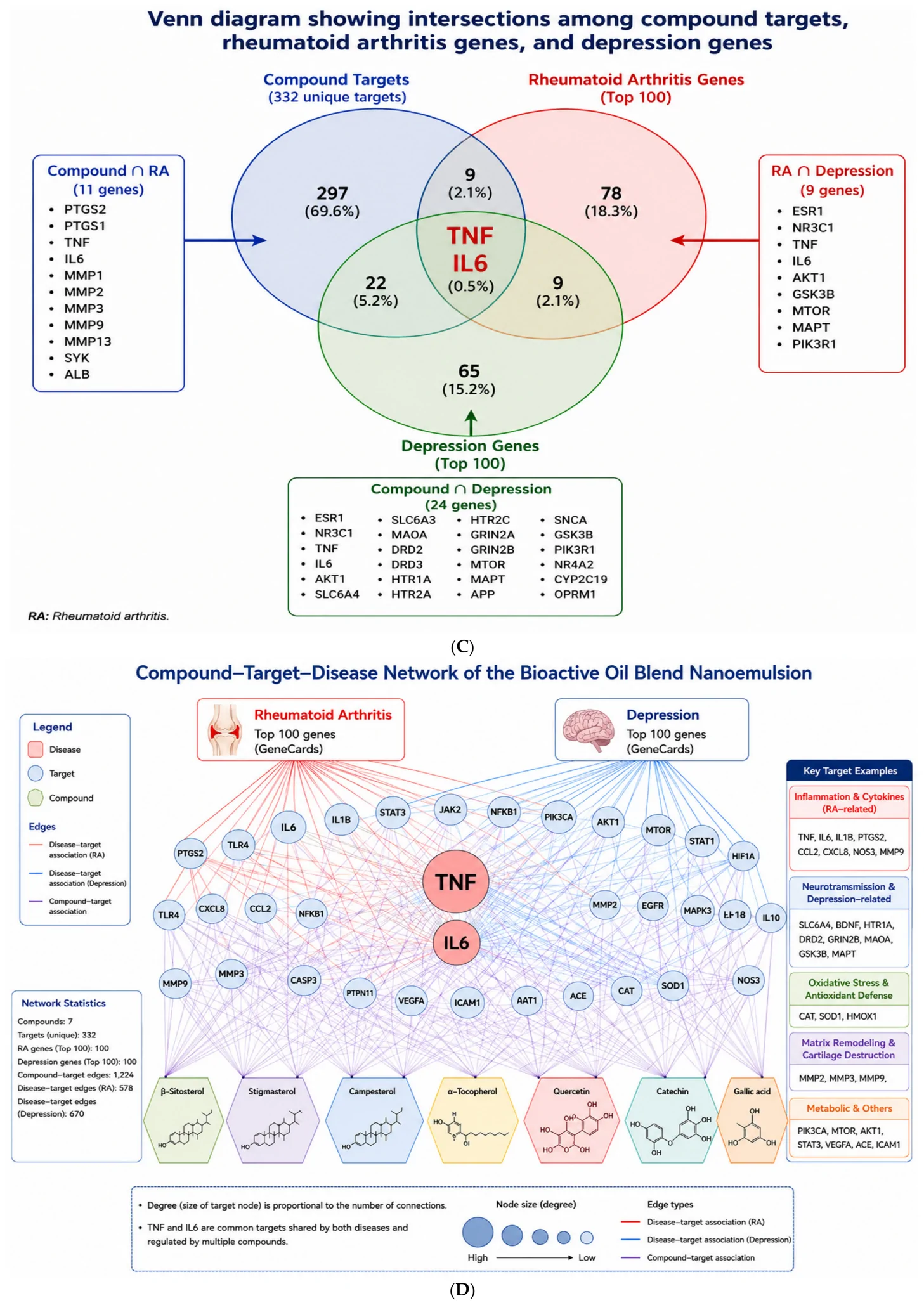

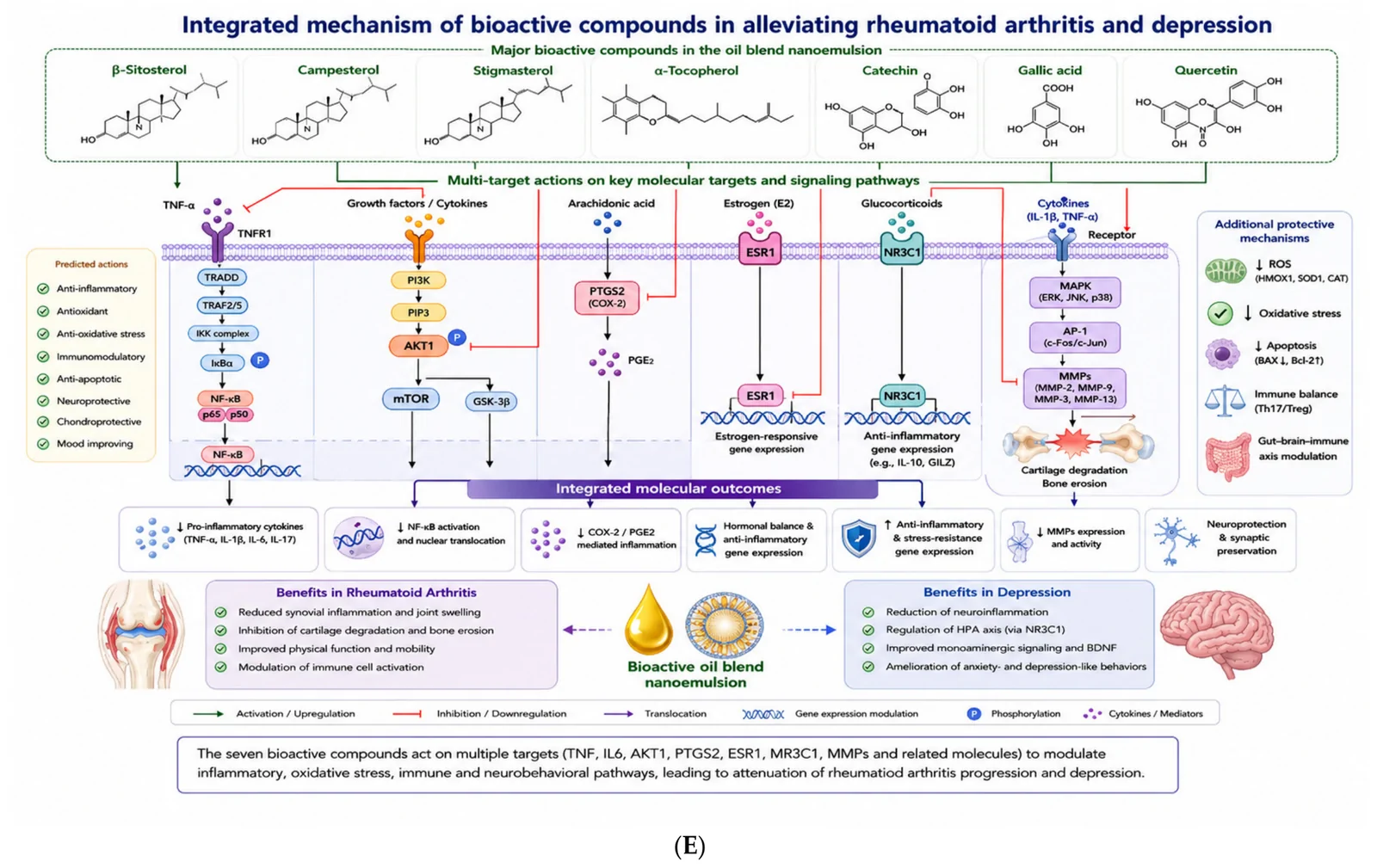
Integrated network pharmacology analysis of the Gum Arabic-stabilized bioactive oil blend nanoemulsion. (**A**) Overall workflow of the network pharmacology analysis. (**B**) Compound–target interaction network constructed using Cytoscape, showing the relationships between the seven major bioactive compounds (β-sitosterol, campesterol, stigmasterol, α-tocopherol, catechin, gallic acid, and quercetin) and their predicted protein targets. (**C**) Venn diagram illustrating the overlap among compound targets, rheumatoid arthritis (RA)-associated genes, and depression-associated genes. (**D**) Compound–target–disease interaction network integrating the seven bioactive compounds, common therapeutic targets, and the two diseases. (**E**) Integrated mechanistic diagram illustrating the proposed molecular mechanisms by which the Gum Arabic-stabilized bioactive oil blend nanoemulsion attenuates rheumatoid arthritis-associated depression through the coordinated regulation of inflammatory, oxidative stress, neuroimmune, and intracellular signaling pathways.

To further elucidate the molecular mechanisms underlying the anti-rheumatoid arthritis and antidepressant activities of the Gum Arabic-stabilized bioactive oil blend nanoemulsion, an integrated network pharmacology analysis was performed (Figure 9A). Based on the phytochemical characterization, seven representative bioactive compounds—β-sitosterol, campesterol, stigmasterol, α-tocopherol, catechin, gallic acid, and quercetin—were selected for target prediction.

SwissTargetPrediction identified the potential human protein targets of the seven compounds. After merging the predicted targets and removing duplicate entries, 332 unique protein targets were obtained, demonstrating the broad pharmacological potential of the identified phytochemicals. The compound–target interaction network (Figure 9B) revealed the characteristic multi-component–multi-target profile of the nanoemulsion, with each phytochemical interacting with multiple protein targets.

To identify disease-related targets, the top 100 rheumatoid arthritis-associated genes and the top 100 depression-associated genes were retrieved from the GeneCards database. An intersection analysis identified several common therapeutic targets shared between the predicted compound targets and both diseases (Figure 9C). The overlapping genes included TNF, IL6, PTGS2, AKT1, ESR1, NR3C1, STAT3, JAK2, IL1B, NFKB1, TLR4, CXCL8, MMP9, MMP3, and MMP2, suggesting that these proteins may represent key molecular targets involved in the therapeutic effects of the nanoemulsion. The compound–target–disease network (Figure 9D) further illustrated the complex interactions among the seven bioactive compounds, the common therapeutic targets, and the two diseases.

Finally, the network pharmacology findings were integrated with the molecular docking and experimental results to generate a comprehensive mechanistic model (Figure 9E). The integrated analysis suggests that β-sitosterol, campesterol, stigmasterol, α-tocopherol, catechin, gallic acid, and quercetin collectively modulate key inflammatory and neuroimmune regulators, particularly TNF, AKT1, PTGS2, ESR1, NR3C1, IL6, STAT3, JAK2, and matrix metalloproteinases (MMPs). Together with the favorable molecular docking results, these findings provide mechanistic support for a multi-component, multi-target, multi-pathway mechanism underlying the therapeutic effects of the Gum Arabic-stabilized bioactive oil blend nanoemulsion.

## 4. Discussion

### 4.1. Nanoemulsion Characterization, Phytochemical Composition, Oxidative Stability, and Anti-Inflammatory Activity

The present study demonstrated that the Gum Arabic-stabilized bioactive oil blend nanoemulsion effectively attenuated adjuvant-induced rheumatoid arthritis and its associated depression-like behavior in female rats. These beneficial effects are likely attributable to the favorable physicochemical characteristics of the nanoemulsion, the enhanced oral delivery of lipophilic phytochemicals, and the complementary biological activities of its naturally occurring constituents. Nanoemulsion technology is well recognized for improving the stability, dispersion, gastrointestinal absorption, and bioavailability of lipophilic bioactive compounds, thereby enhancing their therapeutic efficacy [13,14,15,20].

Physicochemical characterization confirmed the successful preparation of a stable nanoemulsion with predominantly spherical droplets (60–145 nm), a narrow particle size distribution, and a negative zeta potential (−14.88 mV), indicating good formulation homogeneity and colloidal stability. The small droplet size increases the interfacial surface area, facilitating gastrointestinal digestion and absorption of lipophilic compounds, while Gum Arabic provides additional steric stabilization that helps maintain emulsion stability [35,36].

Besides acting as a natural emulsifier, Gum Arabic may also contribute to the biological activity of the formulation. Previous studies have shown that it possesses antioxidant, anti-inflammatory, and immunomodulatory properties, and that it suppresses the production of pro-inflammatory cytokines such as TNF-α and IL-6 [21,22,42,47]. Its ability to protect encapsulated phytochemicals from degradation may further enhance their stability and bioavailability, suggesting that the therapeutic effects observed in the present study resulted from the combined actions of the bioactive oils and Gum Arabic.

Phytochemical analysis showed that the nanoemulsion was rich in phenolic compounds, flavonoids, phytosterols, tocopherols, and unsaturated fatty acids. Major phenolics, including catechin, gallic acid, chlorogenic acid, quercetin, rutin, and ferulic acid, are widely recognized for their antioxidant, anti-inflammatory, and neuroprotective properties through the modulation of pathways such as NF-κB and Nrf2 [11,47,48,49,50,51,52]. Their complementary actions likely contributed to the simultaneous improvements in inflammatory biomarkers, oxidative stress, cholinergic activity, and behavioral performance, supporting the concept that botanical mixtures exert therapeutic effects through coordinated modulation of multiple interconnected pathways rather than a single molecular target [13,14].

The relatively high α-tocopherol content further enhanced the nutritional value and oxidative stability of the nanoemulsion. As a major lipid-soluble antioxidant, α-tocopherol protects polyunsaturated fatty acids against lipid peroxidation, which is consistent with the low peroxide values observed during refrigerated storage. Together with phenolic compounds, flavonoids, and nanoencapsulation, it likely contributed to the preservation of the integrity and biological activity of the oil blend.

One of the most notable findings was the marked reduction in paw inflammation, with the high-dose nanoemulsion decreasing paw edema by approximately 70%. This anti-inflammatory effect is consistent with the known activities of polyphenols, phytosterols, tocopherols, and unsaturated fatty acids, which suppress inflammatory cytokine production, reduce oxidative stress, and regulate signaling pathways involved in rheumatoid arthritis [17,18,19,47,48,49,50,51,52]. The concurrent improvement in paw morphology further supports the quantitative reduction in inflammation and may contribute to the attenuation of depression-like behavior by reducing peripheral inflammatory signals that influence the central nervous system.

The present findings also extend our previous work on the valorization of agro-industrial by-products as sources of functional ingredients. Earlier studies demonstrated the anti-inflammatory and antioxidant properties of grape seed, wheat germ, and avocado peel-derived nanoformulations [23,24,25]. The present study further shows that combining these bioactive oils into a single Gum Arabic-stabilized nanoemulsion integrates their complementary phytochemical profiles and enhances their therapeutic potential. This supports the development of sustainable botanical nanoformulations for the multi-target management of rheumatoid arthritis and its associated neurobehavioral complications.

### 4.2. Nutritional Significance, Biological Activity, Depression-like Behavior, and Correlation Analysis

Beyond reducing joint inflammation, the bioactive oil blend nanoemulsion significantly improved the systemic metabolic disturbances associated with rheumatoid arthritis. Treatment favorably modulated the lipid profile by lowering total cholesterol, triglycerides, and LDL-cholesterol while increasing HDL-cholesterol, and it also normalized liver (ALT and AST) and kidney (urea and creatinine) function markers. These findings indicate that the nanoemulsion provided broad systemic protection in addition to its anti-inflammatory effects. Similar benefits have been reported for phytosterols, polyphenols, tocopherols, and unsaturated fatty acids, which regulate lipid metabolism, suppress inflammatory signaling, and protect hepatic and renal function during chronic inflammatory conditions [16,17,18,19,47].

The metabolic improvements are likely attributable to the complementary nutritional composition of the oil blend. Unsaturated fatty acids support membrane integrity and inflammatory resolution, phytosterols improve cholesterol homeostasis, and tocopherols and phenolic compounds protect against oxidative damage [16,17,18,19,47]. The incorporation of these bioactive compounds into a nanoemulsion likely enhanced their oral bioavailability, contributing to the observed systemic benefits.

A major finding of this study was the marked attenuation of depression-like behaviors following nanoemulsion treatment. Arthritic rats exhibited impaired sucrose preference, increased immobility in the forced swimming test, and reduced locomotor and exploratory activities, all of which were substantially improved, particularly in the high-dose group. These behavioral improvements are consistent with growing evidence that depression associated with rheumatoid arthritis is driven, at least in part, by chronic systemic inflammation and neuroimmune dysfunction rather than solely by persistent pain [3,4,5,6,7,8,9,10,53,54,55,56,57].

The behavioral recovery closely paralleled reductions in circulating TNF-α, IL-6, and malondialdehyde, together with restoration of catalase activity, suggesting that suppression of systemic inflammation and oxidative stress contributed to the improvement in neurobehavioral function. In addition, the significant reduction in acetylcholinesterase activity suggests activation of the cholinergic anti-inflammatory pathway, an important regulator of immune homeostasis and neuroimmune communication. Restoration of cholinergic signaling may therefore represent another mechanism contributing to both the anti-inflammatory and antidepressant-like effects of the nanoemulsion [12,58,59,60,61].

The broad biological activity of the nanoemulsion is most likely the result of complementary interactions among its phytochemicals rather than the action of a single constituent. Catechin, gallic acid, chlorogenic acid, quercetin, rutin, ferulic acid, phytosterols, tocopherols, and unsaturated fatty acids have all been reported to possess antioxidant, anti-inflammatory, immunomodulatory, and neuroprotective properties [11,16,17,18,19,47,48,49,50,51,52]. Nanoemulsion technology may further enhance these effects by improving the stability and bioavailability of these compounds, thus allowing coordinated modulation of multiple pathological pathways [13,14,15,20].

Correlation analysis further supported the proposed mechanism by demonstrating strong associations between improvements in behavioral performance and reductions in inflammatory cytokines, oxidative stress biomarkers, and acetylcholinesterase activity levels. These findings reinforce the close relationship between systemic inflammation, oxidative stress, cholinergic dysfunction, and depression-like behavior in rheumatoid arthritis.

Finally, the present results are consistent with our previous studies demonstrating the anti-inflammatory and antioxidant activities of wheat germ oil, chia seed oil, avocado peel-derived bioactive formulations, and other plant-derived nutraceuticals in experimental arthritis [16,23,24,25,62,63,64,65,66]. The current study extends these findings by showing that integrating these bioactive oils into a single nanoemulsion not only attenuates arthritis but also improves depression-like behavior, thereby highlighting the potential of sustainable agro-industrial by-product-derived nanoformulations as complementary multi-target interventions for rheumatoid arthritis and its associated neurobehavioral complications.

### 4.3. Molecular Docking, Gut–Brain Axis, and Proposed Mechanisms

Molecular docking was performed to further investigate the molecular basis of the therapeutic effects of the bioactive oil blend nanoemulsion. The major phytochemicals identified by HPLC showed favorable binding affinities toward TNF-α, IL-6, and acetylcholinesterase (AChE), supporting their potential to modulate inflammatory and cholinergic signaling pathways. Although docking cannot confirm biological activity, it provides complementary mechanistic evidence that is consistent with the biochemical and behavioral findings observed in vivo. Integrating phytochemical characterization with molecular docking has become a valuable approach for elucidating the mechanisms of complex botanical formulations [13,14,17,18,20].

Among the identified compounds, phytosterols exhibited the strongest predicted binding affinities, followed by phenolic compounds such as catechin, chlorogenic acid, quercetin, rutin, gallic acid, and ferulic acid. These findings agree with previous reports demonstrating that phytosterols and polyphenols suppress inflammatory cytokine production, reduce oxidative stress, and regulate signaling pathways involved in chronic inflammatory and neurobehavioral disorders [11,17,18,19,47,48,49,50,51,52]. The results further support the concept that the therapeutic efficacy of the nanoemulsion arises from complementary interactions among multiple bioactive constituents, rather than from a single active compound.

An additional mechanism that may contribute to the observed therapeutic effects is modulation of the gut–brain axis. Dietary polyphenols, phytosterols, unsaturated fatty acids, and Gum Arabic have been reported to improve gut microbial composition, intestinal barrier function, and immune homeostasis, thereby influencing both systemic inflammation and brain function [2,15,22,46,51,66]. Although gut microbiota was not evaluated in the present study, our previous work demonstrated that an avocado peel-derived nanoformulation improved gut microbial composition in arthritic rats using 16S rRNA sequencing [25]. Therefore, gut microbiota modulation may have contributed to the improvement in depression-like behavior; however, this mechanism remains hypothetical and requires direct experimental confirmation.

Based on the overall findings, a comprehensive mechanism of action is proposed (Figure 8). The nanoemulsion enhances the stability and oral delivery of lipophilic phytochemicals, allowing phenolic compounds, phytosterols, tocopherols, and unsaturated fatty acids to act synergistically by suppressing inflammatory cytokines, reducing oxidative stress, and improving cholinergic neurotransmission. These coordinated actions attenuate systemic inflammation and improve neurobehavioral function, thereby alleviating depression-like behavior associated with experimental rheumatoid arthritis. The significant correlations among inflammatory biomarkers, oxidative stress indices, acetylcholinesterase activity, and behavioral outcomes further support this integrated mechanism.

### 4.4. Network Pharmacological Analysis

Network pharmacology complemented the molecular docking analysis by providing a system-level understanding of the therapeutic mechanisms of the Gum Arabic–stabilized bioactive oil blend nanoemulsion. The identification of 332 unique protein targets for the seven representative phytochemicals highlights the multi-target nature of the formulation and supports the concept that botanical mixtures exert their biological effects through the coordinated regulation of multiple signaling pathways rather than a single molecular target.

Intersection analysis of rheumatoid arthritis- and depression-associated genes identified several common therapeutic targets, including TNF, IL6, AKT1, PTGS2, ESR1, NR3C1, STAT3, JAK2, IL1B, NFKB1, MMP9, and TLR4. These proteins play central roles in inflammation, oxidative stress, immune activation, extracellular matrix remodeling, and neuroimmune communication, providing a mechanistic link between rheumatoid arthritis and its associated depression.

The enrichment of inflammation- and neuroimmune-related signaling pathways further supports the biological relevance of these targets and provides a mechanistic explanation for the observed reductions in inflammatory cytokines, oxidative stress, and depression-like behavior.

Overall, the network pharmacology findings were consistent with the molecular docking and experimental results. The identified hub genes and enriched pathways, together with the observed reductions in inflammatory cytokines, oxidative stress, acetylcholinesterase activity, and depression-like behavior, support a multi-component–multi-target–multi-pathway mechanism of action. These findings provide mechanistic insight into the complementary effects of the phytosterols, phenolic compounds, and α-tocopherol present in the nanoemulsion and further support its potential as a complementary nutraceutical strategy for rheumatoid arthritis-associated depression. Nevertheless, transcriptomic, proteomic, and gene expression studies are warranted to experimentally validate the predicted molecular targets and signaling pathways.

## 5. Study Limitations

The present study has several limitations that should be acknowledged. First, although the proposed mechanisms were supported by biochemical findings, molecular docking, and network pharmacology analyses, direct experimental validation of the predicted intracellular signaling pathways was not performed. Future studies should investigate key molecular pathways, including NF-κB, Nrf2, MAPK, PI3K/Akt, and cholinergic signaling, using gene and protein expression analyses to further elucidate the mechanisms underlying the observed therapeutic effects.

Second, improvements in depression-like behavior were evaluated using behavioral tests together with peripheral inflammatory, oxidative stress, and cholinergic biomarkers. However, brain-specific biochemical, molecular, and histopathological alterations were not assessed. Future studies should therefore investigate neuroinflammation, neurotransmitter homeostasis, synaptic plasticity, and neuronal integrity to provide direct evidence of the central mechanisms involved.

Third, although our previous study demonstrated that an avocado peel-derived bioactive nanoformulation favorably modulated the gut microbiota, the gut microbial composition and gut–brain axis interactions were not investigated in the present study. Therefore, the contribution of microbiota-mediated mechanisms to the observed neurobehavioral improvements remains hypothetical and requires direct experimental validation.

In addition, paw thickness was assessed only before FCA administration and at the end of the treatment period; therefore, intermediate measurements were not available to characterize the temporal progression of paw inflammation or the response to treatment over time.

Finally, this study was conducted in an experimental animal model. Further pharmacokinetic, toxicological, and well-designed clinical studies are required to establish the long-term safety, optimal dosage, and clinical efficacy of the Gum Arabic–stabilized bioactive oil blend nanoemulsion before its potential translation into human applications.

## 6. Conclusions

The present study demonstrated that a Gum Arabic-stabilized bioactive oil blend nanoemulsion, prepared from grape seed oil, wheat germ oil, and avocado peel oil, effectively attenuated adjuvant-induced rheumatoid arthritis and its associated depression-like behavior in female rats. The nanoemulsion combined favorable physicochemical characteristics with a rich composition of phenolic compounds, flavonoids, phytosterols, tocopherols, and unsaturated fatty acids, which collectively contributed to its therapeutic activity. Treatment significantly reduced joint inflammation, pro-inflammatory cytokines, oxidative stress, and acetylcholinesterase activity, while enhancing antioxidant defenses, improving metabolic homeostasis, and restoring behavioral performance. Molecular docking and network pharmacology analyses further supported the experimental findings by identifying key molecular targets and signaling pathways associated with inflammation, oxidative stress, and neuroimmune regulation.

Overall, the findings suggest that the bioactive oil blend nanoemulsion alleviates rheumatoid arthritis-associated depression-like behavior through the coordinated modulation of inflammatory, oxidative stress, cholinergic, and neuroimmune pathways via a multi-component–multi-target–multi-pathway mechanism. These results highlight the potential of standardized botanical nanoformulations as complementary therapeutic strategies for immune-mediated neurobehavioral complications associated with rheumatoid arthritis. Further pharmacokinetic, toxicological, mechanistic, and clinical studies are warranted to establish their long-term safety, optimize dosages, and evaluate their translational potential.

## Figures and Tables

**Table 1:** Total phenolic content, total flavonoid content, and HPLC profiles of the prepared oil blend nanoemulsion.

Components	Concentration
Total phenolics (mg GAE/100 g)	320 ± 2.542
Total flavonoids (mg/QE/100 g)	145 ± 1.638
Phenolic compounds profile (µg/g)	
Catechin	600 ± 0.068
Gallic acid	270 ± 0.031
Chlorogenic acid	210 ± 0.025
Methyl gallate	40 ± 0.037
Caffeic acid	80 ± 0.045
Syringic acid	50 ± 0.027
Rutin	155 ± 0.030
p-Coumaric acid	65 ± 0.038
Pyrocatechol	30 ± 0.028
Cinnamic acid	7 ± 0.005
Vanillin	11 ± 0.015
Ferulic acid	140 ± 0.075
Naringenin	25 ± 0.011
Hesperetin	10 ± 0.008
Kaempferol	90 ± 0.055
Quercetin	170 ± 0.065

**Table 2:** Fatty acid composition and phytosterol profile of the prepared oil blend nanoemulsion.

Fatty Acids and Phytosterol Content	Nanoemulsion
Fatty acids (as percentage of total fatty acids)	
Palmitic acid: C16:0	13.15 ± 0.011
Stearic C18:0	1.89 ± 0.002
Oleic acid: C18:1	44.5 ± 0.252
Linoleic acid (C18:2) (ω − 6)	32.9 ± 0.551
Linolenic acid (C18:3) (ω − 3)	4.1 ± 0.066
Total identified saturated fatty acids	15.04 ± 0.055
Total identified unsaturated fatty acids	81.5 ± 0.086
Phytosterols (as percentage of total phytosterols)	
Campesterol	12.3 ± 0.080
Stigmasterol	11.9 ± 0.040
β-Sitosterol	46.6 ± 0.224
Total phytosterols	70.8 ± 0.214
α-Tocopherols (mg/100 g)	650 ± 4.721
COX = (C18:1 + 10.3 × C18:2 + 21.6 × C18:3)/100	4.72 ± 0.132

**Table 3:** Changes in pH and peroxide values of the prepared oil blend nanoemulsion during refrigerated storage.

Parameters	Time			
	Initial	First Week	Second Week	Third Week
pH	6.03 ^b^ ± 0.058	6.12 ^ab^ ± 0.029	6.14 ^ab^ ± 0.035	6.18 ^a^ ± 0.025
Peroxide Value meq O_2_/kg oil	2.35 ^c^ ± 0.050	2.43 ^c^ ± 0.076	2.57 ^b^ ± 0.076	2.76 ^a^ ± 0.066

Values are expressed as mean ± SD (*n* = 3). Statistical significance was analyzed using one-way ANOVA followed by Tukey’s multiple comparison post hoc test across all groups. Different superscript letters within the same row indicate significant differences at *p* ≤ 0.05, while groups sharing the same letter show no statistically significant difference (*p* ≥ 0.05).

**Table 4:** Effects of the prepared oil blend nanoemulsion on acetylcholine (AChE), inflammatory cytokines, and oxidative stress biomarkers in adjuvant-induced arthritic rats.

Parameters	Normal Control	Arthritic Control	Oil Blend Nanoemulsion Low Dose	Oil Blend Nanoemulsion High Dose
AChE (ng/mL)	1.22 ^a^ ± 0.079	2.57 ^b^ ± 0.084	1.75 ^c^ ± 0.187	1.33 ^ac^ ± 0.088
IL-6 (pg/mL)	57.50 ^a^ ± 1.12	84.17 ^b^ ± 1.30	70.67 ^c^ ± 1.02	62.50 ^d^ ± 1.06
TNF-α (pg/mL)	13.67 ^a^ ± 0.42	35.33 ^b^ ± 0.84	24.67 ^c^ ± 0.56	18.67 ^d^ ± 0.49
MDA (nmol/mL)	7.17 ^a^ ± 0.25	19.50 ^b^ ± 1.06	14.33 ^c^ ± 0.67	11.25 ^d^ ± 0.44
CAT (U/L)	610.83 ^a^ ± 7.00	298.33 ^b^ ± 9.46	501.67 ^c^ ± 7.03	555.00 ^d^ ± 7.64

Values are expressed as mean ± SE (*n* = 6). Statistical significance was assessed using one-way ANOVA followed by Tukey’s multiple-comparison post hoc test across all groups. Groups with different superscript letters within the same row indicate significant differences (*p* ≤ 0.05), whereas groups sharing at least one letter are not significantly different (*p* > 0.05).

**Table 5:** Plasma lipid profiles and hepatic and renal functions of arthritic and normal rats.

Parameters	Normal Control	Arthritic Control	Oil Blend Nanoemulsion Low Dose	Oil Blend Nanoemulsion High Dose
T-Ch (mg/dL)	67.3 ^a^ ± 1.82	84.2 ^b^ ± 2.15	68.2 ^a^ ± 1.19	66.8 ^a^ ± 1.19
TG (mg/dL)	76.0 ^a^ ± 3.73	113.0 ^b^ ± 4.90	76.0 ^a^ ± 4.51	80.0 ^a^ ± 2.89
HDL-Ch (mg/dL)	36.8 ^a^ ± 1.08	29.3 ^b^ ± 0.71	35.0 ^a^ ± 1.06	38.5 ^a^ ± 0.99
LDL-Ch (mg/dL)	15.3 ^a^ ± 2.04	32.2 ^b^ ± 2.60	17.9 ^a^ ± 1.99	12.3 ^a^ ± 2.06
AST (IU/L)	58.9 ^a^ ± 1.18	94.3 ^b^ ± 5.4	59.4 ^a^ ± 3.77	60.0 ^a^ ± 1.93
ALT (IU/L)	12 ^a^ ± 0.58	16.8 ^b^ ± 0.60	12.83 ^a^ ± 1.24	12.5 ^a^ ± 0.43
Urea (mg/dL)	36 ^a^ ± 1.77	49 ^b^ ± 3.47	42 ^c^ ± 1.51	37 ^ac^ ± 2.54
Creatinine (mg/dL)	0.48 ^a^ ± 0.03	0.69 ^b^ ± 0.02	0.54 ^a^ ± 0.02	0.52 ^a^ ± 0.02

Values are expressed as mean ± SE (*n* = 6). Statistical significance was assessed using one-way ANOVA followed by Tukey’s multiple-comparison post hoc test across all groups. Groups with different superscript letters within the same row indicate significant differences (*p* ≤ 0.05), whereas groups sharing at least one letter are not significantly different (*p* > 0.05).

**Table 6:** Effect of the prepared oil blend nanoemulsion on body weight parameters and relative liver weight in adjuvant-induced arthritic rats.

Group	Initial Body Weight (g)	Final Body Weight (g)	Body Weight Gain (g)	Relative Liver
Normal control	147.00 ^a^ ± 2.633	200.83 ^a^ ± 6.67	53.83 ^a^ ± 8.733	3.32 ^b^ ± 0.12
Arthritic control	146.67 ^a^ ± 4.484	167.17 ^b^ ± 3.718	20.50 ^b^ ± 6.242	3.98 ^a^ ± 0.315
Oil blend nanoemulsion low dose	146.5 ^a^ ± 7.035	194.50 ^a^ ± 3.972	48.00 ^a^ ± 5.278	3.79 ^a^ ± 0.411
Oil blend nanoemulsion high dose	146.5 ^a^ ± 5.376	196.17 ^a^ ± 6.451	49.67 ^a^ ± 4.666	3.75 ^a^ ± 0.229

Values are expressed as mean ± SE (*n* = 6). Statistical significance was assessed using one-way ANOVA followed by Tukey’s multiple-comparison post hoc test across all groups. Groups with different superscript letters within the same column indicate significant differences (*p* ≤ 0.05), whereas groups sharing at least one letter are not significantly different (*p* > 0.05).

**Table 7:** Binding free energies (ΔG, kcal/mol) of major phytochemicals identified in the oil blend nanoemulsion against TNF-α, IL-6, and acetylcholinesterase (AChE).

Ligand	TNFα	IL-6	AChE
	Binding Free Energy (kcal/mol)		
β-Sitosterol	−8.4	−6.6	−10.5
Campesterol	−8.3	−6.7	−10.4
Stigmasterol	−8.5	−7.1	−10.5
α-Tocopherol	−7.8	−5.2	−10.4
Catechin	−7.1	−6.2	−9.1
Gallic acid	−5.5	−4.9	−6.4

**Table 8:** 2D and 3D interactions between each compound and TNFα.

Ligand	TNFα	
	2D	3D
β-Sitosterol	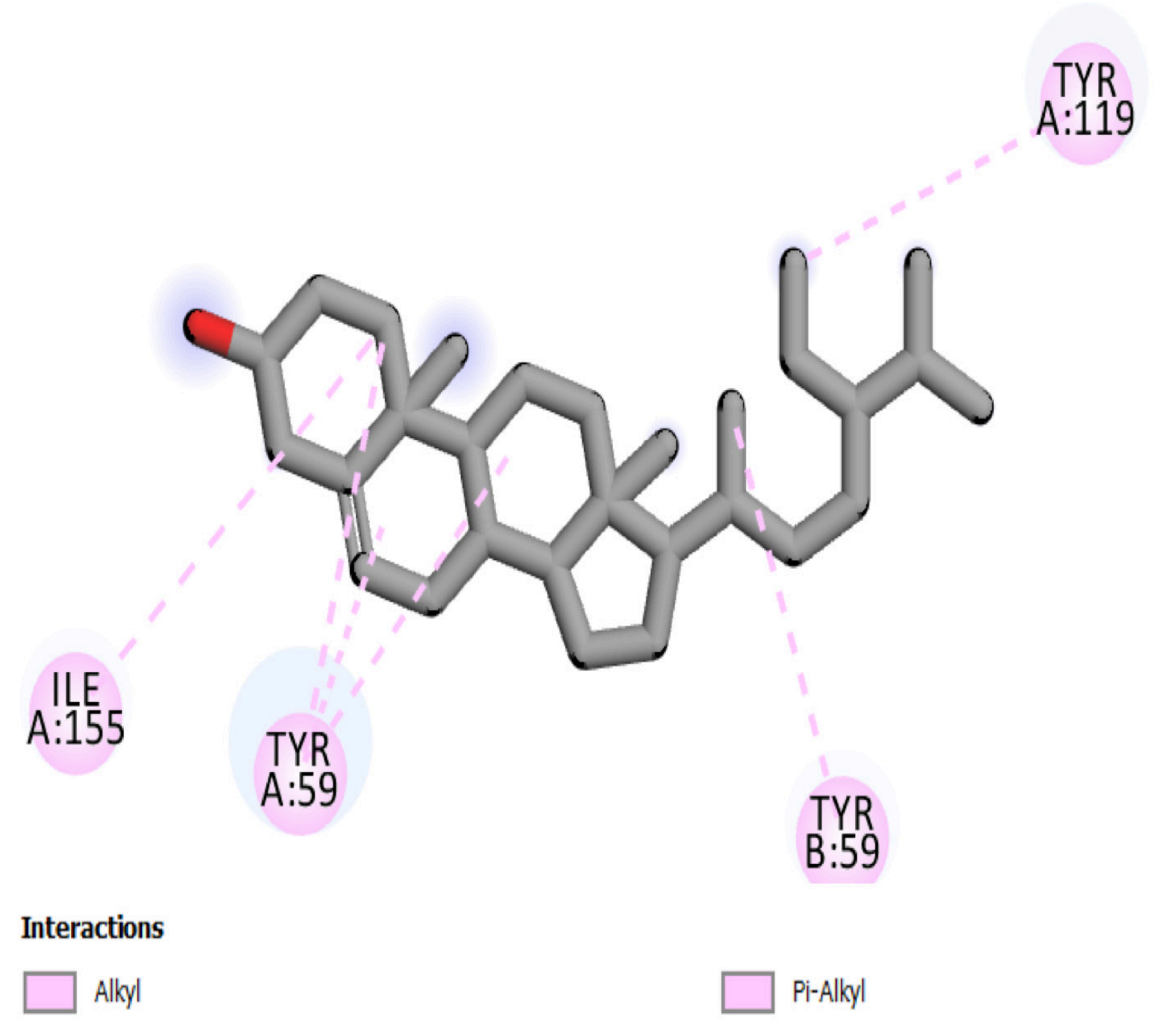	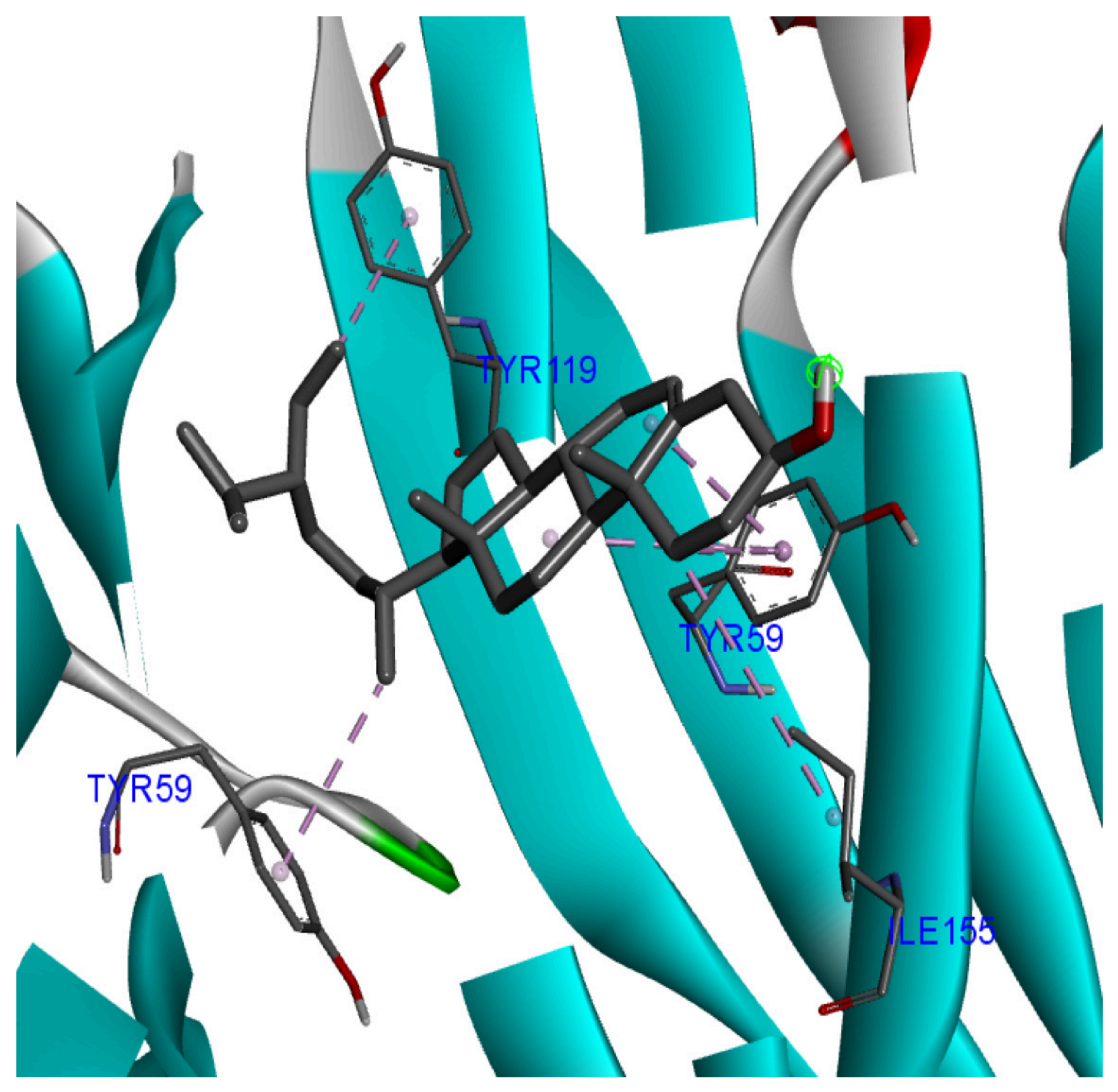
Campesterol	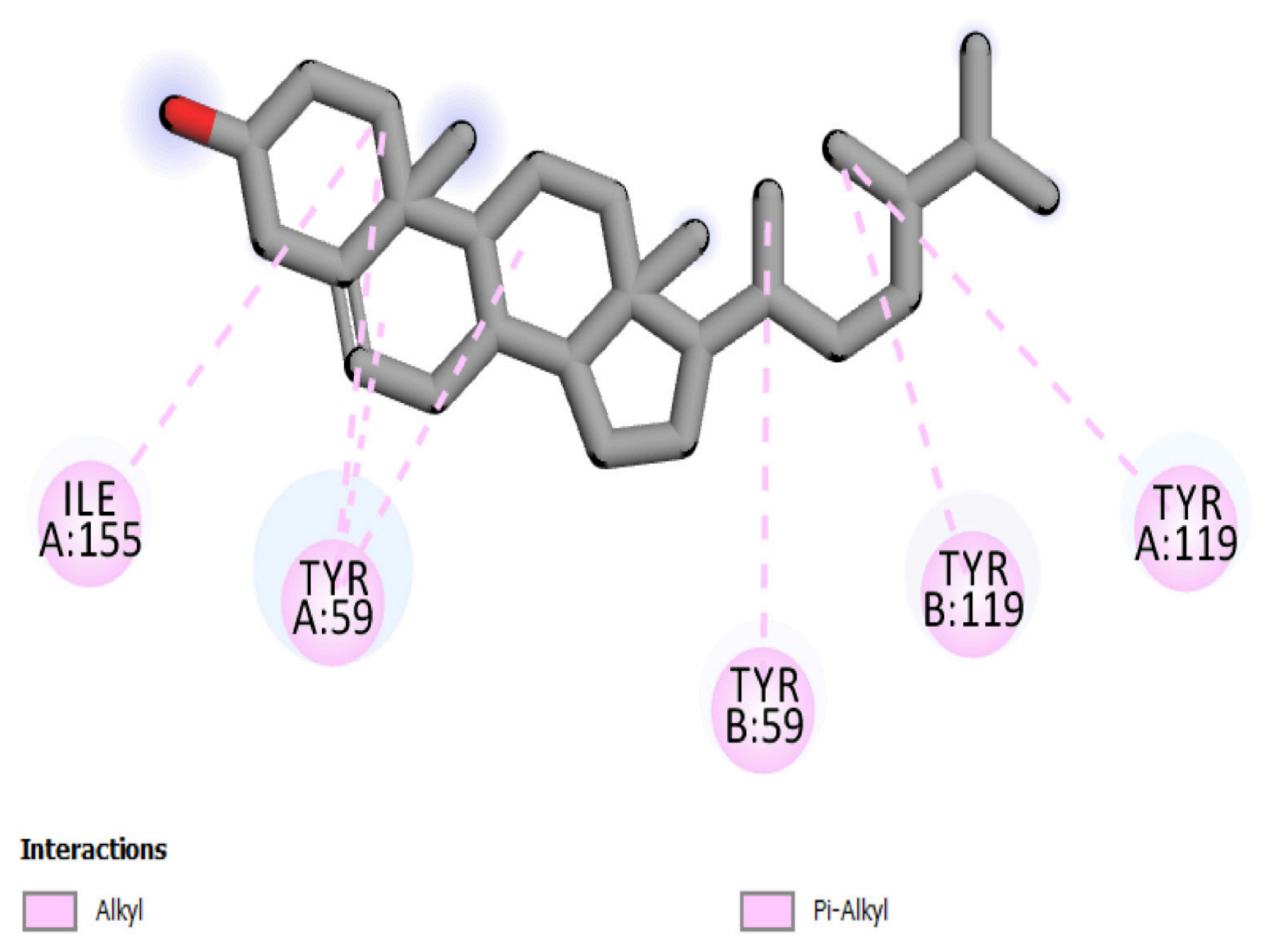	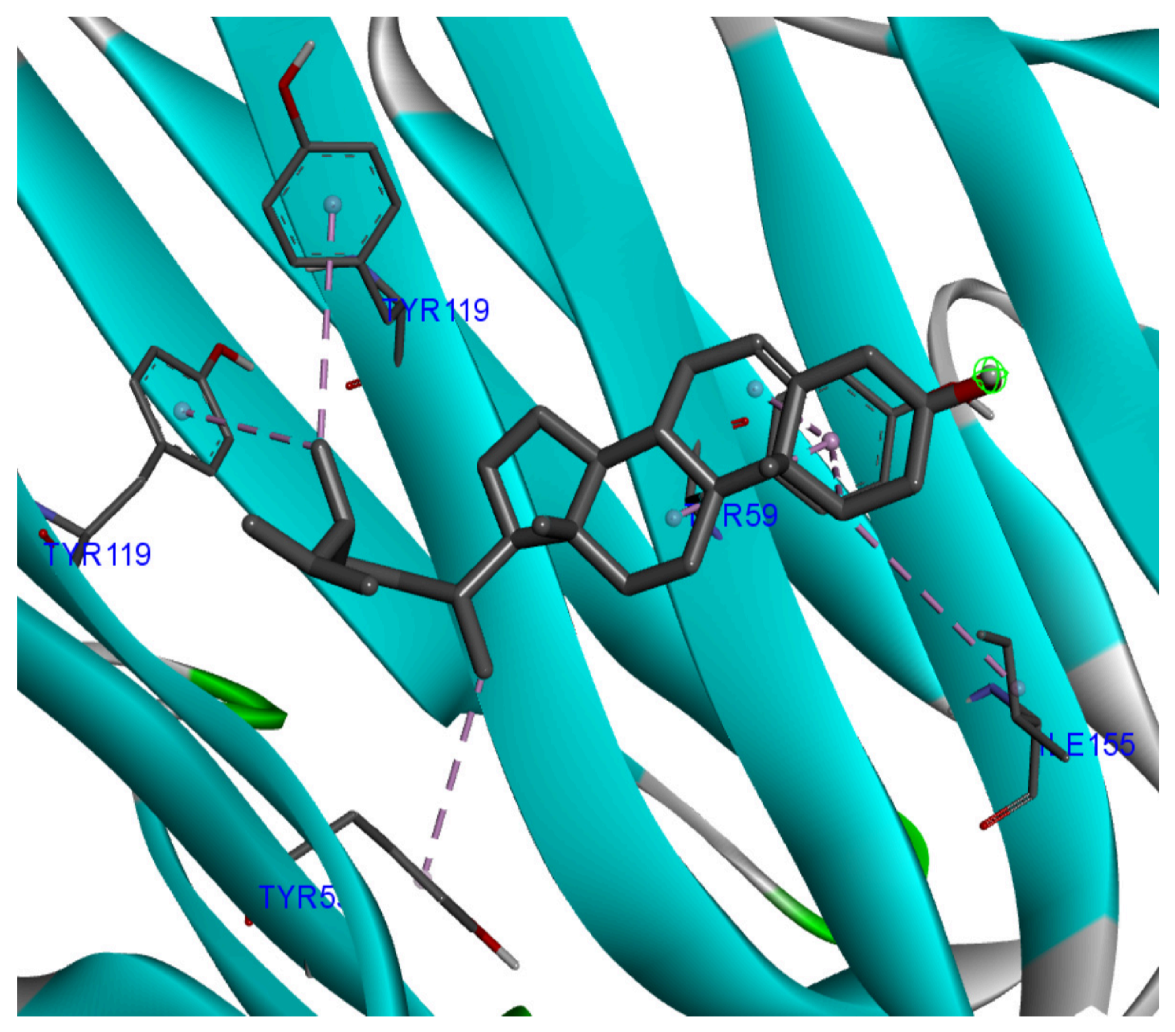
Stigmasterol	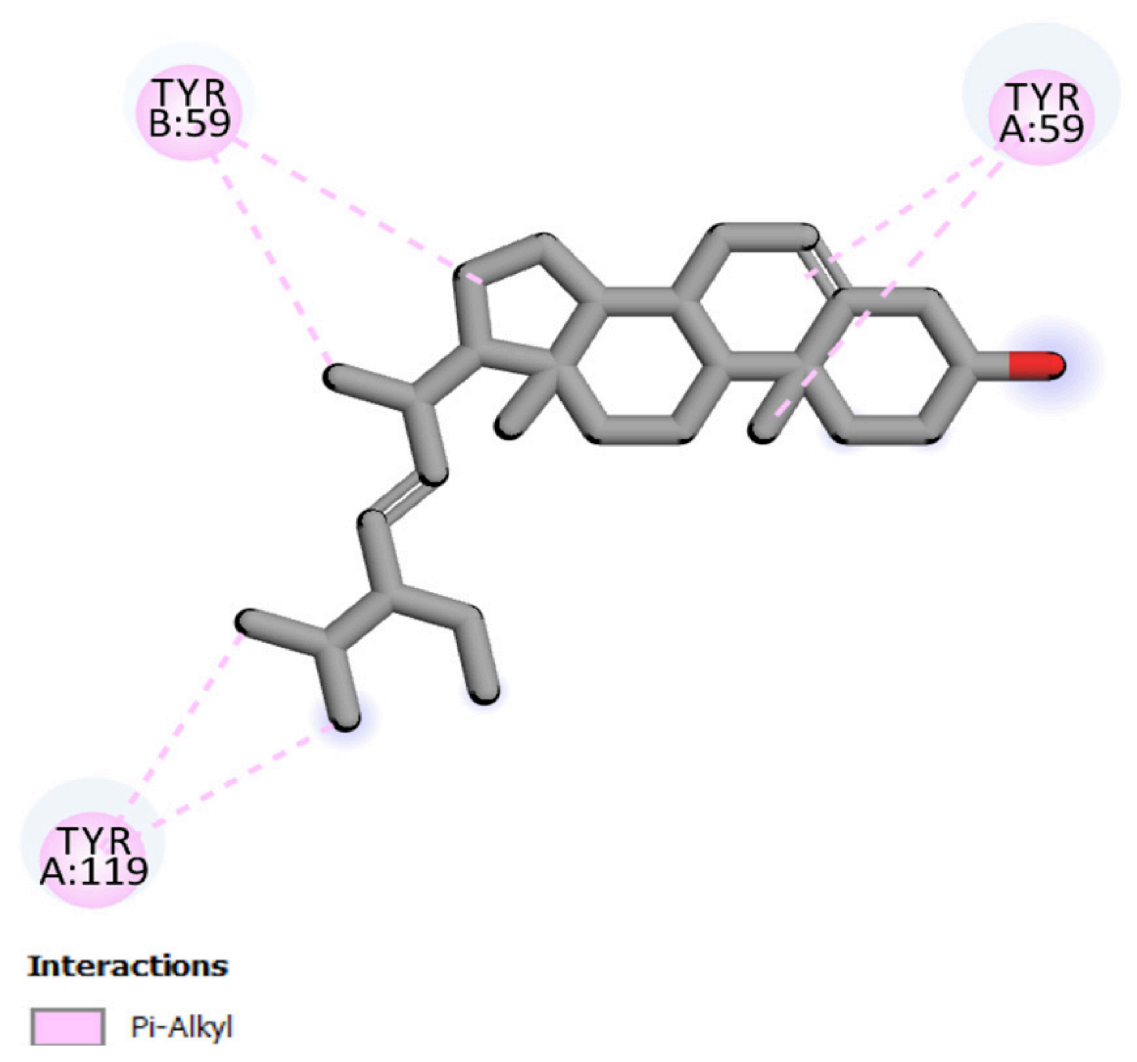	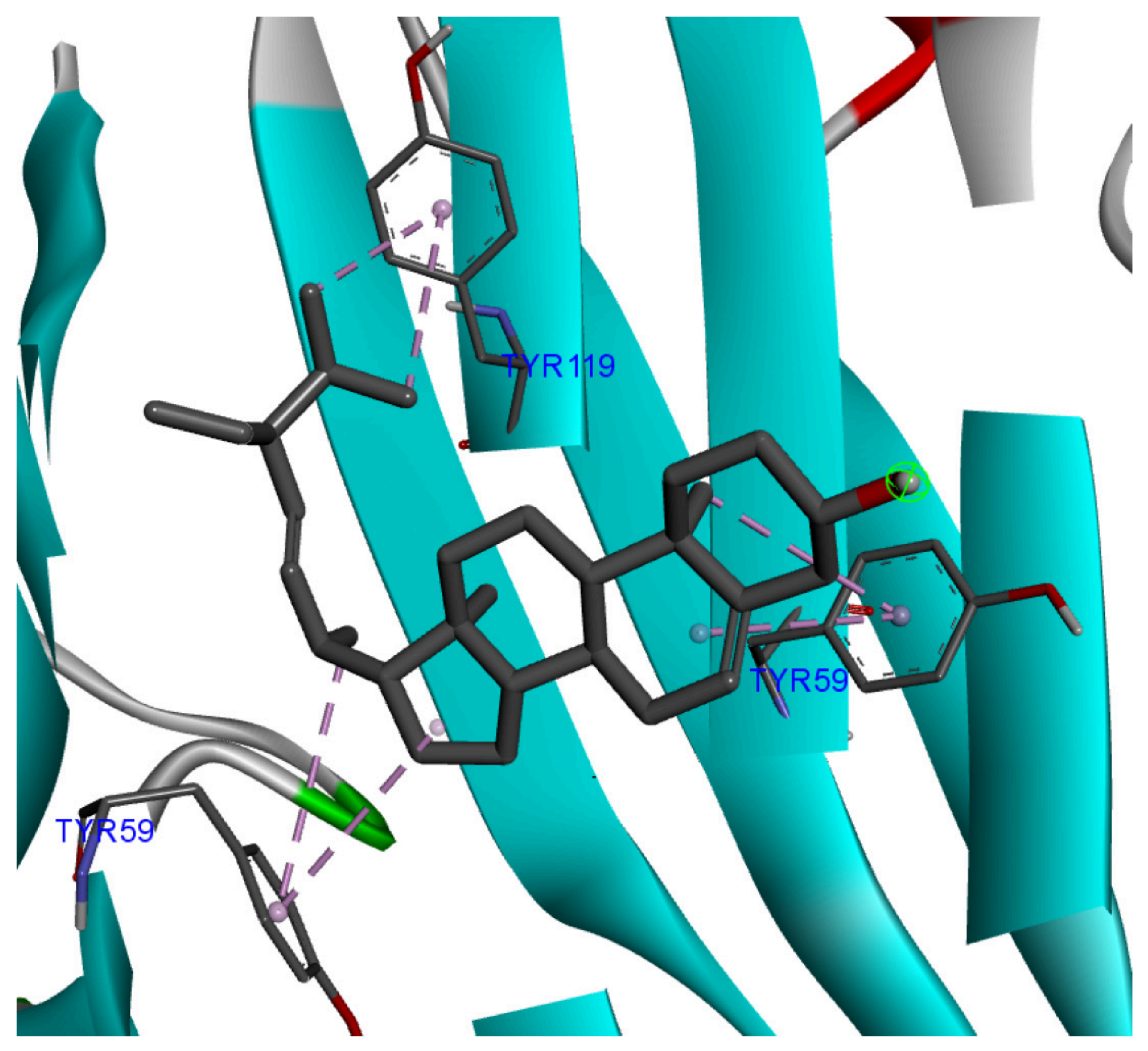
α-Tocopherol	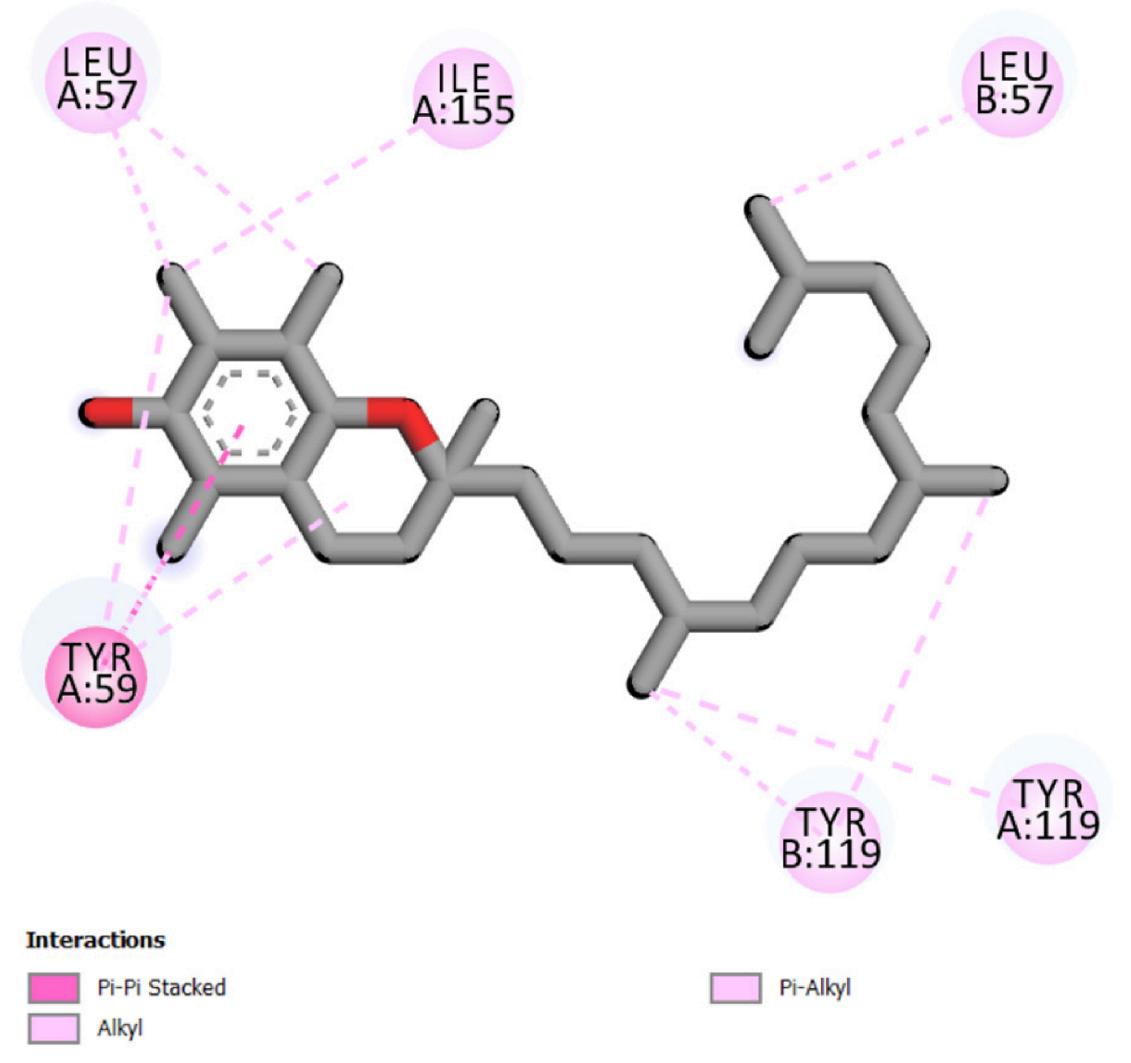	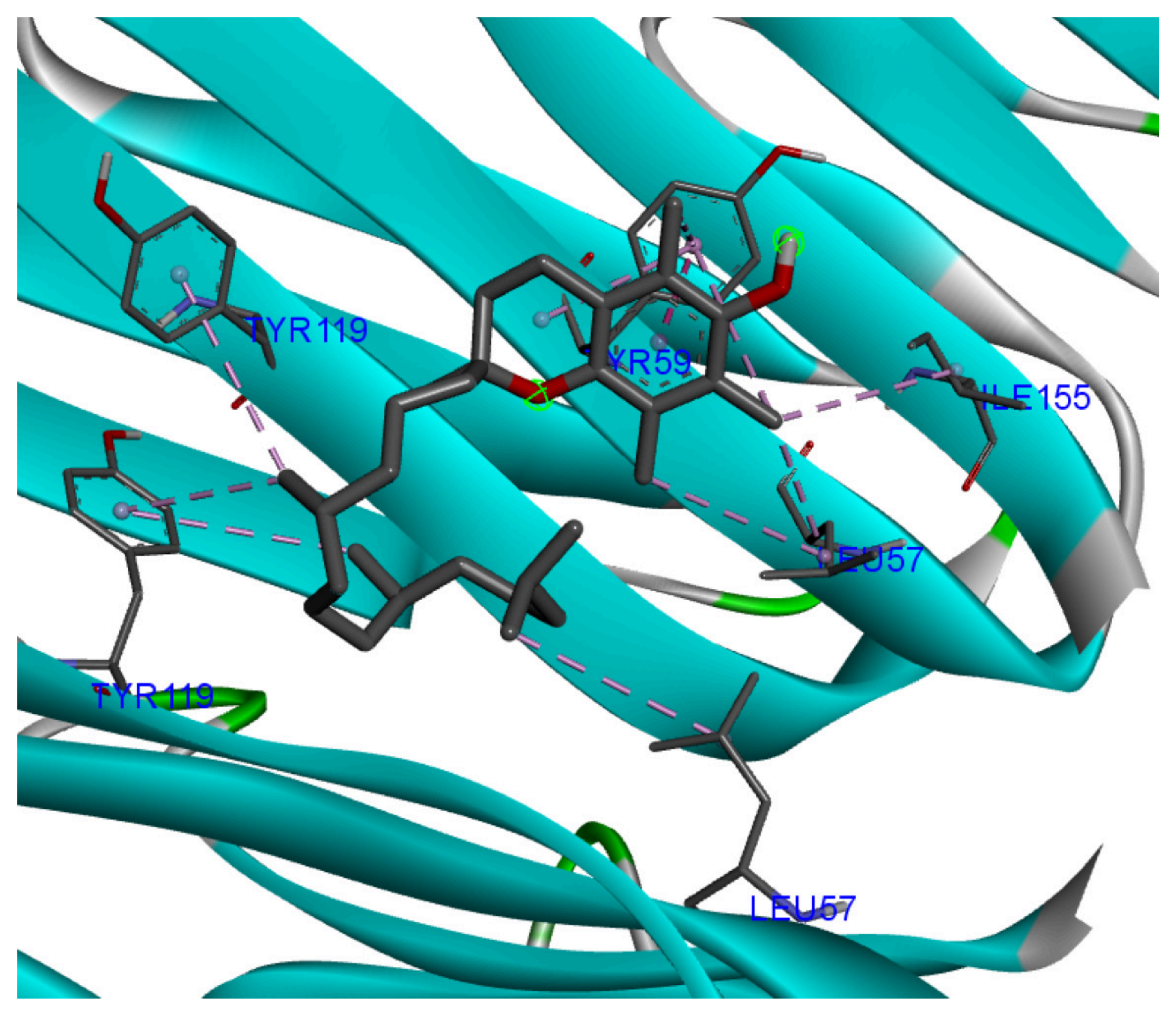
Catechin	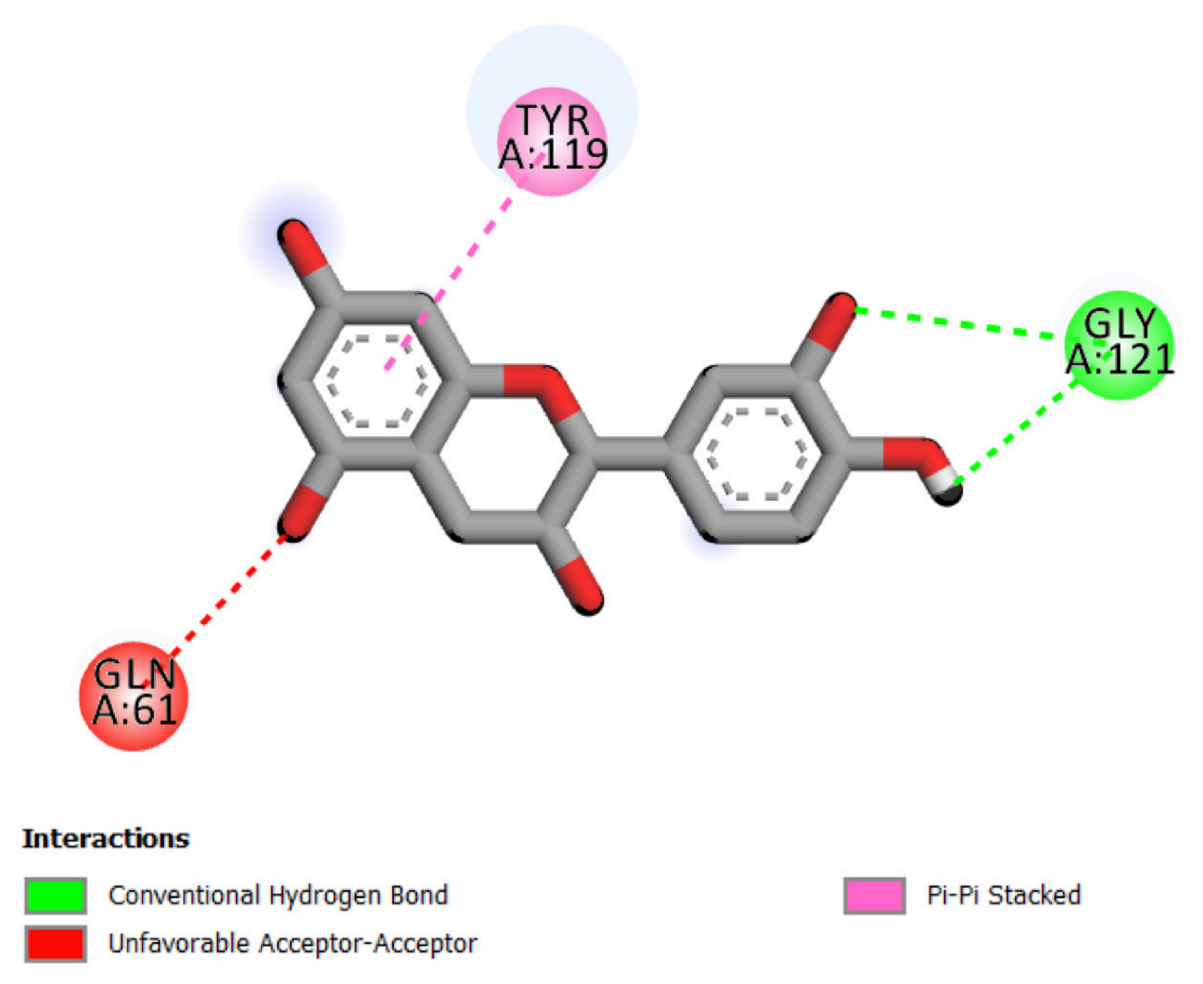	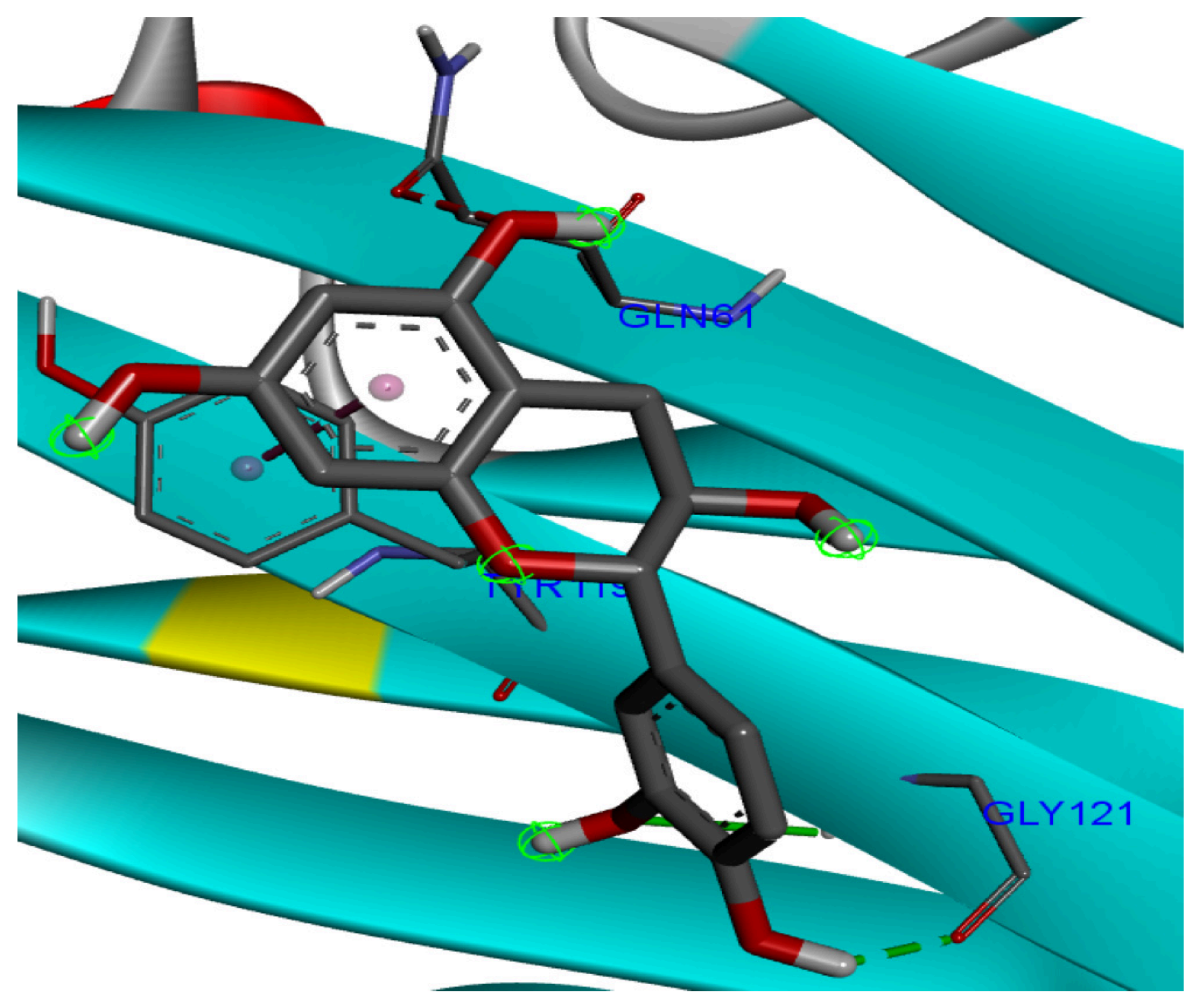
Gallic acid	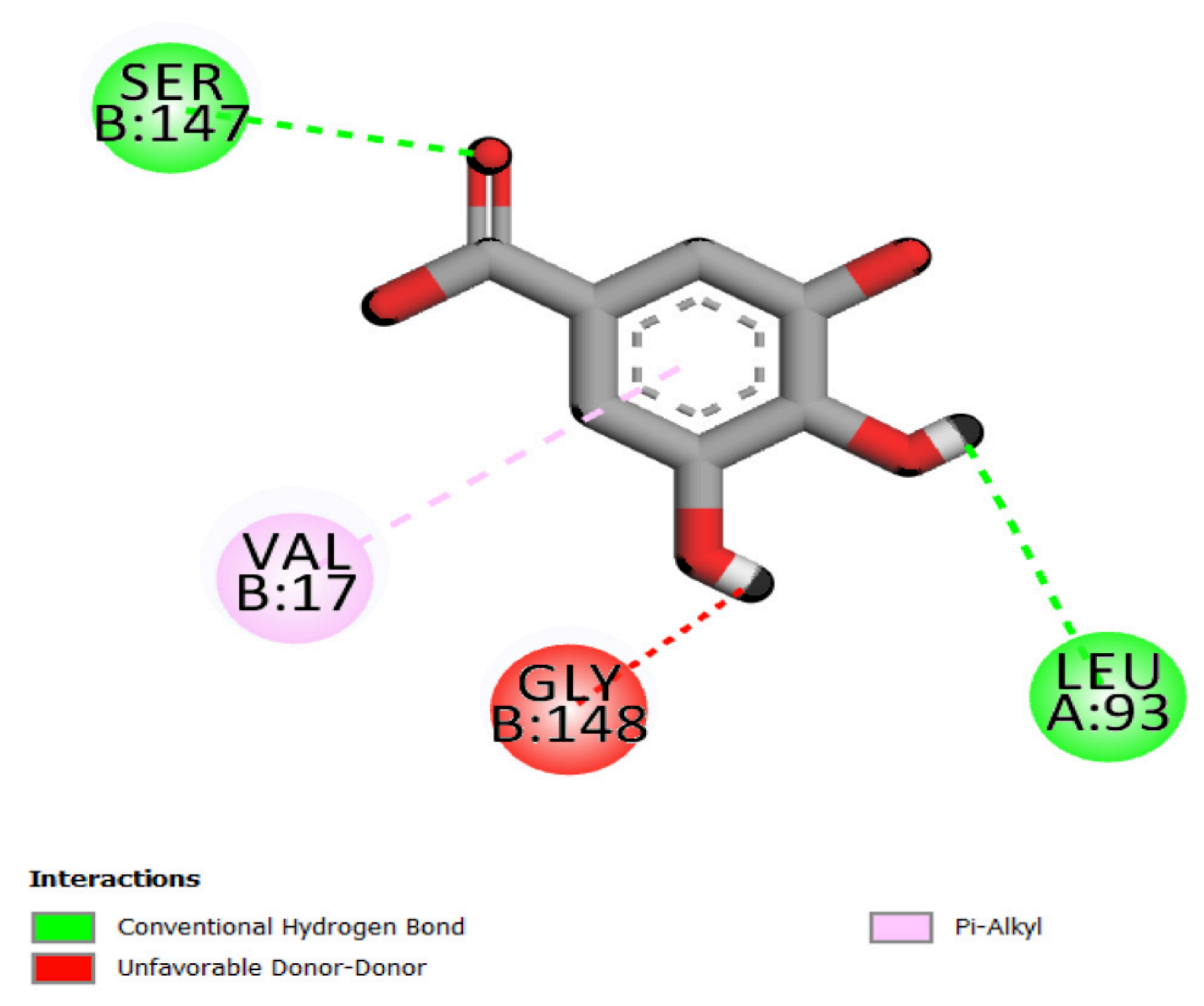	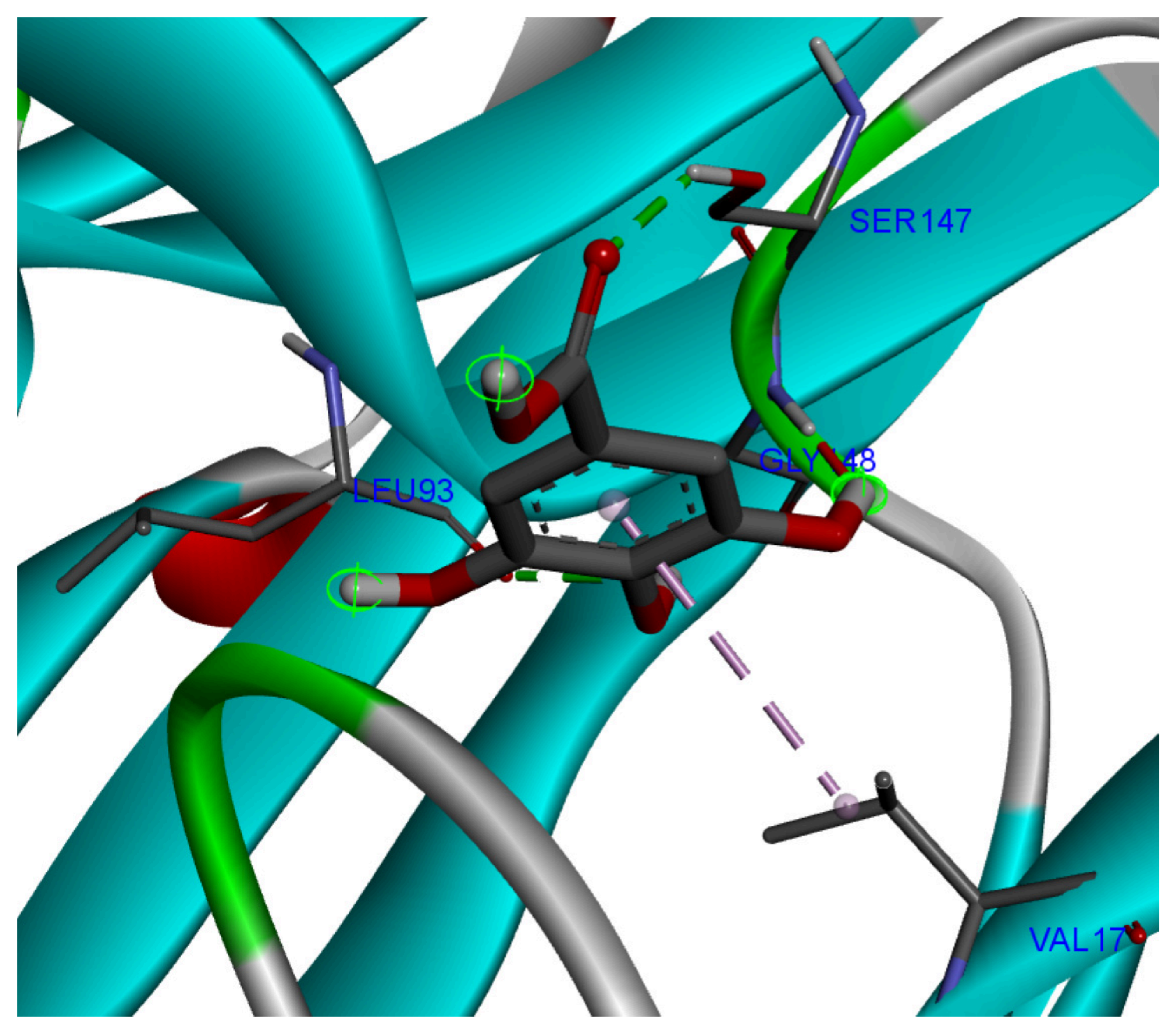

**Table 9:** 2D and 3D interactions between each compound and IL-6.

Ligand	IL-6	
	2D	3D
β-Sitosterol	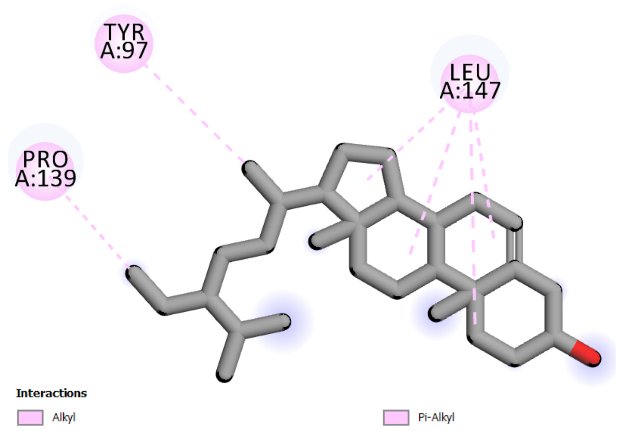	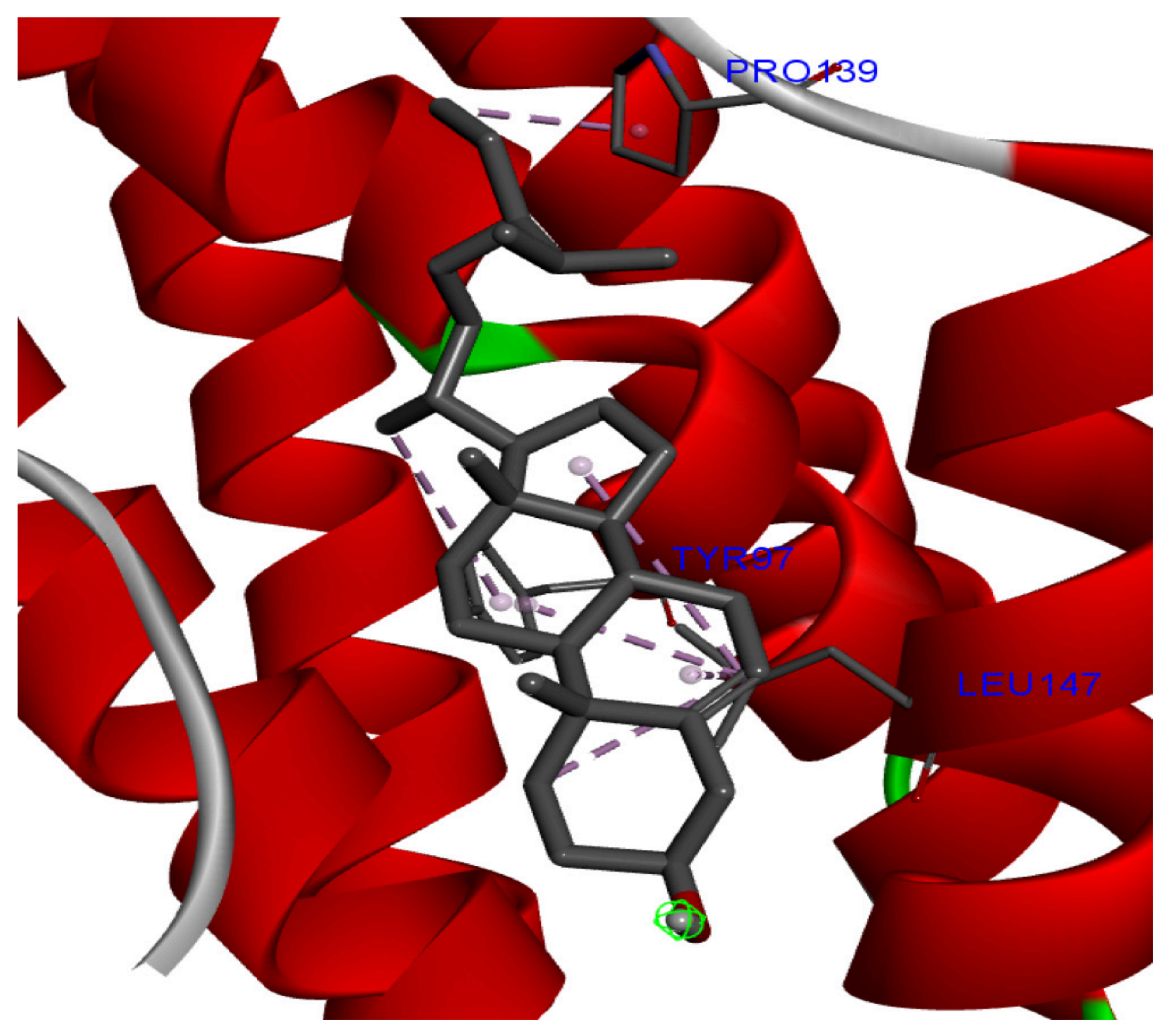
Campesterol	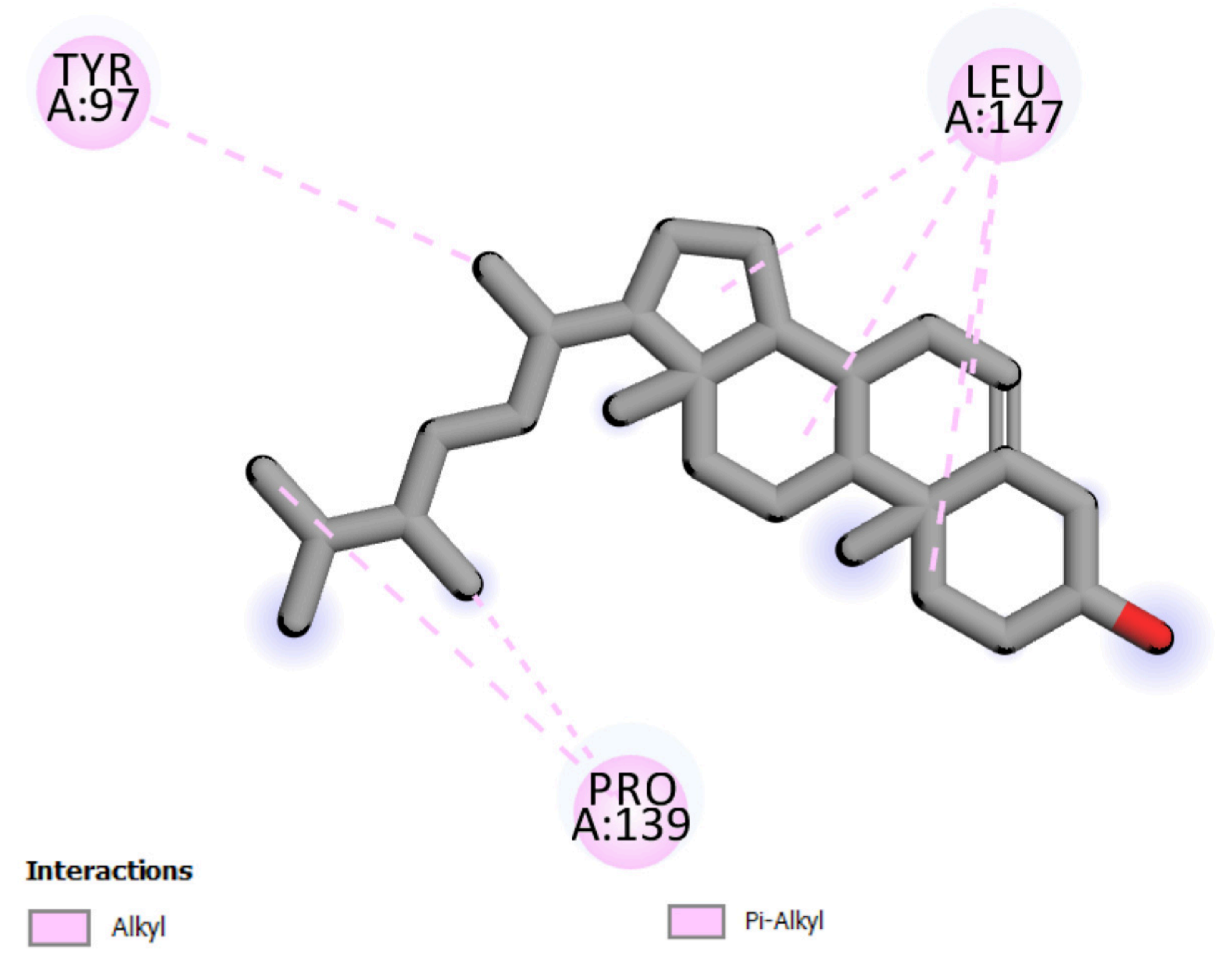	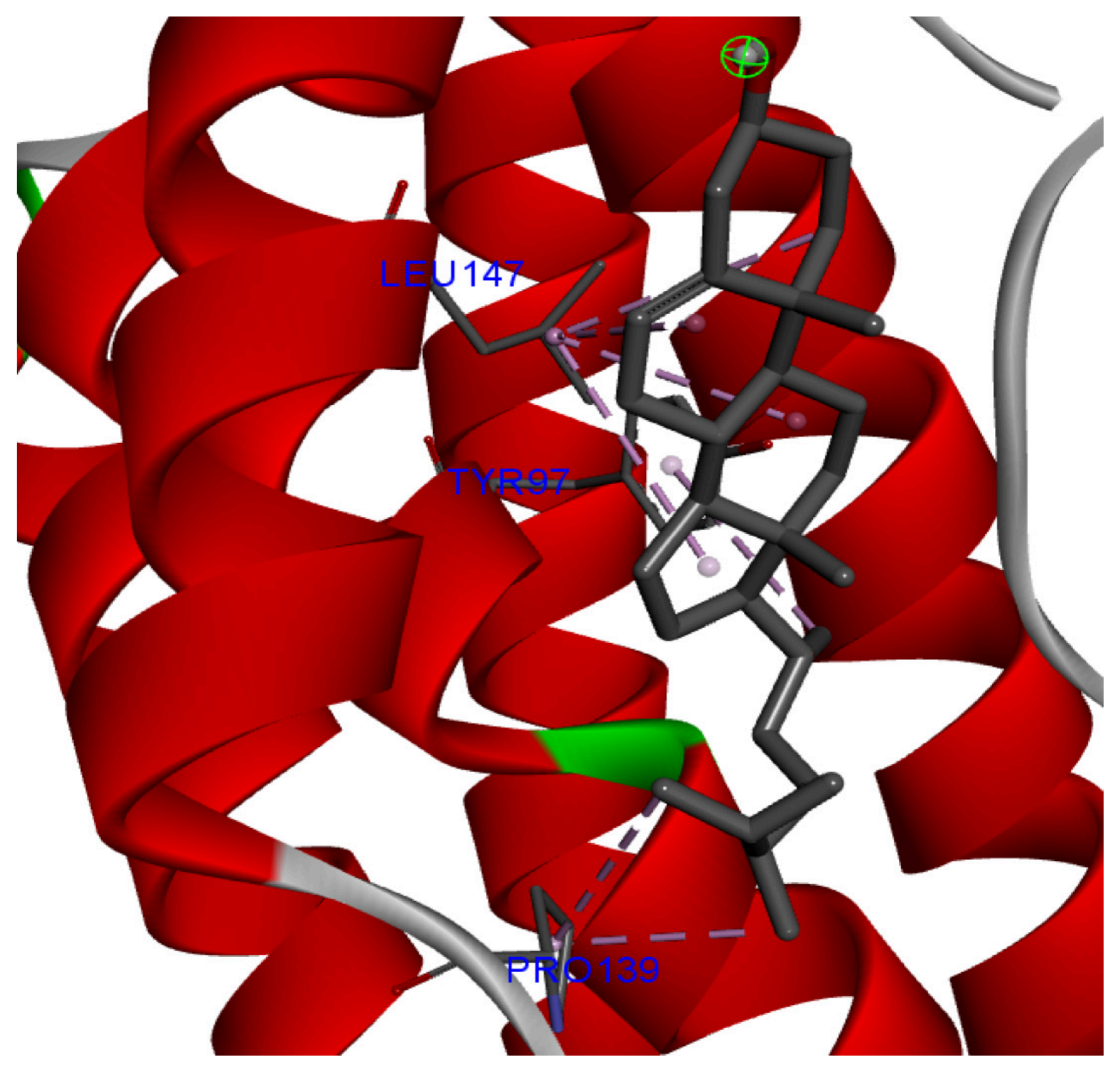
Stigmasterol	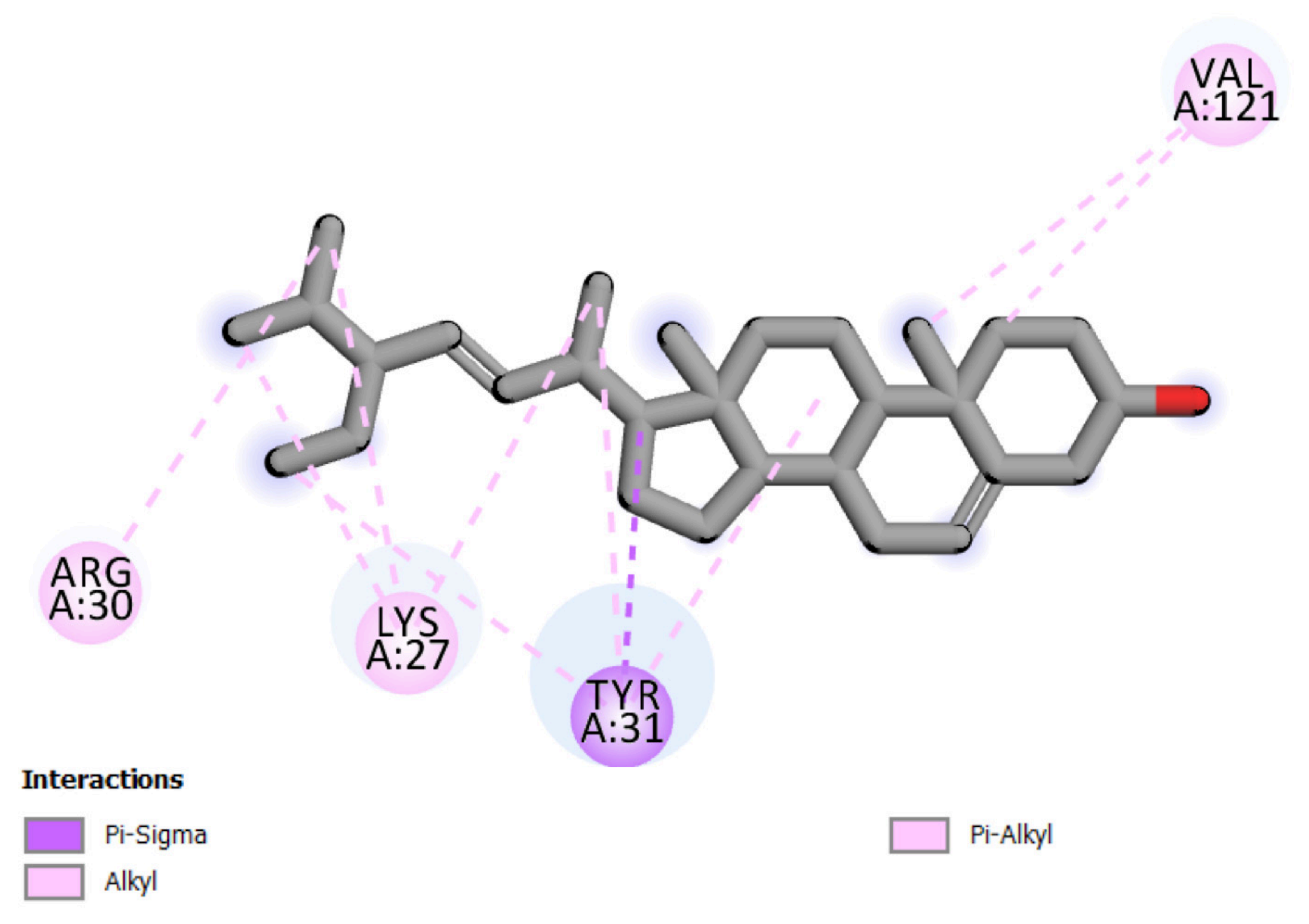	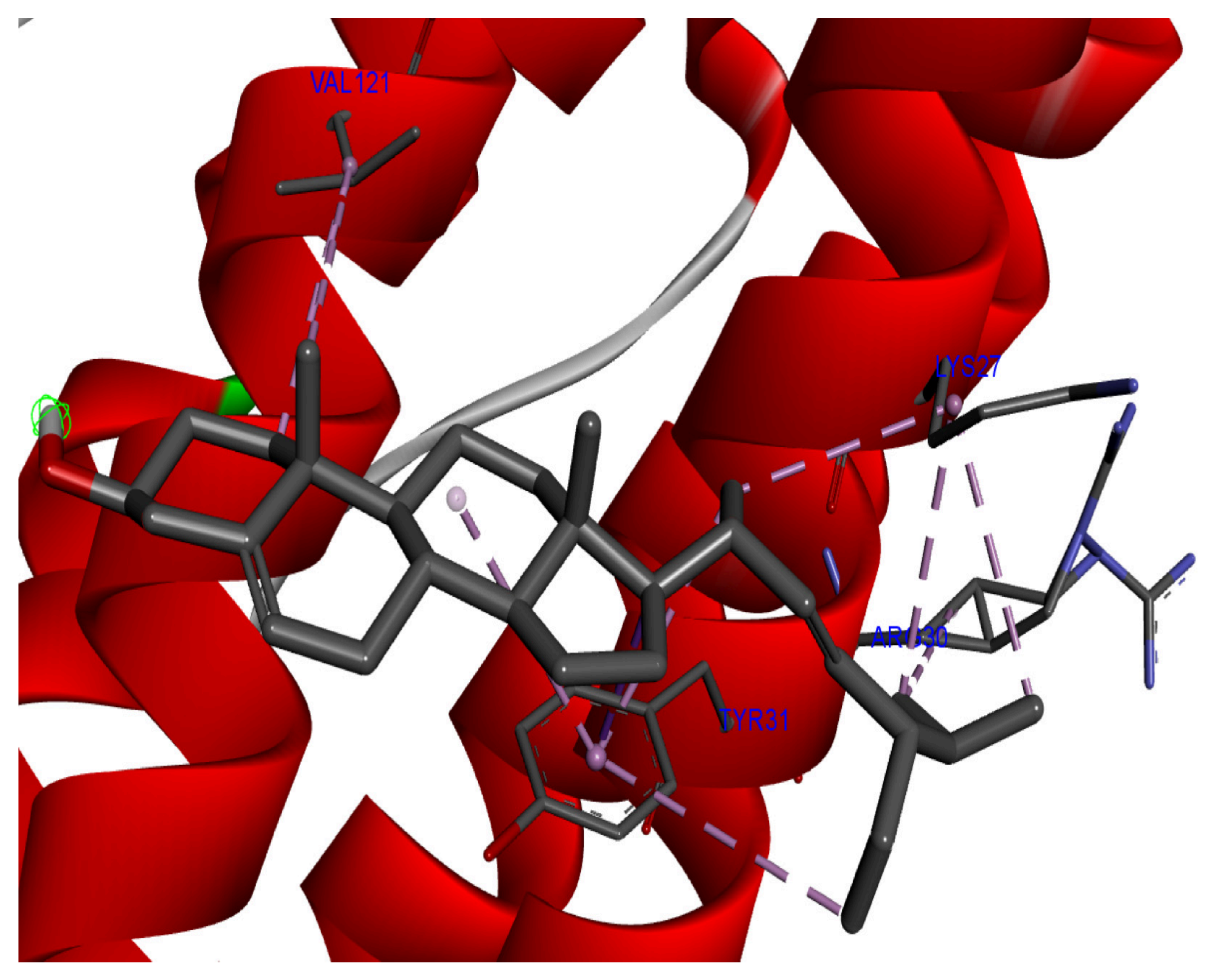
α-Tocopherol	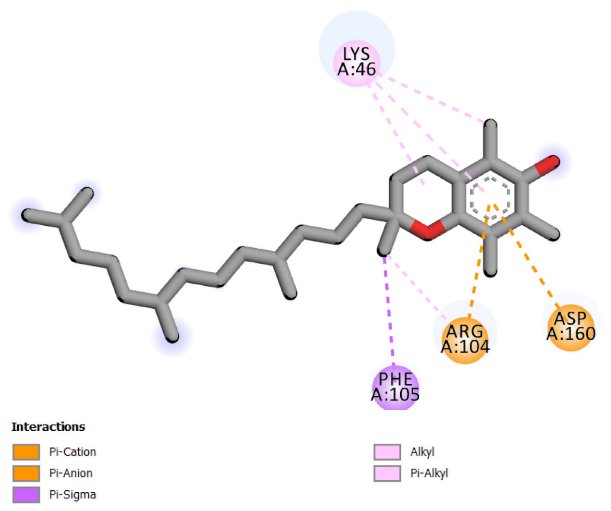	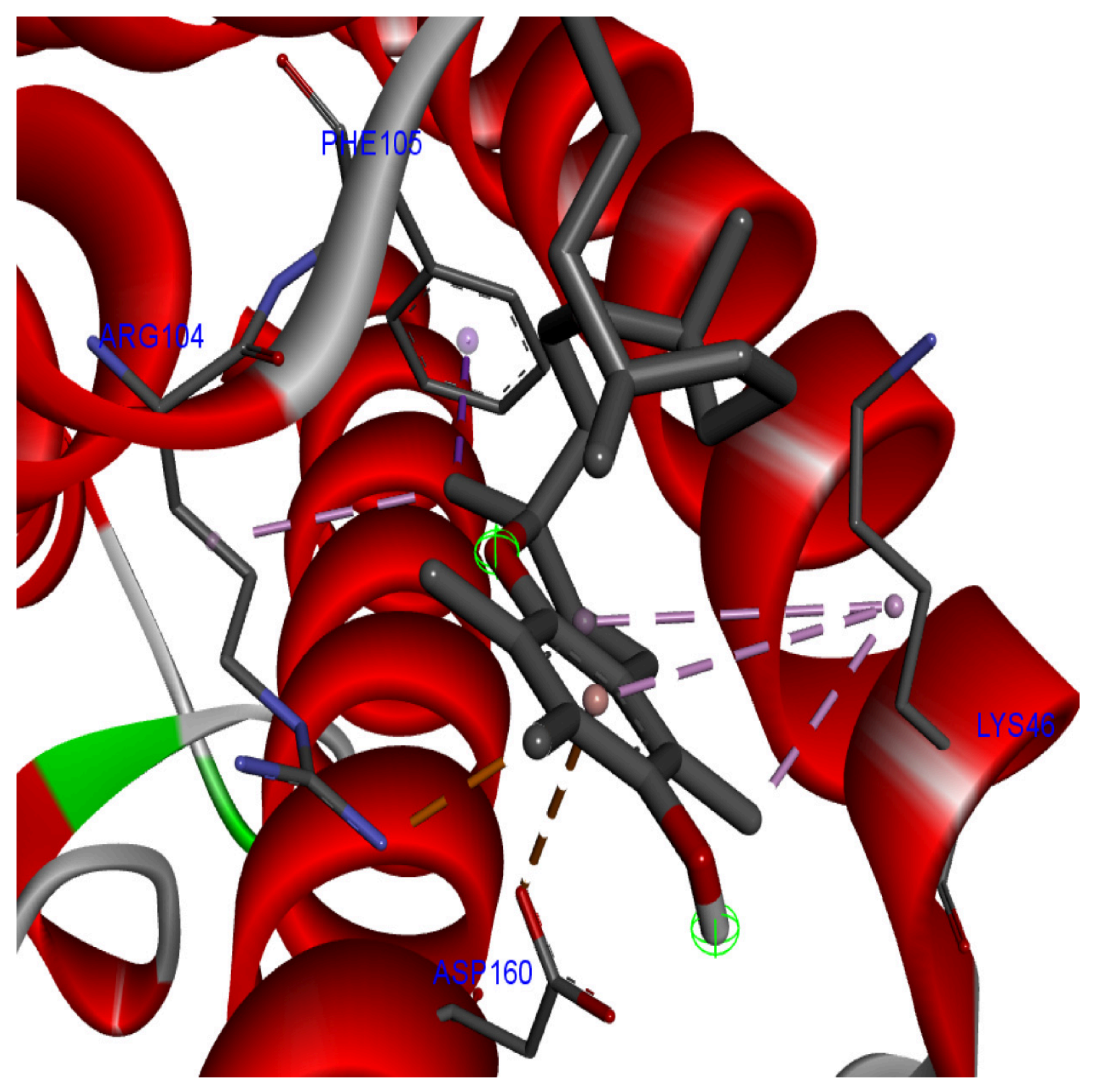
Catechin	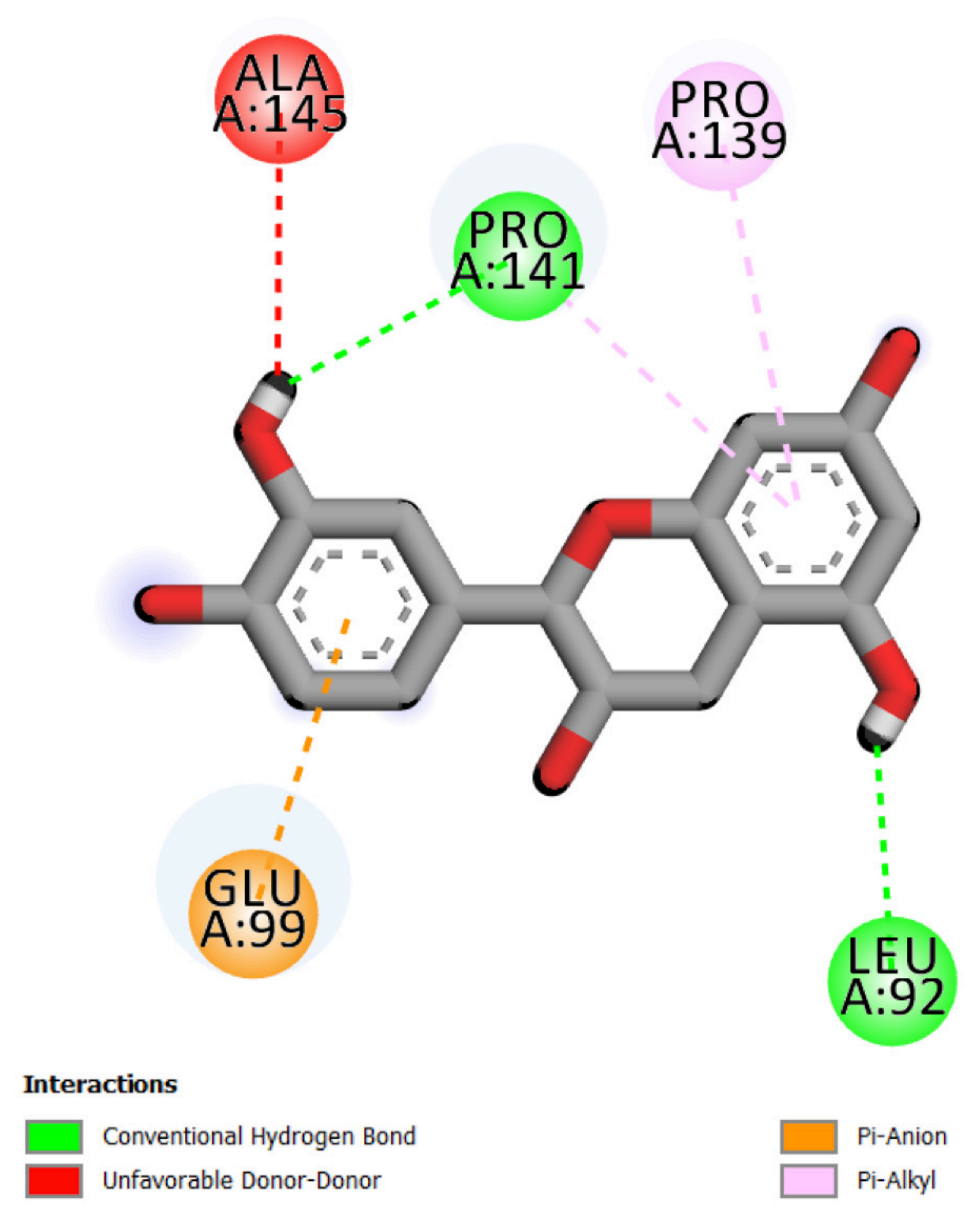	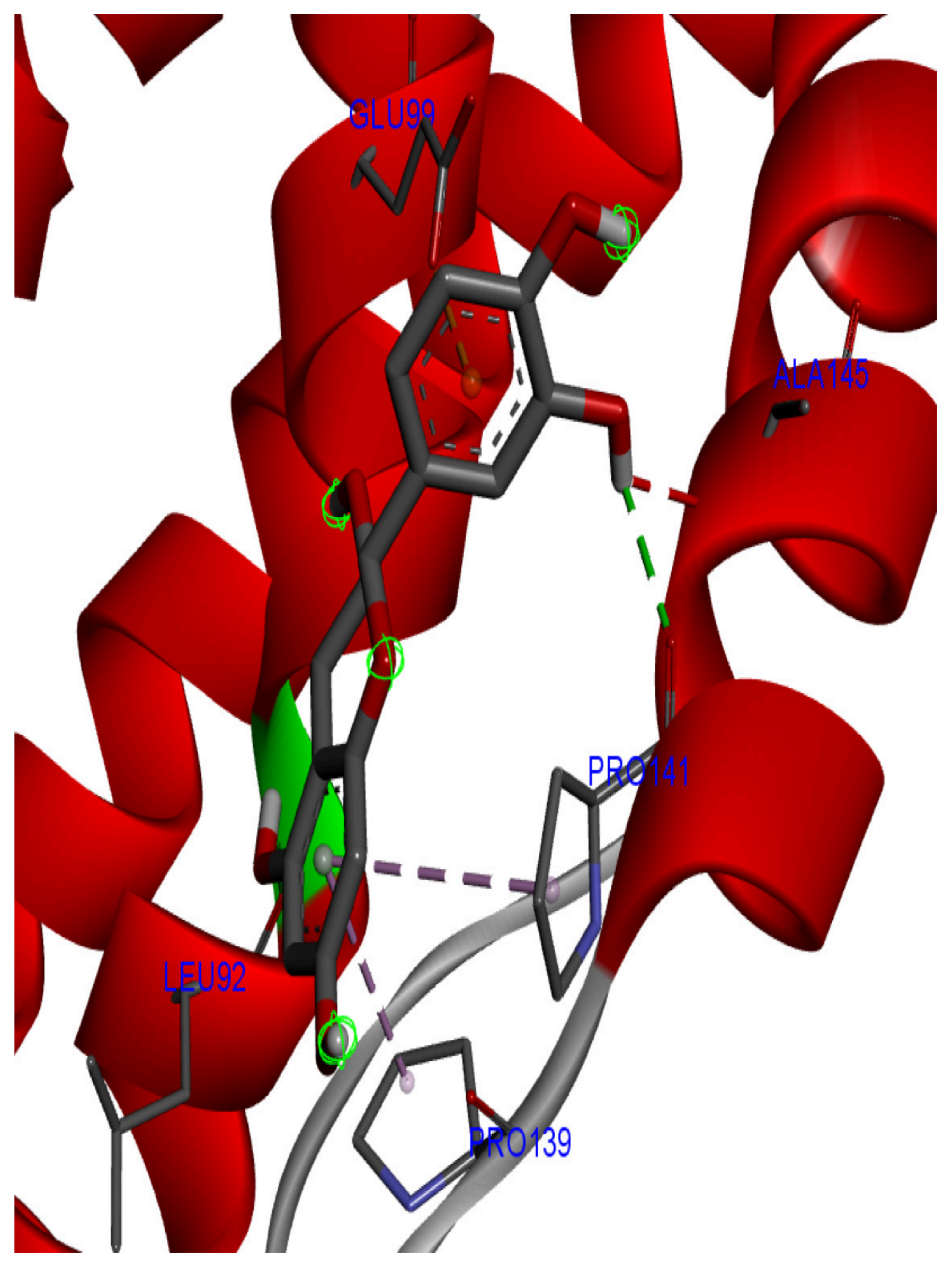
Gallic acid	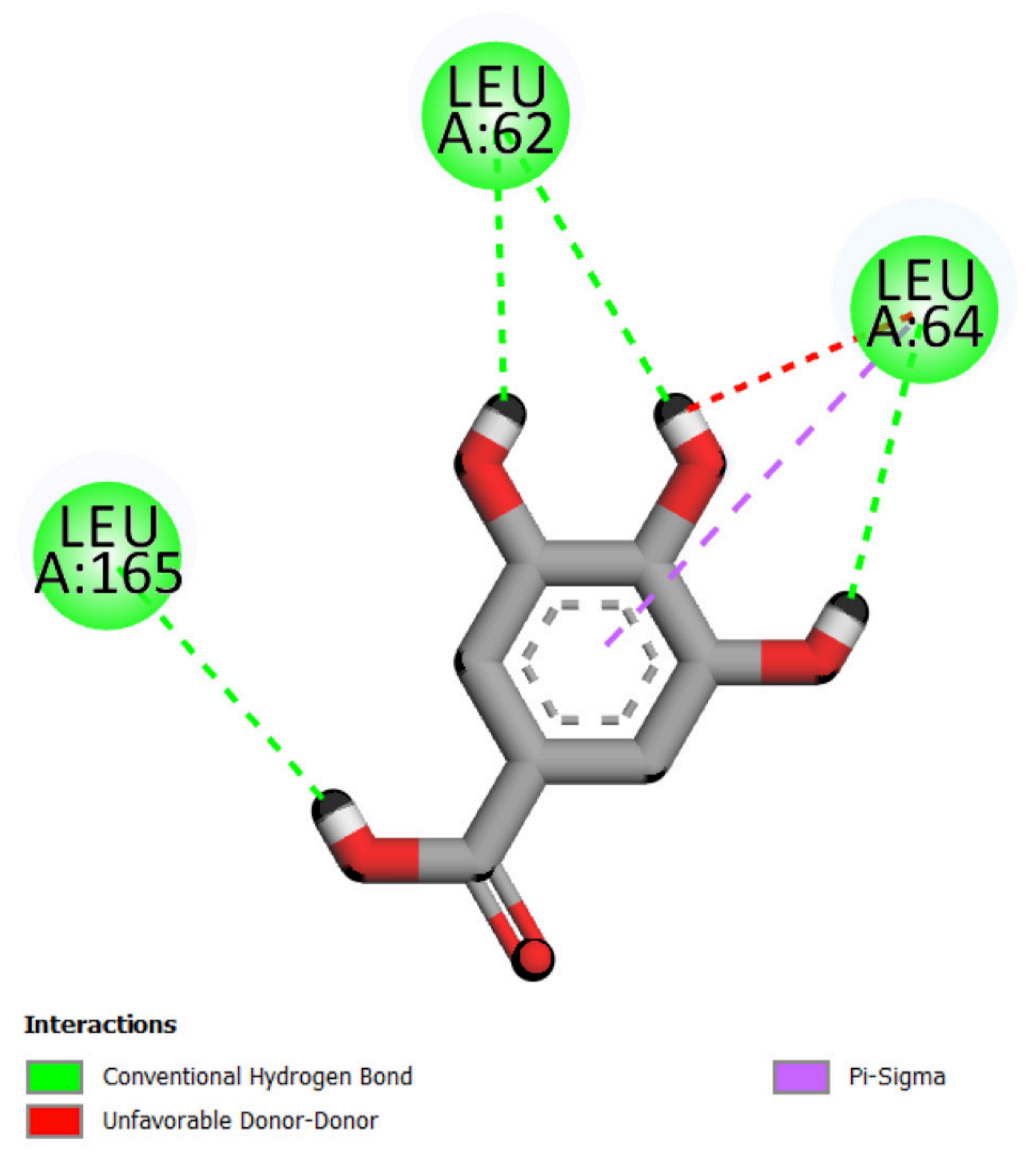	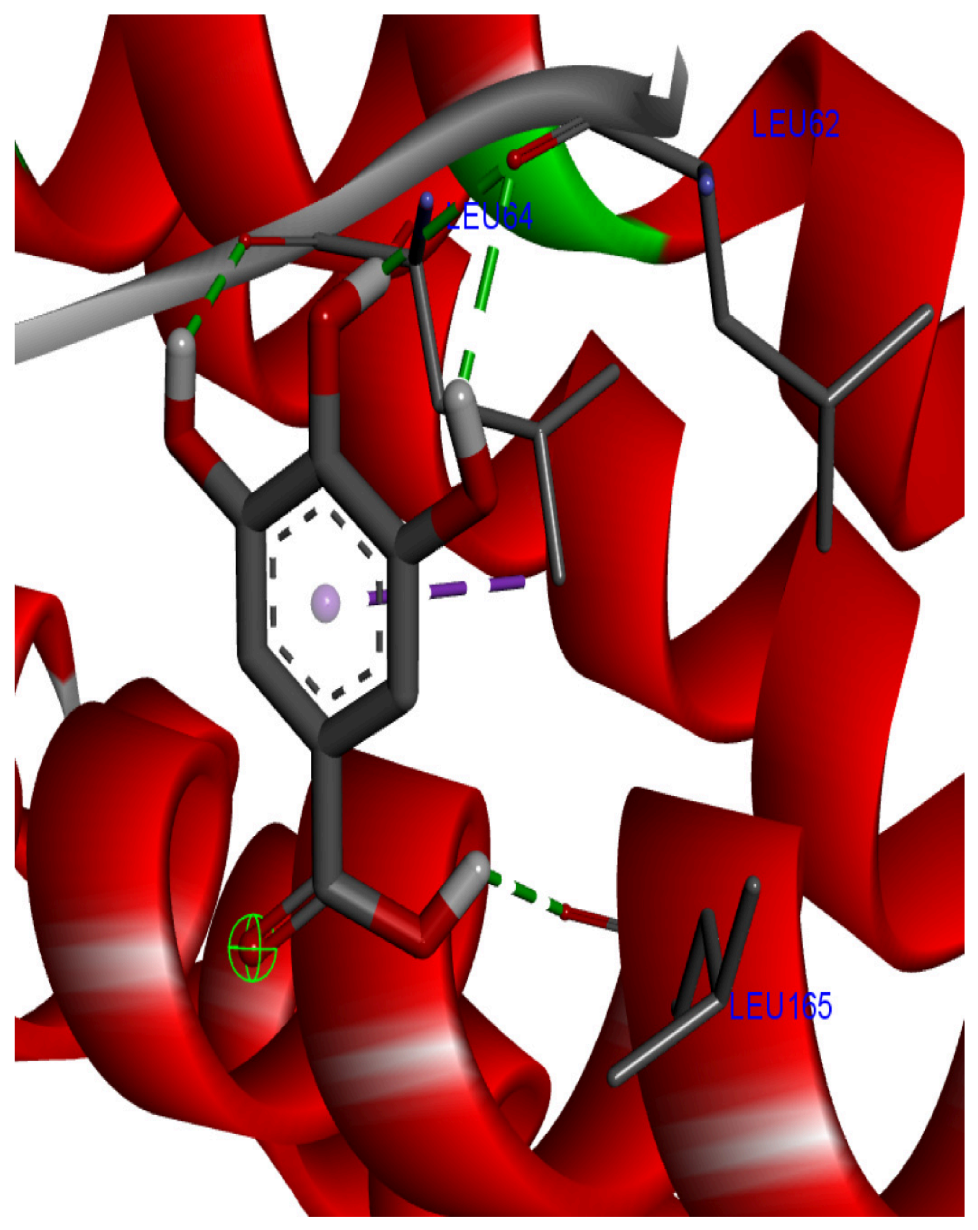

**Table 10:** 2D and 3D interactions between each compound and AChE.

Ligand	AChE	
	2D	3D
β-Sitosterol	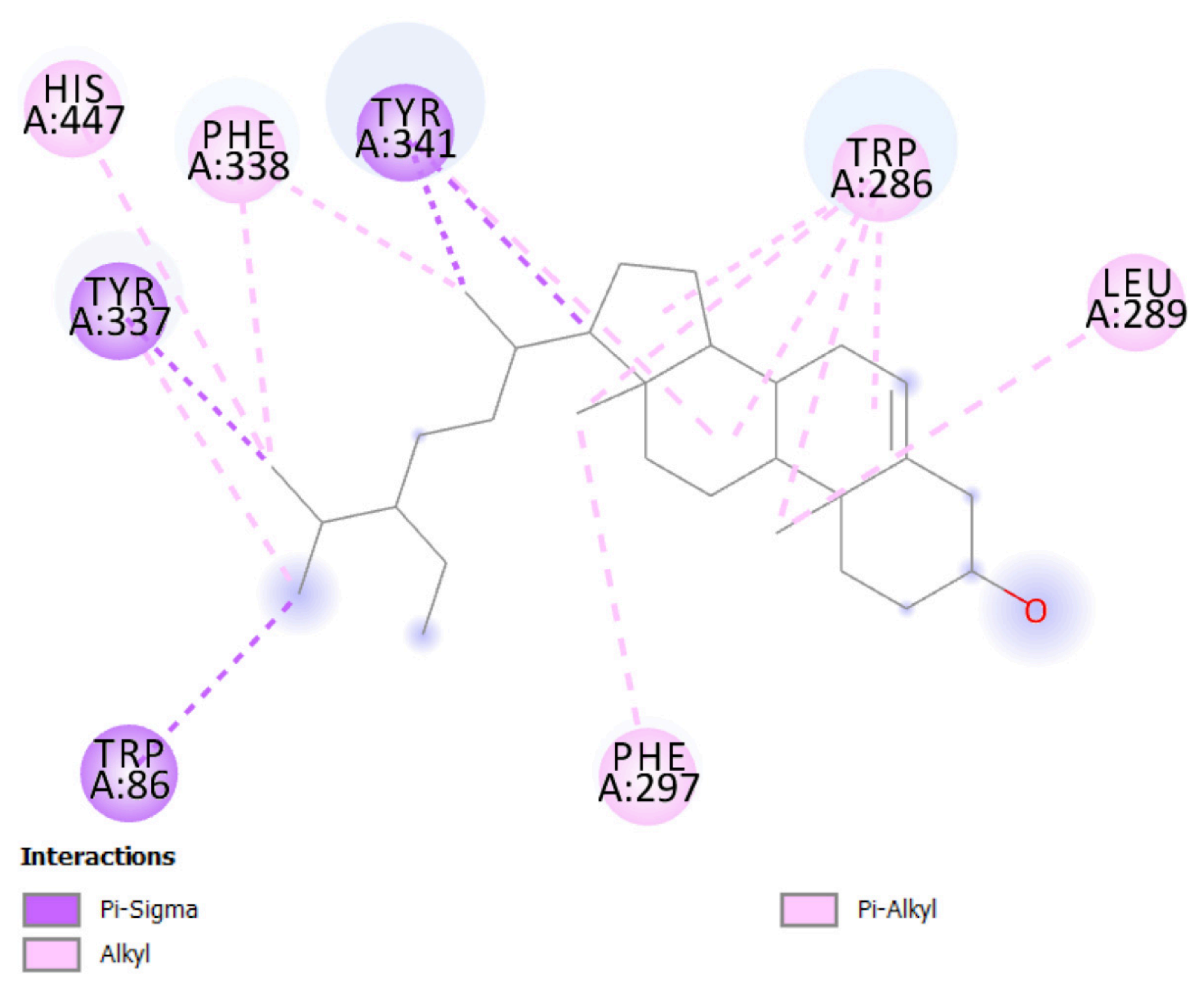	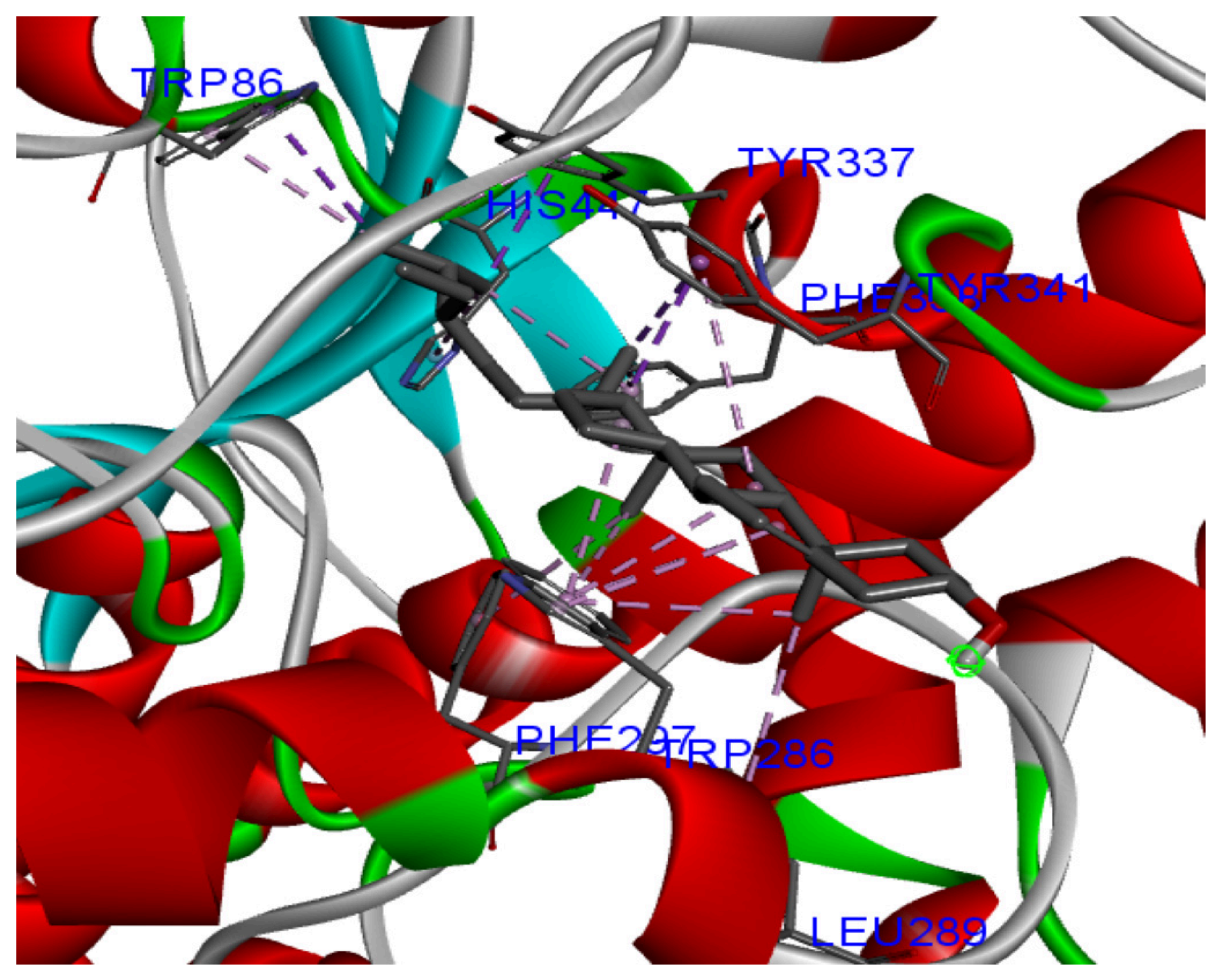
Campesterol	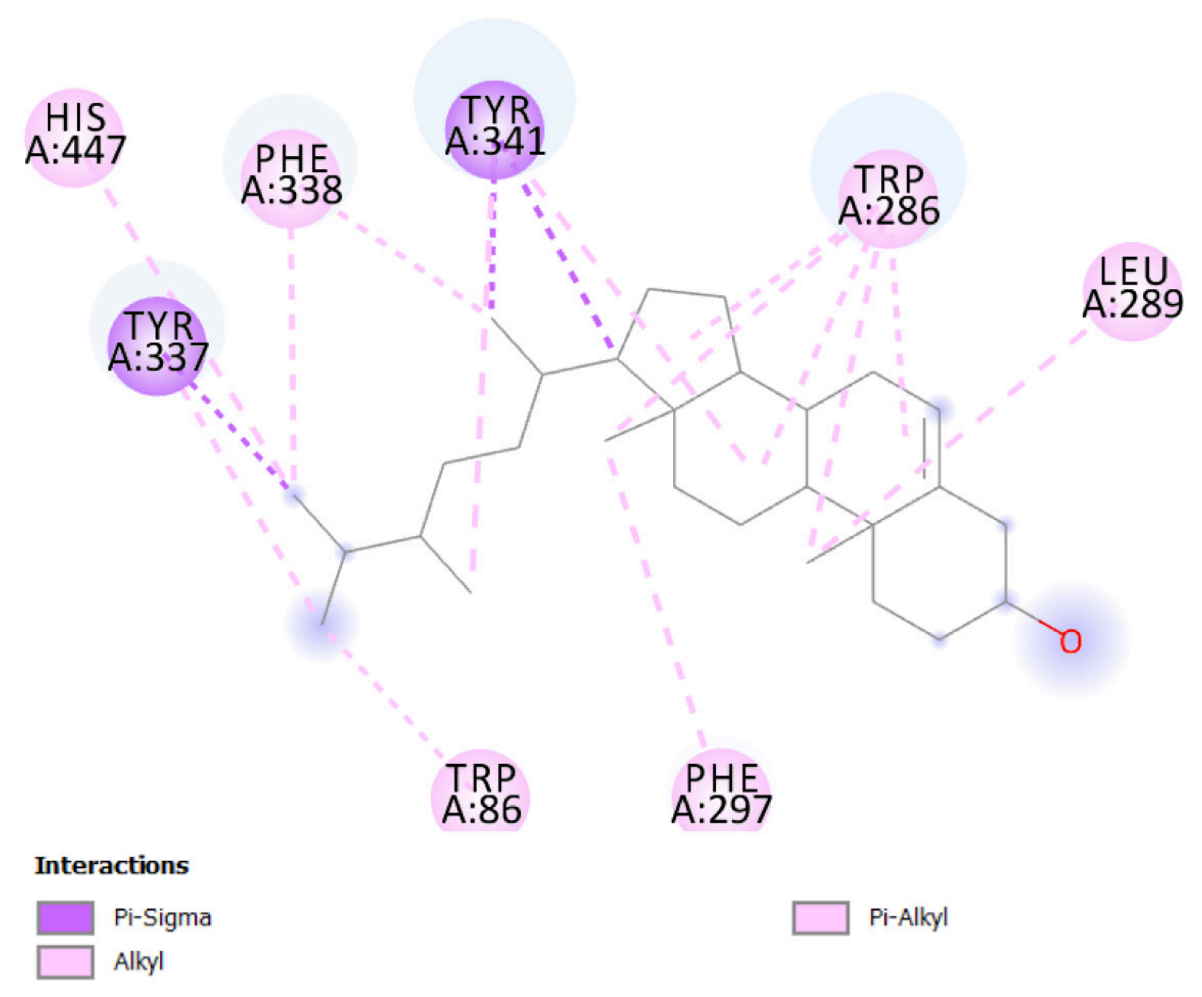	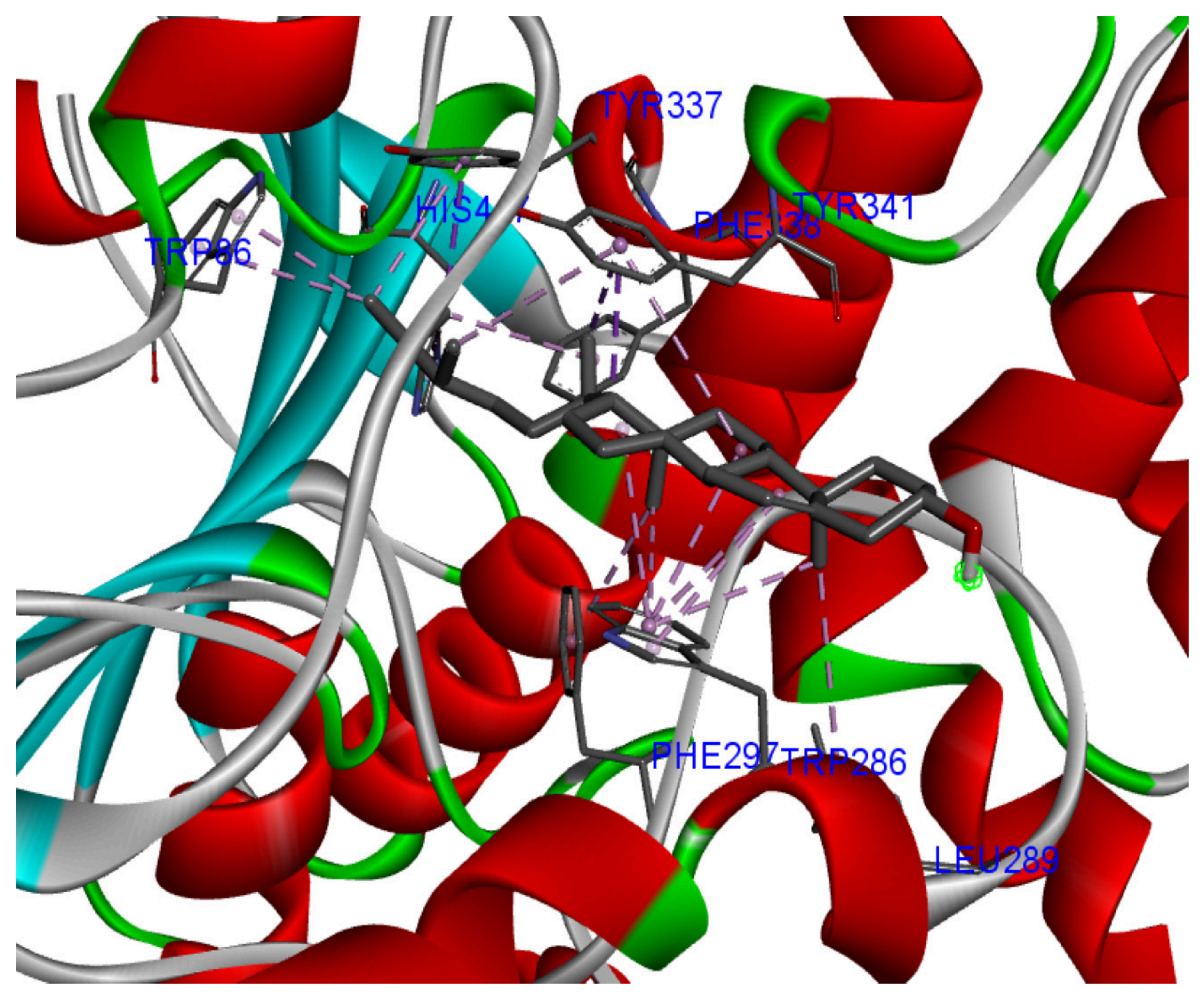
Stigmasterol	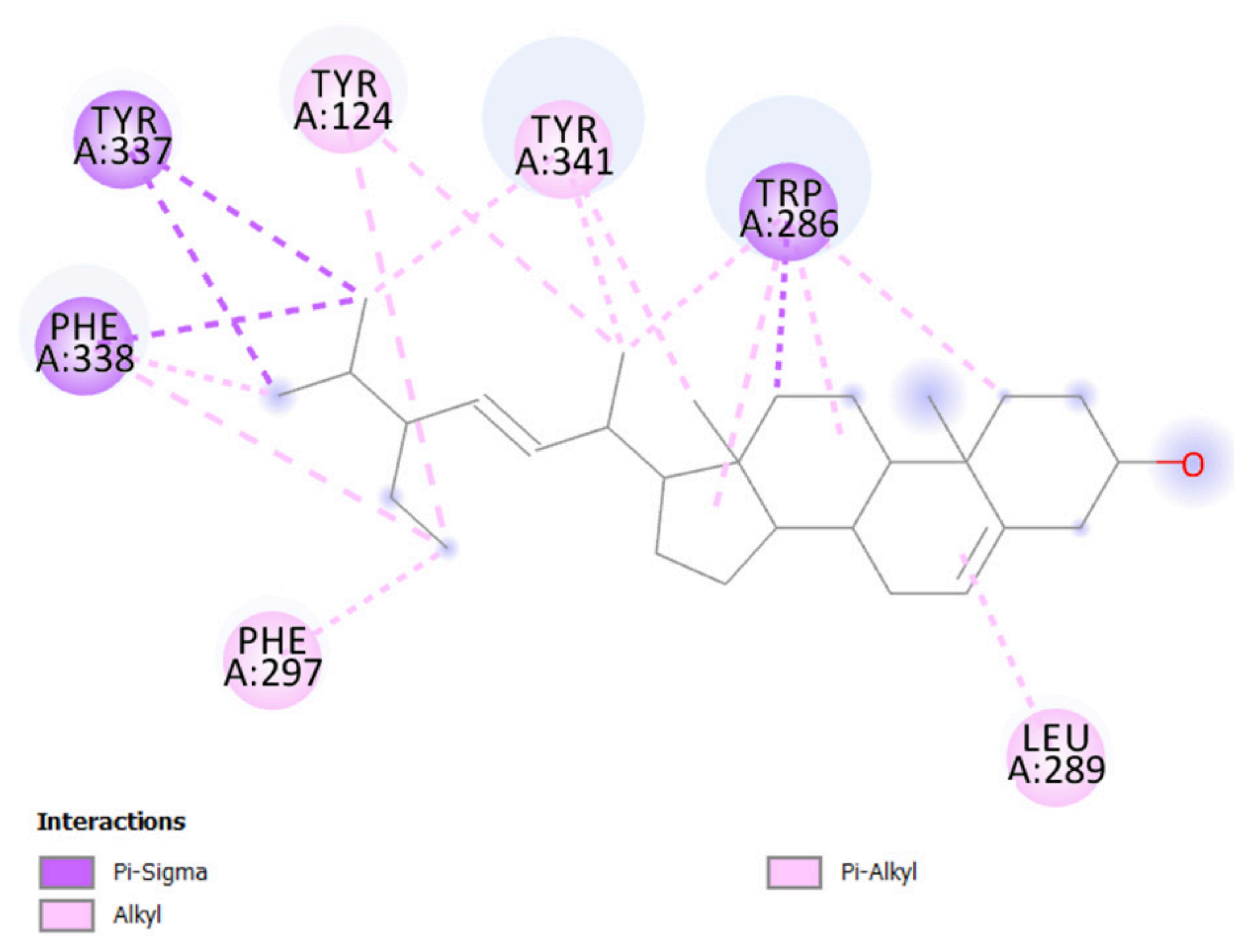	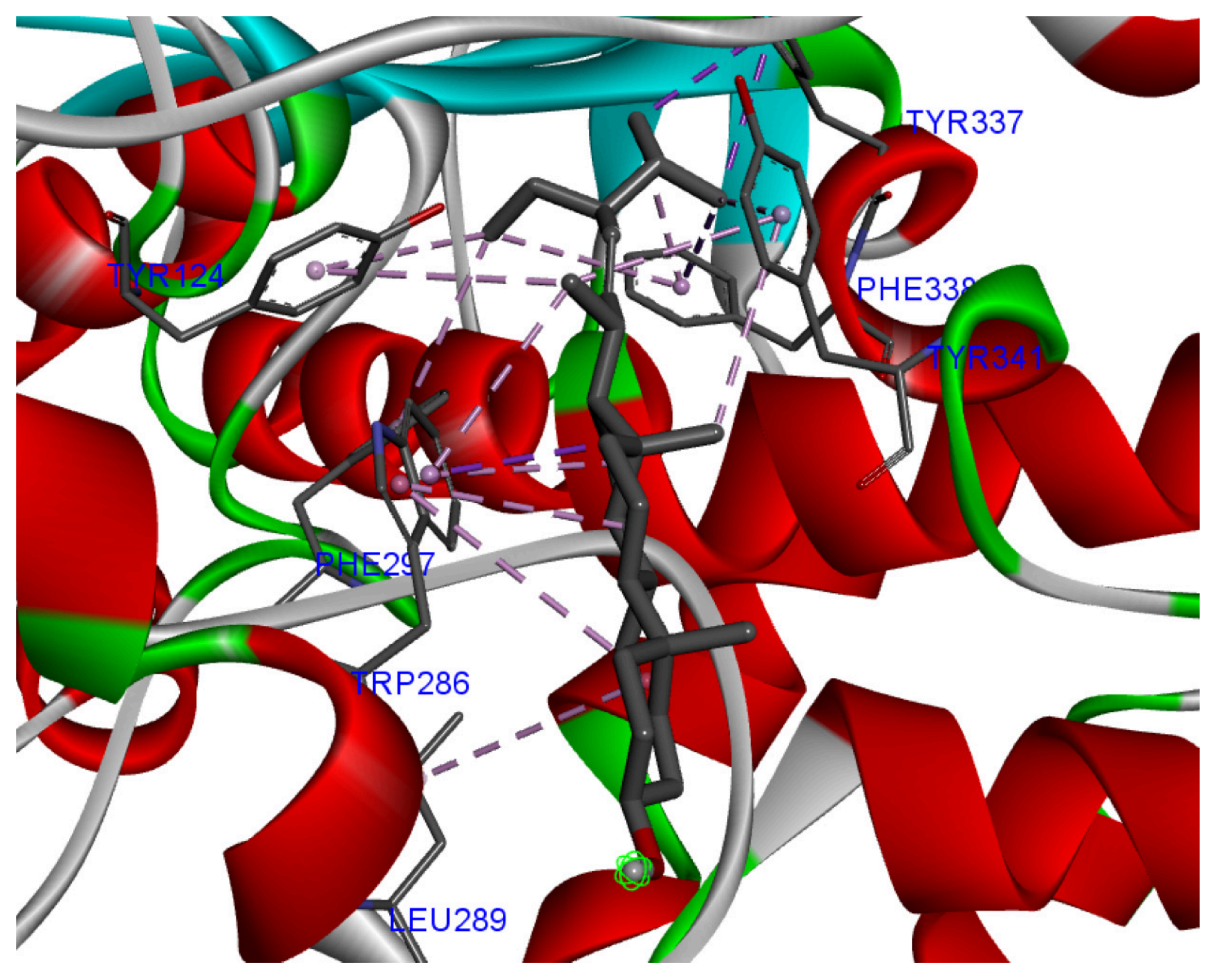
α-Tocopherol	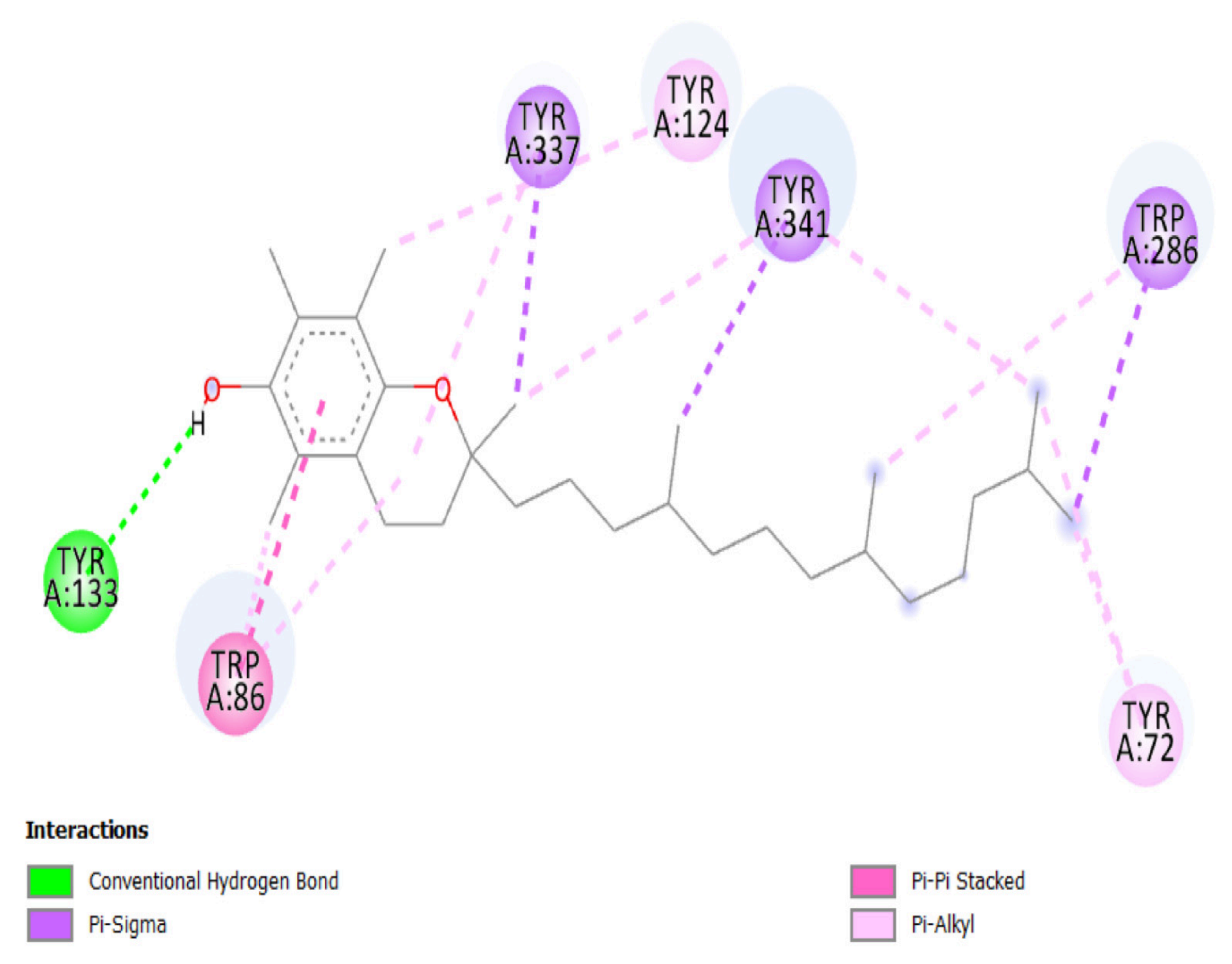	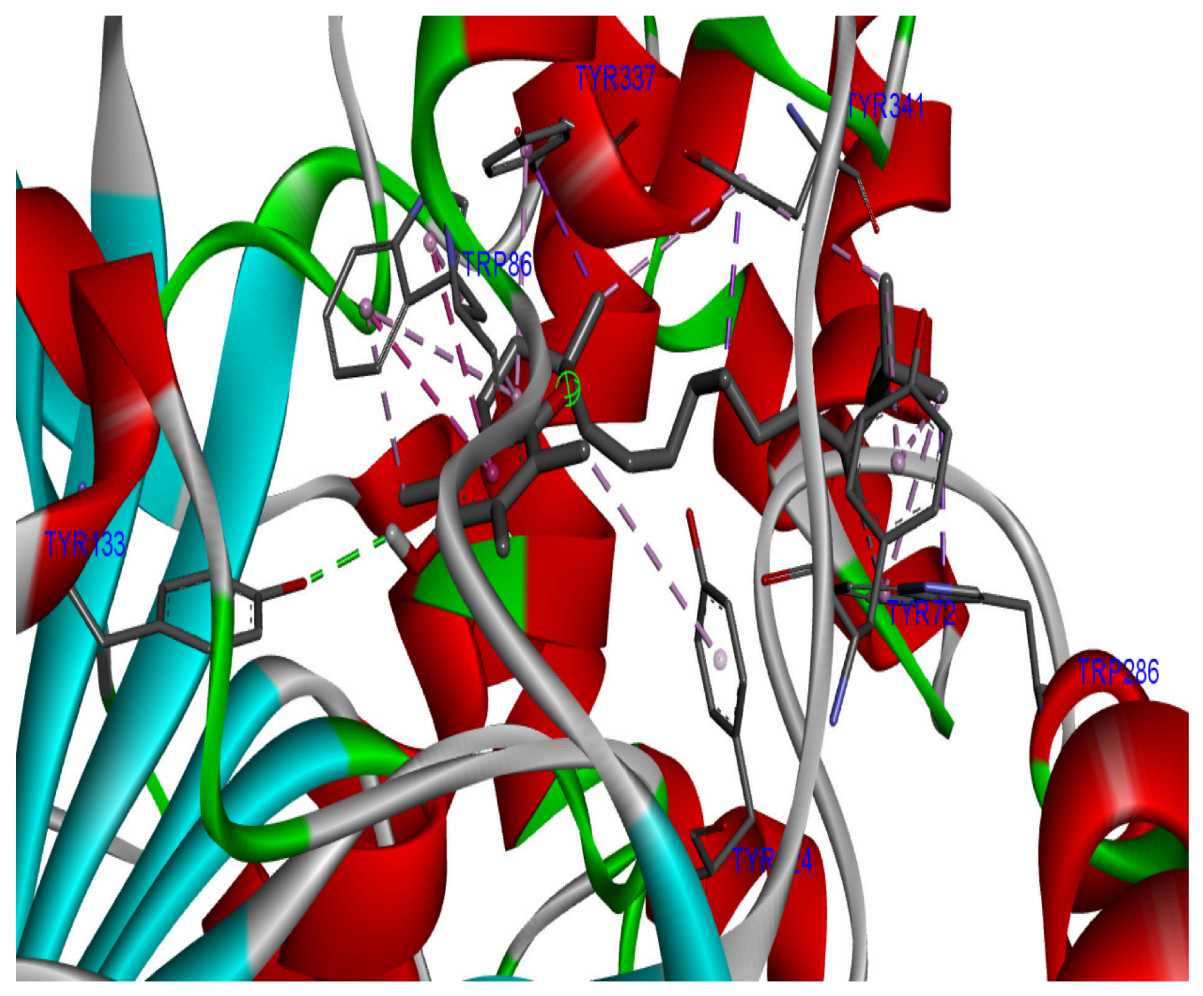
Catechin	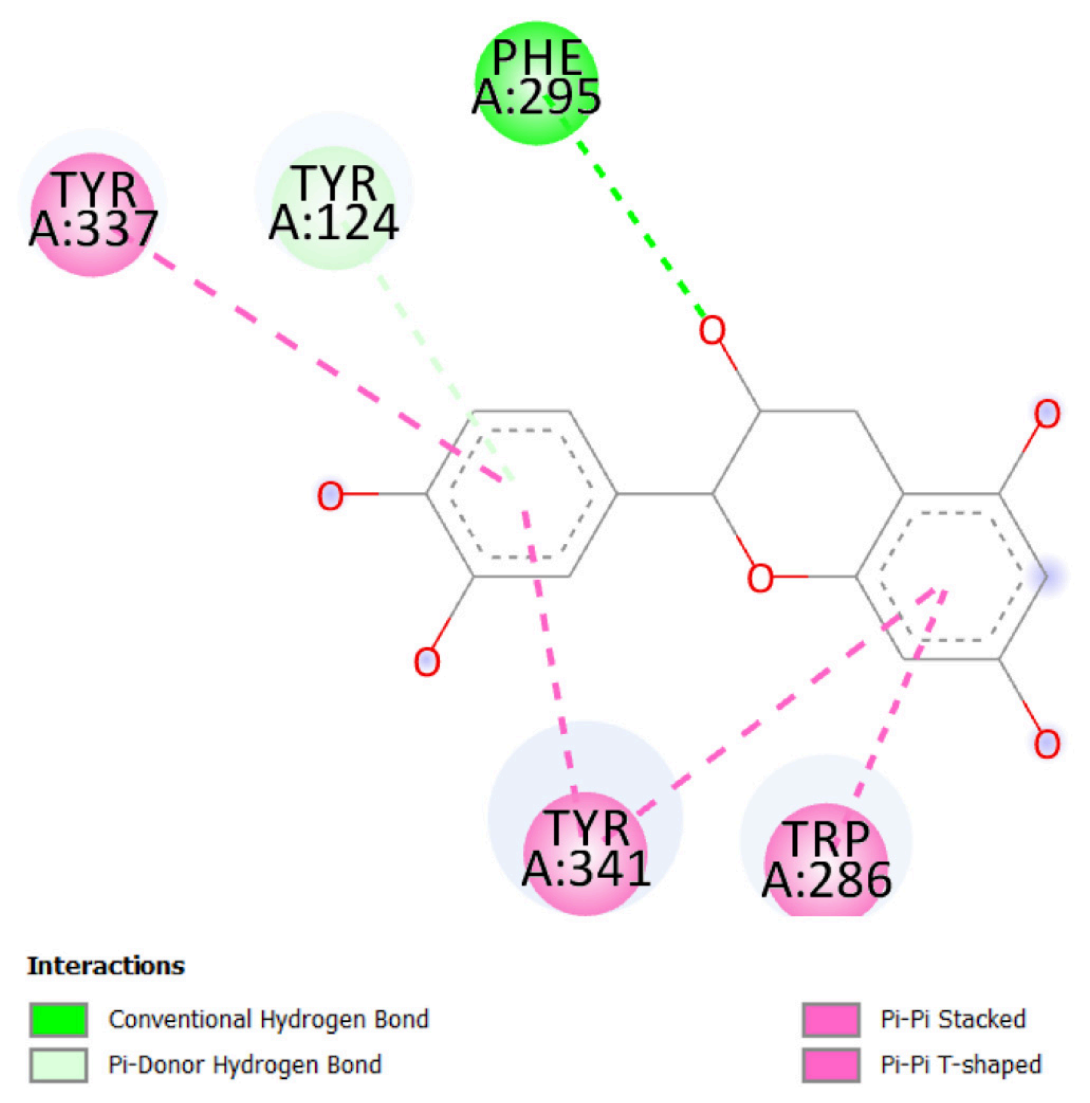	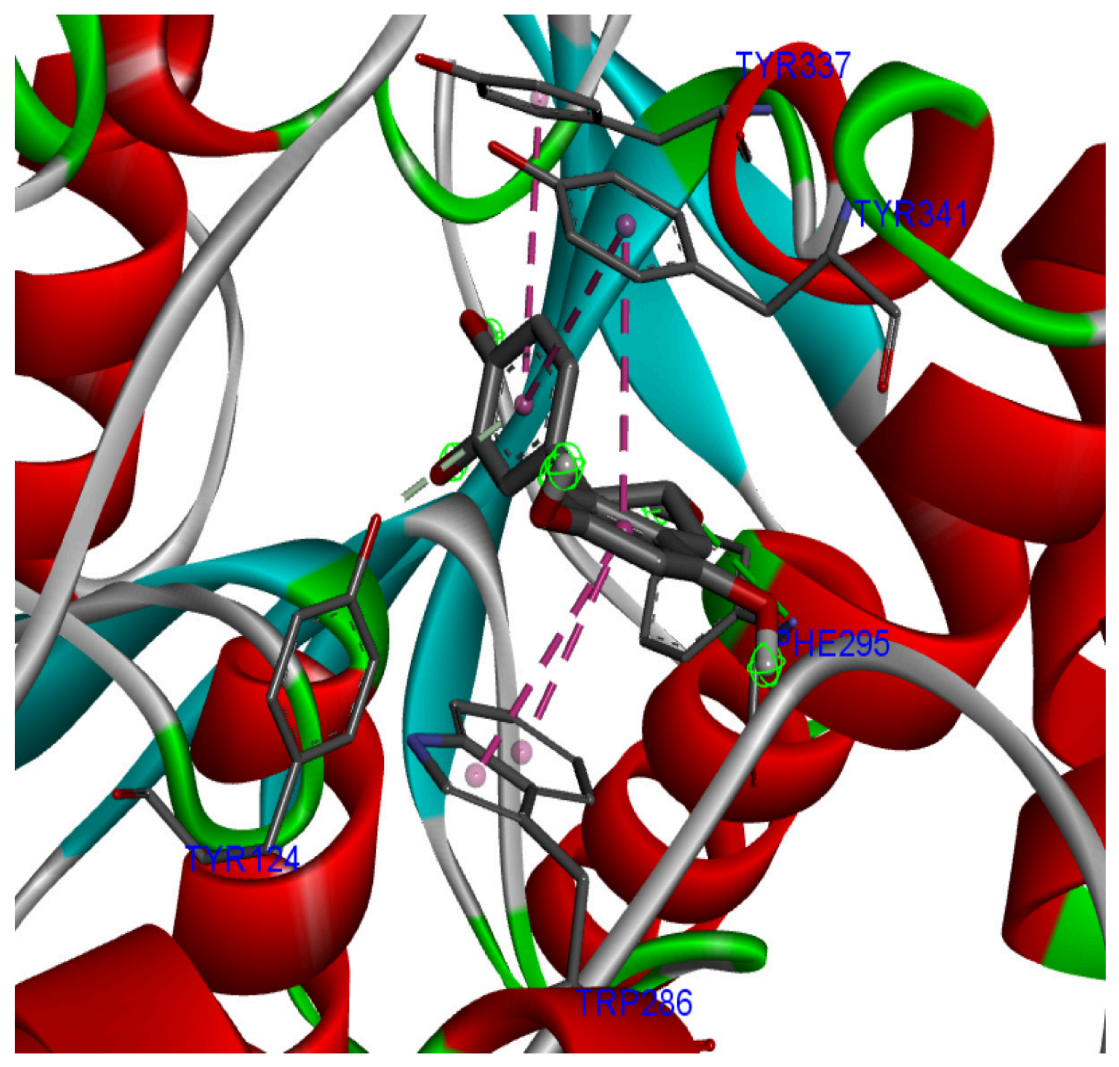
Gallic acid	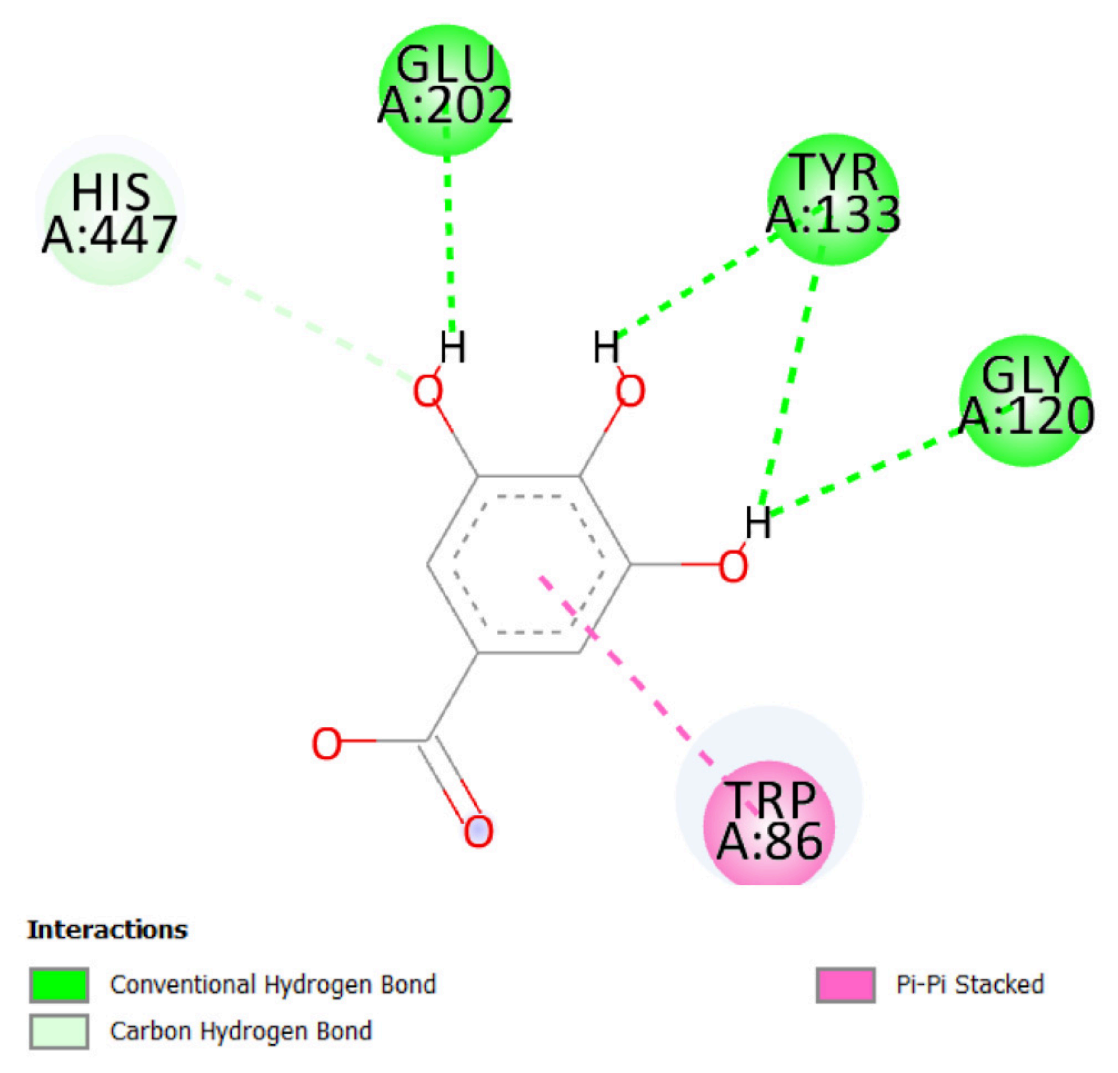	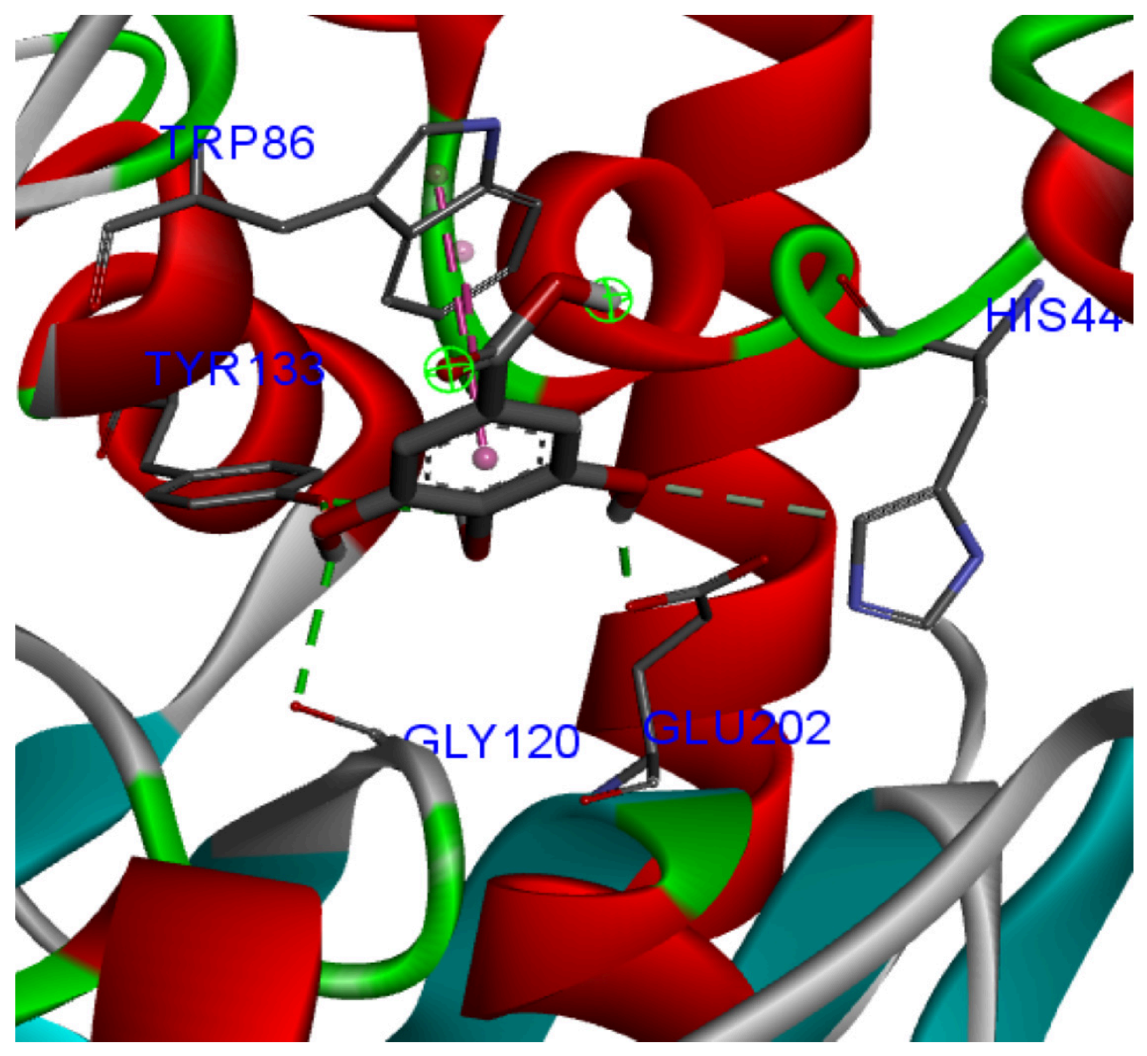

## Data Availability

All data generated or analyzed during this study are included in this published article.
